# Spaceflight-guided pharmacogenomics: a foundational analysis of pharmaceuticals and their responsive gene targets in the space environment

**DOI:** 10.3389/fphys.2026.1879838

**Published:** 2026-09-07

**Authors:** Theodore M. Nelson, Anurag Sakharkar, Julianna K. Rose, Claire E. Walter, Gresia L. Cervantes-Navarro, Caleb M. Schmidt, Richard Lin, Emma Alexander, Jiang Tao Zheng, Benjamin S. Glicksberg, Julian C. Schmidt, Sofia M. Etlin, Eliah Overbey, Li Shean Toh, Brinda K. Rana, Hemal H. Patel, Michael A. Schmidt, Christopher E. Mason

**Affiliations:** 1 Department of Physiology and Biophysics, Weill Cornell Medicine, Cornell University, New York, NY, United States; 2 BioAstra, Inc, New York, NY, United States; 3 Department of Microbiology & Immunology, Vagelos College of Physicians & Surgeons, Columbia University Irving Medical Center, New York, NY, United States; 4 Maldives Space Research Organization, Malé, Maldives; 5 School of Integrative Plant Sciences, Cornell University, Ithaca, NY, United States; 6 Department of Nutritional Sciences, Cornell University, Ithaca, NY, United States; 7 Polytechnic University of Guanajuato, GTO, Cortazar, Guanajuato, Mexico; 8 Sovaris Aerospace, Boulder, CO, United States; 9 Department of Systems Engineering, Colorado State University, Fort Collins, CO, United States; 10 Department of Biomedical Engineering, Cornell University, Ithaca, NY, United States; 11 Department of Communications, Cornell University, Ithaca, NY, United States; 12 Francis Lewis High School, Queens, NY, United States; 13 Hasso Plattner Institute for Digital Health, Icahn School of Medicine at Mount Sinai, New York, NY, United States; 14 Department of Biology and Society, Cornell University, Ithaca, NY, United States; 15 The HRH Prince Alwaleed Bin Talal Bin Abdulaziz Alsaud Institute for Computational Biomedicine, Weill Cornell Medicine, New York, NY, United States; 16 School of Pharmacy, University of Nottingham, Nottingham, United Kingdom; 17 Department of Psychiatry, University of California, San Diego, San Diego, CA, United States; 18 Department of Anesthesiology, University of California, San Diego, San Diego, CA, United States; 19 VA San Diego Healthcare System, San Diego, CA, United States; 20 Department of Medicine, Aerospace Medicine, University of Central Florida College of Medicine, Orlando, FL, United States; 21 Center for Aerospace and Extreme Environments Medicine, University of Central Florida College of Medicine, Orlando, FL, United States; 22 The Feil Family Brain and Mind Research Institute, Weill Cornell Medicine, New York, NY, United States; 23 WorldQuant Initiative for Quantitative Prediction, Weill Cornell Medicine, New York, NY, United States

**Keywords:** ADME, FAIR data, pharmaceuticals, pharmacogenomics, spaceflight, transcriptomics

## Abstract

Human spaceflight alters physiology in ways that can impact drug safety and efficacy, yet progress in this area has been hampered by a lack of integrated data. Our primary objective was to establish a Findable, Accessible, Interoperable, and Reproducible (FAIR) data curation framework by developing a novel database of pharmaceuticals used in spaceflight. This resource, compiled from publicly available literature, serves as a foundation for integrated pharmacological analysis. Using this resource, we demonstrate a data-driven framework for identifying pharmacologically-relevant, spaceflight-responsive genes by intersecting our drug catalog with available space-omics datasets. By focusing on the biological mechanisms perturbed by spaceflight, this approach provides a new avenue for pinpointing the most relevant changes within drug absorption, distribution, metabolism, and excretion (ADME) pathways. While necessarily limited by available tissue types, this work provides both the justification and a definitive starting point for spaceflight-guided pharmacogenomics. Ultimately, this establishes a foundational methodology to ensure the health and safety of future astronauts on long-duration missions to the Moon, Mars, and beyond.

## Graphical Abstract

**Figure:**
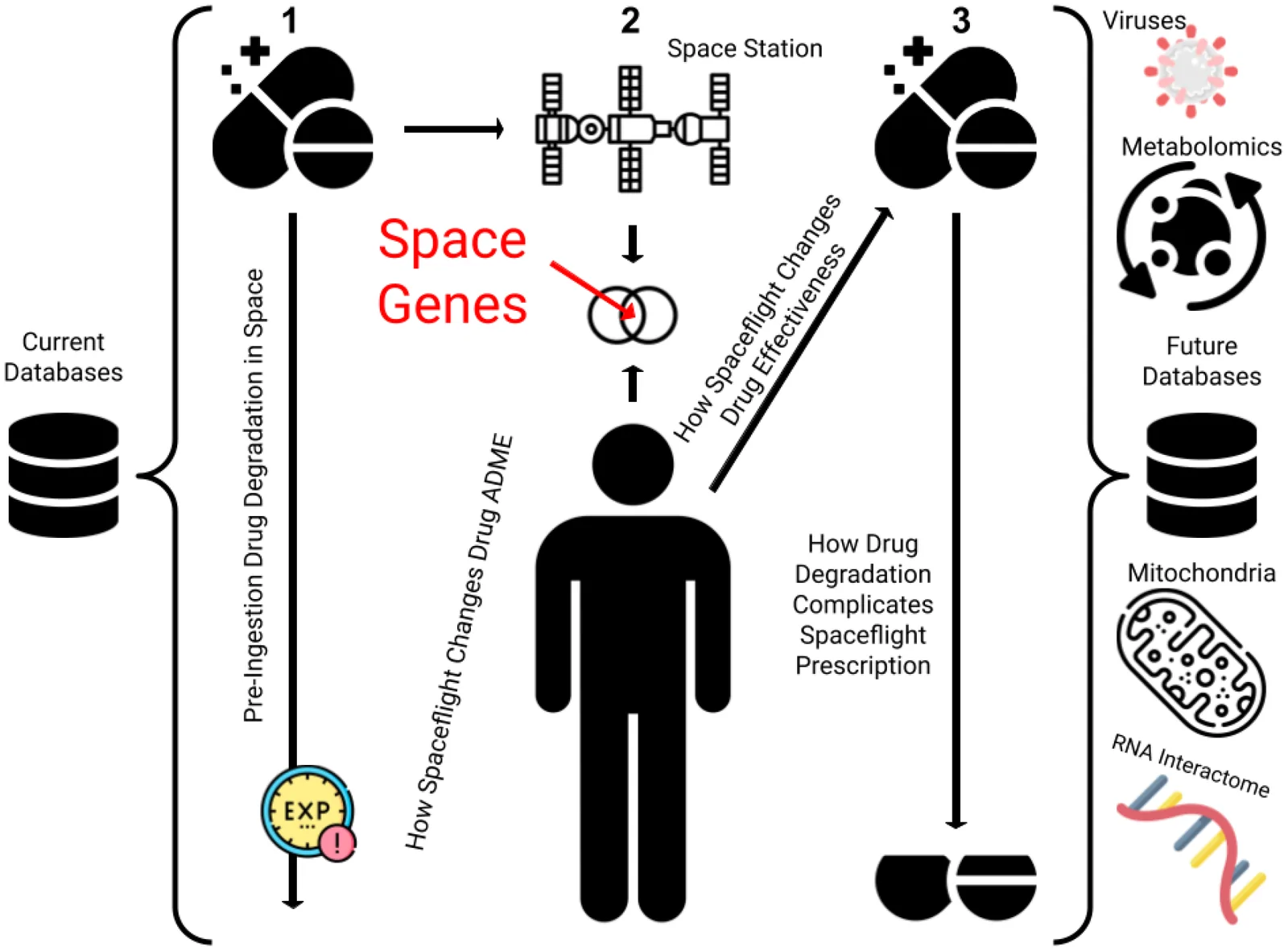

## Introduction

Human spaceflight subjects the body to a multitude of stressors, including microgravity, space radiation, and isolation, which collectively induce significant physiological adaptations. These changes include headward fluid shifts, immune system dysregulation, and altered metabolic function, often necessitating the use of pharmaceuticals to manage both acute spaceflight-induced conditions and pre-existing medical needs. As humanity prepares for long-duration missions to the Moon and Mars, and with the advent of commercial spaceflight involving a more diverse population of travelers, the importance of ensuring safe and effective medical treatments will only increase.

A higher resolution understanding of spaceflight-induced immune suppression has revealed an increased susceptibility to infections, necessitating the frequent use of pharmaceutical countermeasures. This reliance on medication, often involving the administration of multiple drugs simultaneously to treat complex or resistant conditions, significantly increases the risk of polypharmacy within spaceflight environments (Crucian et al., 2018). To date, the interactions between these necessary pharmaceutical interventions and individual astronaut genotype profiles remain largely unexplored.

Personalized medicine in space to date has focused on applying Earth-based pharmacogenomic principles, both from the perspective of short-term low earth-orbit travelers and future long-term human spaceflight missions (Pavez Loriè et al., 2021). The implication of current astronaut pharmaceutical usage in space, as well as a clinical perspective regarding the implications of drug metabolizer genotypes in relation to these, are well summarized by Blue et al (Blue et al., 2019). and Schmidt et al (Schmidt et al., 2019), respectively. While these reviews discuss the significant physiological effects of spaceflight, they do not systematically tie individualized spaceflight response to possible downstream pharmaceutical effects.

The previous ten years of human astronaut research has revealed individualized yet wide-ranging transcriptomic effects resulting from both short- and long-term low-earth orbit spaceflight (Garrett-Bakelman et al., 2019; Stratis et al., 2023). The development and expansion of the NASA GeneLab multi-omics database (Berrios et al., 2021), in combination with the Ames Life Science Data Archive (Scott et al., 2020), have represented a pioneering step in achieving Findable, Accessible, Interoperable, and Reproducible (FAIR) database curation (Wilkinson et al., 2016). Ten years ago, corresponding with the advent of the sequencing revolution, spaceflight-guided personalized medicine was first proposed (Schmidt and Goodwin, 2013). We believe that now is the appropriate time to again address this possibility, especially in the context of spaceflight-guided personalized medicine.

In our view, identifying spaceflight-responsive genes is a primary goal for fully understanding individualized transcriptomic responses to spaceflight. These are not limited to drug metabolizing genes, but can include all known drug-gene interactions dysregulated by spaceflight. The underlying mechanisms that determine drug response in spaceflight are complex, involving genotype, epigenetic markers, transcriptomic upregulation or downregulation, post-transcriptional modifications, post-translational tagging, and higher-order interactome dynamics, all further complicated by additional spaceflight-related variables, most prominently isolation, microgravity, and space radiation (Schmidt and Goodwin, 2013). Although current spaceflight datasets are limited by small astronaut cohort sizes, often generalizing potential intra-person variability to inter-person effects, we believe the application of established, Earth-generated pharmacogenomic knowledge-bases can immediately enhance personalized treatment in space. By anchoring astronaut care with proven drug-gene associations, we can better characterize the novel variability observed in flight, especially with the advent of in-flight sequencing technologies (McIntyre et al., 2016; Castro-Wallace et al., 2017). Moreover, the integration of FAIR data processes into future human spaceflight research practice will allow for seamless comparison to better inform clinical recommendations.

Within this framework, we will describe current field-specific databases related to spaceflight pharmaceuticals, introduce our own expanded knowledgebase, find “space genes”, and hypothesize the implications of these annotations on three critical drug processes: stability, metabolism, and efficacy/safety. Lastly, we will speculate on future exciting possibilities within pharmacogenomics multi-omics integrative analysis. This work emphasizes the novelty of bridging fragmented clinical reports with high-dimensional omics data to identify drug-response pathways vulnerable to spaceflight-induced dysregulation.

## Building a catalog of space-flown drugs

Pharmaceuticals are among the most critical countermeasures for maintaining astronaut health, given the range of physiological changes induced by microgravity, radiation, circadian disruption, and isolation. Nearly every space mission has documented the need for medications to treat both acute spaceflight-induced conditions (e.g., motion sickness, sleep disruption, infection) and ongoing medical needs. The most systematic evaluation to date, the Dose Tracker study, used an iOS-based logging system to record medication use on the International Space Station (ISS). Six astronauts and five ground-based controls provided detailed entries on drug name, dose, frequency, indication, perceived efficacy, and side effects, averaging 20.6 medication-use entries (dosing events) per subject per flight week. This study underscored the high frequency and diversity of pharmaceutical interventions in orbit, while also highlighting how little is systematically known about their safety and efficacy in the space environment.

Despite the obvious importance of pharmacology in human spaceflight, publicly available data are sparse and fragmented. Existing reports are scattered across NASA technical documentation, isolated mission studies, and individual reviews, making it difficult to integrate or compare findings. Few datasets adhere to FAIR principles, meaning that even when drug use is described, the data are not consistently curated, easily searchable, or linked to omics and physiological outcomes. This lack of standardized curation hampers the ability of researchers to analyze trends across missions or to connect pharmaceutical use with molecular and clinical phenotypes.

To address this gap, BioAstra, Inc. (https://www.bioastra.org/) hosted a space medicine data-mining competition to systematically catalog drug usage in spaceflight environments. Literature selection was based on a systematic search of PubMed and the NASA Technical Report Server using keywords including spaceflight, astronaut, and pharmaceutical. Through the review of fifty publications, 394 table entries corresponding to 218 unique drugs and drug combinations were compiled (**Supplementary Table 1**). This catalog more than doubles the entries compared to existing resources such as the Space Medicine Database (SPACELID) (Bioinformatics and Drug Design (BIDD) Group, 2024), which lists 106 medicines, and further maps these drugs to multi-omics targets. Some drugs were studied in tandem (e.g., scopolamine–dextroamphetamine; zolpidem–zaleplon), reflecting real-world prescribing practices. The most frequently mentioned medications across independent studies included zolpidem (12 studies), ampicillin (10), erythromycin (10), gentamicin (10), acetaminophen (8), promethazine (8), and tetracycline (8). This catalog represents, to our knowledge, the most comprehensive public resource on space-flown pharmaceuticals.

To place these findings in context, we compared our catalog against the official ISS Emergency Medical Procedures Manual (2016 edition), which lists the contents of both the “Convenience” and “Contingency” medical kits. Together, these kits provide roughly 200 pharmaceuticals intended to address anticipated in-flight medical needs ranging from infection and pain management to cardiovascular and psychiatric conditions. Of the 85 drugs explicitly listed in this NASA manual, only 45 were represented in our literature-derived database. This means nearly half of the drugs stocked in 2016 on the ISS have no publicly available literature regarding their use in spaceflight. Moreover, four drug combinations (sulfamethoxazole–trimethoprim, hydrocodone–acetaminophen, ciprofloxacin–dexamethasone, and tobramycin–dexamethasone) were present in the ISS kit but never described in published studies as in-flight combinations, despite each component drug appearing individually in the catalog. This discrepancy illustrates how operational knowledge within NASA medical kits has outpaced what is documented in the open scientific literature.

The contrast between actual ISS medical inventories and the published pharmacological evidence base underscores a critical gap. Current astronaut care often depends on terrestrial prescribing practices, with limited spaceflight-specific validation. To ensure safe and effective treatment on long-duration missions, future studies must integrate FAIR-compliant reporting of clinical pharmaceutical data (including dosing, frequency, indication, and side effects) alongside molecular and physiological measures. Only with standardized, openly accessible data can we begin to understand how the unique space environment alters pharmacokinetics and pharmacodynamics, ultimately enabling personalized and evidence-based pharmacological care for astronauts.

## Assessing the abiotic stability of pharmaceuticals in space

Due to aforementioned discrepancies within reporting standards, as well as the importance of accurate prescriptive treatment of astronauts, a brief discussion on drug degradation and expiration during spaceflight in an abiotic context is warranted.

To assess drug storage specifically, studies have most often examined the percentage of active pharmaceutical ingredient (API). Particularly striking are results indicating that medications flown aboard the ISS, including levothyroxine, promethazine, dextroamphetamine, and ciprofloxacin, did not meet regulatory requirements in terms of API content prior to their expiration date (Du et al., 2011). These medications, particularly the latter three, are highly light sensitive, and therefore likely saw significant increases in their degradation in space, potentially due to factors like increased radiation exposure that could compromise otherwise light-safe packaging over long durations (Du et al., 2011). In a separate study, API degradation and impurity were observed for some of the most commonly utilized drugs, including aspirin, ibuprofen, loratadine, modafinil, and zolpidem after storage aboard the ISS for 550 days (Wotring, 2016). Similar results have been shown for scopolamine, one of the most heavily prescribed drugs for astronauts (Wotring, 2015; Davis et al., 1993; Putcha and Cintrón, 1991; Tietze and Putcha, 1994; Traon et al., 1997), specifically to treat space adaptation syndrome and space motion sickness. Similar studies have been further summarized in Blue et. al., supporting the conclusion that the unique environment of spaceflight may accelerate the degradation of certain pharmaceuticals (Blue et al., 2019).

Nevertheless, contrary perspectives do exist, which note the relatively small difference in API, suggesting that this ultimately may not contribute to clinical outcomes (Reichard et al., 2023). Notably, all of the changes in drug stability aboard the ISS were within 10% of the changes seen at the Johnson Space Center (Reichard et al., 2023). These differences may be the result of confounding variables, including drug formulation and pre-flight storage conditions. In fact, in a study of different Vitamin B formulations within multivitamin brands, it was shown that B1 was changed significantly in one brand after storage on the ISS for 12 or 19 months, while the second brand demonstrated no statistically significant changes (Chuong et al., 2011). Further documentation and studies are necessary to both resolve this question, and evaluate downstream clinical effects.

## Curating observational data into pharmacogenomics knowledge for spaceflight

The foundation for personalized pharmacology in space begins with the translation of established terrestrial pharmacogenomic principles into astronaut care. Regulatory frameworks, such as the FDA’s table of pharmacogenomic biomarkers in drug labeling, and clinical implementation guidelines from the Clinical Pharmacogenetics Implementation Consortium (CPIC), already identify high value gene-drug pairs where genomic variation predicts efficacy or toxicity. Applying these standards to astronaut populations would allow pre-flight screening for common functional variants in key ADME genes (e.g., CYP2D6, CYP2C19, VKORC1, SLCO1B1, UGT1A1), establishing baseline metabolizer phenotypes before launch. This approach has been repeatedly suggested by terrestrial clinicians and space medicine experts as a pragmatic first step to reduce the risk of therapeutic failure or adverse drug reactions in the unique and resource-limited environment of spaceflight.

However, relying solely on terrestrial regulatory labeling or population-based guidelines is insufficient for the dynamic environment of spaceflight. The FDA updating process is designed for broad population safety, making it inherently conservative. Similarly, while CPIC provides the gold standard for clinical guidelines, its recommendations are based on large-scale terrestrial studies that do not account for space-specific variables, such as radiation exposure or microgravity-induced fluid shifts. Astronauts may experience “phenoconversion”, where environmental stressors transiently alter a drug-metabolizing phenotype, creating a discrepancy between their germline genotype and their functional metabolic capacity that current terrestrial guidelines cannot anticipate.

To address this, we need a mechanism to capture and evaluate space-specific pharmacogenomic data. The space biology community has already established robust infrastructure for FAIR data that can support this research. The NASA Open Science Data Repository and Ames Life Sciences Data Archive (ALSDA) has standardized omics data processing across more than 700 spaceflight experiments. Similarly, the Space Omics and Medical Atlas (SOMA) has extended this model to civilian spaceflight. These platforms do not replace clinical validation; rather, they serve as the essential discovery engine, allowing researchers to aggregate small, heterogeneous datasets to generate high-confidence hypotheses about how spaceflight alters drug processing.

Given the centrality of safe drug use for astronaut health, we envision a consortium modeled on CPIC, employing similar rigor in evidence review but optimized for the realities of astronaut medicine, small cohorts, longitudinal monitoring, and the integration of multi-omics data. Such a group could utilize the signals derived from OSDR, SOMA, and other large-scale integrated space biology studies to curate evidence and provide graded recommendations. This ensures that while data ingestion remains fast and FAIR-compliant in the research domain, clinical guidance remains evidence-based, bridging the gap between raw omics signals and operational medical safety. BioAstra’s annual SOMA Summit already convenes international experts around multi-omics datasets and could serve as a venue for iterative review and release of curated spaceflight pharmacogenomic guidance. Establishing this infrastructure now would ensure that astronaut pharmacology transitions from reactive, terrestrial extrapolation to proactive, data-driven personalization as humanity embarks on longer missions to the Moon and Mars.

## Spaceflight-related data curation practices

To systematically track and predict drug efficacy in space, robust data infrastructure is required. The ability to link pharmacogenomic profiles to clinical outcomes relies on properly curated, FAIR databases. Space-related research possesses a high barrier to entry, due to limited spaceflight opportunities and data availability, resulting in an increased frequency of interdisciplinary collaborations, and decreased statistical power within studied patient cohorts (Amselem and Eyal, 2022). Due to these limitations, a digital ecosystem of databases has emerged to preserve information in a FAIR manner (Wilkinson et al., 2016). Database curation and management are maintained by relevant space agencies and supported by international cohorts of subject-matter experts. The aim of these platforms is to discover spaceflight-induced phenotypes across astronaut missions and research studies, reducing future biological threats to long-term human spaceflight through assessment and targeted application of countermeasures.

Even though these databases have been established to produce valuable insights from previous missions, the input is sparse. A few notable studies within the previous decade include a comprehensive examination of the astronaut microbiome (Morrison et al., 2021), along with the NASA Twins Study, which provided a paired-ground spaceflight experiment, although limited to one replicate, analyzing changes pre-, in- and post-flight (Garrett-Bakelman et al., 2019). Even though these provided more comprehensive portraits of baseline physiological effects, they did not extensively characterize spaceflight pharmacological-transcriptomic interactions.

We next describe three field-specific databases in terms of their capacity for integration: the NASA Technical Report Server (https://ntrs.nasa.gov/), the NASA Open Science Data Repository (https://genelab.nasa.gov/), and the Space Omics and Medical Atlas (https://soma.weill.cornell.edu/) (SOMA).

The first of these, the NASA Technical Report Server, is a collection of NASA-affiliated or funded datasets, conference publications, and scientific articles. Although expansive in breadth, the diversity of source material makes the information difficult to integrate. In contrast, the NASA GeneLab repository focuses on a narrower set of data types, related to multi-omics sequencing and allows for uniform processing and integration. This platform is now integrated with the Ames Life Sciences Data Archive (ALSDA). First published in 1994, these combined platforms contain a diverse array of population, microscopy, transcriptomic, genetic, metabolomic, and proteomic data. Experiments have been conducted in a wide variety of organisms as well as microgravity simulation systems. Notably, more than 375 rodent studies have been carried out to understand the mechanisms that affect organisms in conditions ranging from radiation exposure to microgravity treatments. In terms of actual spaceflight studies, these repositories contain over 700 experiments conducted aboard the International Space Station (ISS), the NASA/MIR space station, Bion/Cosmos, Gemini, Biosatellites, Apollo, Skylab and the Russian Foton missions. SOMA, hosted by Weill Cornell Medicine’s Epigenomics Core Facility, is a specialized platform designed to facilitate the analysis of omics data related to space research. It currently houses data from the Inspiration4 mission. Ultimately, study-specific databases such as the SOMA Browser, which catalogs the data from SpaceX’s civilian missions, can provide an even deeper layer of integration and visualization support, among diverse and occasionally novel sample collection profiles.

These three platforms represent distinct tiers of data accessibility. The NASA TRS functions as a vast but unstructured archive, NASA OSDR operationalizes FAIR principles, and SOMA provides a specialized, integrative environment for rapid analysis. Currently, the collective body of spaceflight pharmacogenomics knowledge most closely resembles the first of these categories, extensive in scope but fragmented across disparate literature reviews and isolated technical reports. While generalist sequencing repositories (such as the European Nucleotide Archive (https://www.ebi.ac.uk/ena/browser/)) have implemented metadata standards to track experimental conditions, strict privacy protocols for government astronauts have historically prevented the inclusion of granular medication logs in these public datasets.

This gap prevents the direct linking of omics profiles to specific dosing events. To overcome this fragmentation, we must adopt an integrative strategy: utilizing our manually curated catalog of space-flown pharmaceuticals to bridge the divide between scattered clinical reports and high-dimensional omics data. By systematically mapping these pharmaceutical countermeasures to their biological targets, we can bypass the lack of direct clinical annotation and begin to identify which drug-response pathways are most vulnerable to spaceflight-induced dysregulation.

## Identifying cell-type specific spaceflight-driven adverse drug effects

As discussed in the Introduction, genes constitute the foundational unit of biological analysis, currently employed in genetic, transcriptomic and proteomic analyses. Utilizing the catalog of space-flown drugs, we determined that 17 of them (fluorouracil, atorvastatin, codeine, ibuprofen, mercaptopurine, nitrofurantoin, omeprazole, ondansetron, phenytoin, sertraline, and the aminoglycoside antibiotics amikacin, gentamicin, kanamycin, neomycin, paromomycin, streptomycin and tobramycin) are part of CPIC level A gene-drug pairs, meaning there is strong evidence that genetic variation has a clinically meaningful impact on drug response and that actionable prescribing guidelines already exist based on genotype.

Within this review, we take a novel approach and look beyond genetics. To do that, we first define a set of related space genes by defining a set of related genes impacted by spaceflight-flown pharmaceuticals. The list of drugs known to have been applied in space was matched with the drug-gene interaction database DGIdb (Cannon et al., 2023) in order to generate a gene set potentially affected by in-flight drug usage. After collating the results from both database versions, we found that out of the 218 unique drugs within our space medicine database, 190 had interactions with 772 unique genes, for a total of 2,318 interactions (**Supplementary Table 2**).

**Figure 1:**
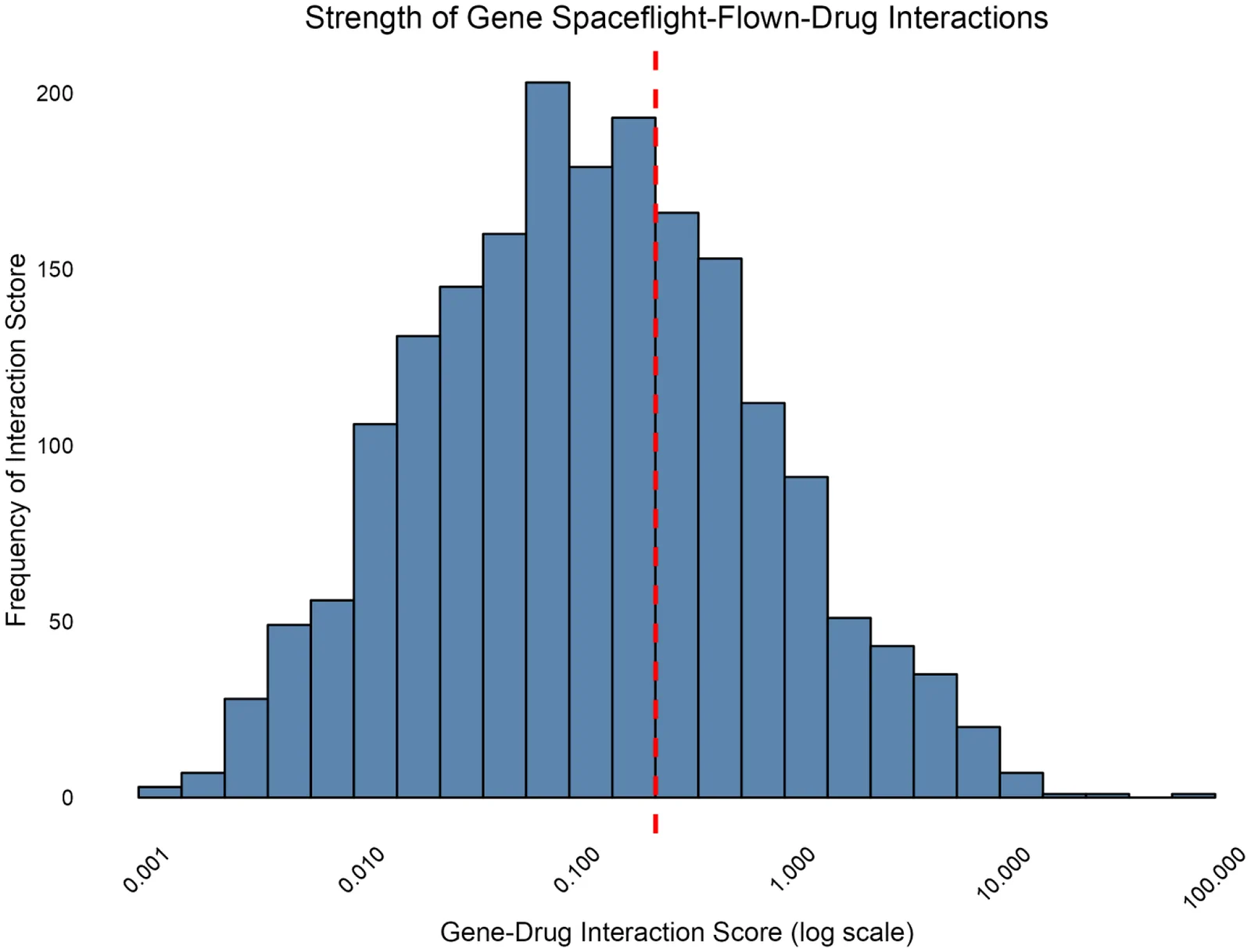
Strength of gene spaceflight-flown-drug interactions. A line at the border demarcates an interaction strength of 0.2 (note the log scale on the x-axis).

The newer DGIdb database version ranks drug-gene interactions via a scoring metric. This is collated based on evidence scores, related to the number of supporting publications and sources, the ratio of average known gene partners for all drugs to the known partners for the given drug, and the ratio of average known drug partners for all genes to the known partners for the given gene (Cannon et al., 2023). We utilize an arbitrary yet lenient cutoff of 0.2 for discussion purposes within this review. A total of 681 ranked interactions pass this cutoff, while 377 interactions do not have an interaction score assigned (**Figure 1**). Unsurprisingly, these genes are, as a collection, most associated with response to chemical stressors (**Supplementary Figure 1**), further indicating their important role within the mechanistic action of the selected drugs. Downstream effects could result from many different potential mechanisms related to these genes.

Although their specific relevance to the spaceflight environment has not yet been established, the genes mentioned above represent important targets for drugs commonly used in space. To demonstrate a framework for identifying this relevance, we intersected our master list of 772 potential drug-gene targets with two distinct, publicly available space-omics datasets as case studies. Due to fundamental differences in their cell type, experimental platform (true vs. simulated microgravity), and biological measurement (transcriptome vs. proteome), these datasets are not expected to show significant overlap. Instead, they serve to illustrate a methodology for deriving testable hypotheses from the limited data currently available.

A convergent approach would be to define spaceflight-perturbed genes utilizing RNA-sequencing assays, which capture gene expression across conditions. For this purpose, we selected the closest public analogue of human spaceflight available within the NASA GeneLab repository; namely, a study of human induced pluripotent stem cell-derived cardiomyocytes, which utilized a well-controlled ISS spaceflight perturbation (n=3 per group), as opposed to microgravity simulation devices. This transcriptomic dataset followed the standard NASA GeneLab normalization pipeline, which includes log2 transformation and quantile normalization. Although cell types specific to drug metabolizing cells within the liver would have been preferable, we selected the most relevant dataset available for this application.

To indicate drugs whose mechanistic actions may be perturbed by spaceflight, we intersected the set of 772 unique genes associated with spaceflight-applied drugs with the set of differentially expressed genes, available within the **Supplementary Materials** of (Wnorowski et al., 2019). There were 48 genes whose mechanistic perturbation intersected with current spaceflight-flown drug associated genes: ABCA1, ABL1, ADRB3, APOE, ATF4, B4GALT2, BAX, CHRM3, CREBBP, CYP1B1, CYP2D6, CYP3A43, ERCC1, FOS, FTO, GCLC, GGT1, GLP1R, GNAS, GSR, GSTP1, GTF2B, HRH1, HSPA5, HSPA8, IMPDH1, JUN, JUNB, MECP2, MVK, PIK3R2, PLOD1, PRDX1, PTH1R, RAD52, RPS19, SCN9A, SDHB, SLC19A1, SLC23A2, SLC29A2, SOD1, STS, TGM2, TMEM167A, UGT1A4, WRN, and ZBTB22. The gene ontology for each of these genes is described in **Supplementary Table 3**.

Collectively, these influenced the following 36 drugs: acyclovir, alendronate sodium, ascorbic acid, aspirin, atorvastatin calcium trihydrate, brompheniramine maleate, cefotaxime sodium, cetirizine hydrochloride, chlorzoxazone, cinnarizine, ciprofloxacin, copper chloride, cyclizine, dexamethasone, dimenhydrinate, diphenhydramine, doxycycline anhydrous, fluorouracil, gentamicin, hydrogen peroxide, levofloxacin anhydrous, melatonin, mercaptopurine, metoclopramide hydrochloride, ofloxacin, ondansetron hydrochloride, progestin, quetiapine fumarate, selenomethionine, sulfamethoxazole, tetracycline, thrombin, triprolidine, vitamin D, vitamin E.

As a second case study, we utilized a proteomics dataset from a different experimental context. When cells from the Caco-2 cell line, a human male colorectal adenocarcinoma, were treated with simulated microgravity conditions, researchers, using label-free shotgun proteomics, found a host of proteins with differential abundances at both 48 and 72 hours of microgravity. Capturing a total of 6,109 proteins with this assay, the authors found 62 with differential abundance. When comparing this proteomic set to the 772 genes associated with space-flown medicines, there was a total overlap of three genes: APOA1, interacting with furosemide and progestin, CDH17, interacting with dexamethasone, and DPEP1, interacting with cilastatin and dexamethasone.

## Interpreting results and addressing limitations

**Figure 2:**
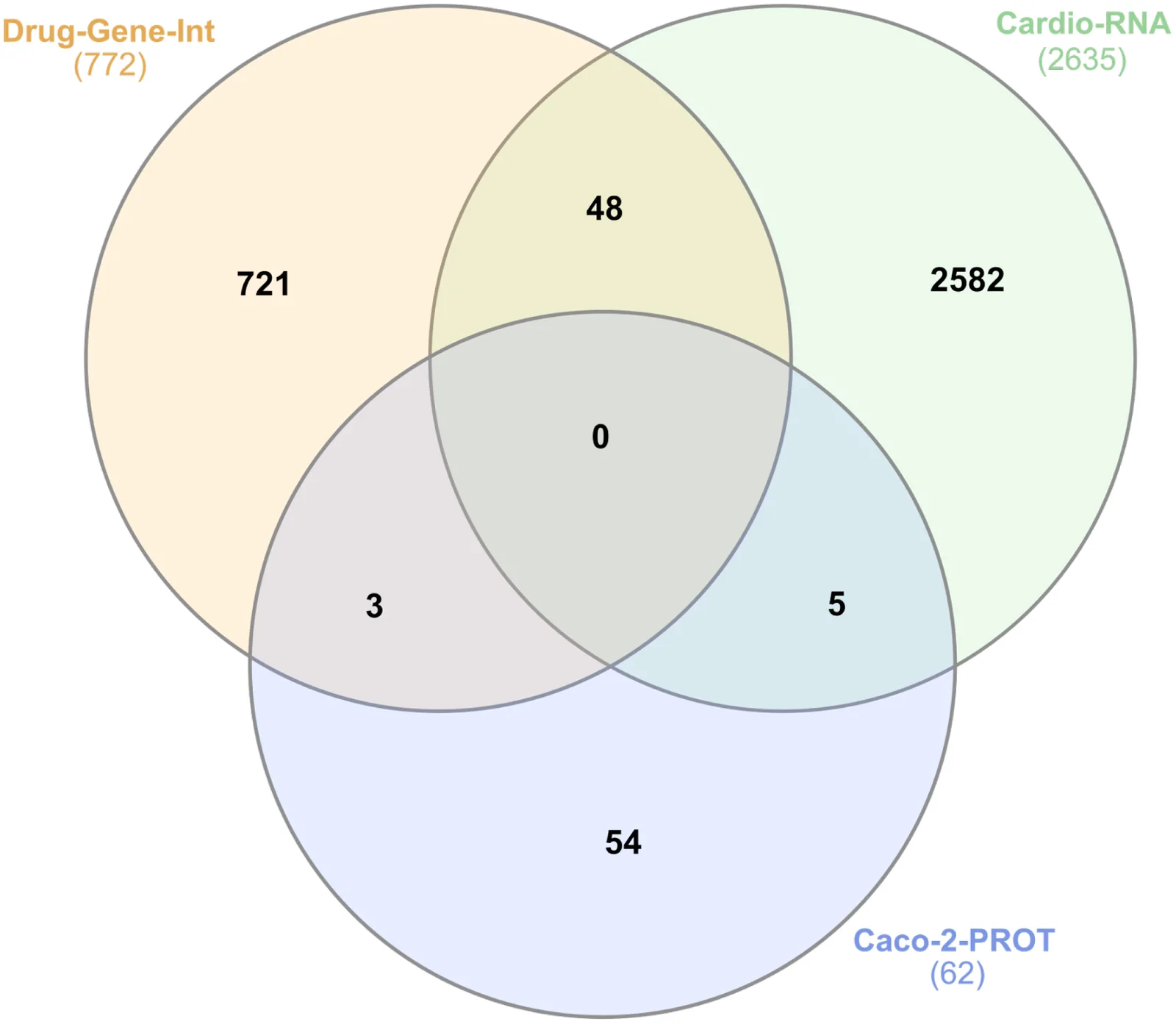
Intersection analysis. Intersection between published spaceflight-flown drug-gene interactions, differentially expressed gene sets from ISS-flown cardiomyocytes, and differentially expressed protein sets from simulated-microgravity Caco-2 cells. Chart produced with Interactivenn.

When comparing between the differential sets associated with either the gene-drug interaction database, the proteomic gene set, and the transcriptomic gene set, we find limited overlap between any of the indicated space genes (**Figure 2**). As such, we recognize the preliminary nature of these predictions. The lack of overlap between the gene sets derived from these two analyses is not surprising and is, in fact, an expected outcome given the disparate nature of the source data. The discrepancies arise from several key factors: 1) fundamental differences in tissue type (cardiomyocytes vs. colorectal adenocarcinoma cells), which have inherently distinct gene and protein expression profiles due to tissue-specific expression; 2) different biological assays (RNA-sequencing vs. proteomics), as changes in transcript levels do not always correlate directly with protein abundance; and 3) different experimental platforms (true spaceflight aboard the ISS vs. ground-based simulated microgravity), which introduce different environmental variables.

This highlights a critical limitation in the current field and underscores the need for future experiments using more physiologically relevant models, particularly for pharmacogenomic studies. As suggested by prior research, models of microgravity-induced differential expression in human liver tissue would be invaluable, as the liver is the primary site of drug metabolism. Furthermore, to move beyond tissue-specific transcriptomics, future research must characterize the full spectrum of pharmacological variables unique to long-duration spaceflight. This methodical approach to drug prescribing—focused on the intersection of drug–gene, drug–drug, drug–herb, drug–food, drug–nutrient, and drug-space interactions—has been described as the “constrained drug interactome” (Schmidt et al., 2025). While these six categories (**Table 1**) represent the primary controllable (or operating) elements, they provide a necessary framework for navigating the complex and variable effects of the space environment on pharmaceutical efficacy and safety (Schmidt et al., 2026).

**Table 1:** The six primary categories of the constrained drug interactome.

Category	Description of constraint
Drug–Gene	Influence of germline polymorphisms and in-flight transcriptomic shifts on drug metabolism.
Drug–Drug	Pharmacokinetic interactions resulting from in-flight polypharmacy.
Drug–Herb	Interactions between pharmaceuticals and herbal supplements or bioactive compounds.
Drug–Food	Effects of spaceflight diet and altered gastric emptying on drug bioavailability.
Drug–Nutrient	Depletion or supplementation of vitamins and minerals impacting enzymatic pathways.
Drug–Space	Direct effects of radiation, microgravity, and fluid shifts on drug stability and physiology.

Crucially, the intersection of abiotic and biotic factors creates a non-linear risk profile. For example, a 10% abiotic loss in API potency, if combined with a 50% biotic decrease in CYP450 metabolic activity, could lead to a cumulative risk where the effective dose delivered to the target tissue is outside of the therapeutic window, potentially resulting in toxicity or therapeutic failure that terrestrial models cannot anticipate.

## A systematic driver: the impact of immune dysregulation on absorption, distribution, metabolism, and excretion

**Figure 3:**
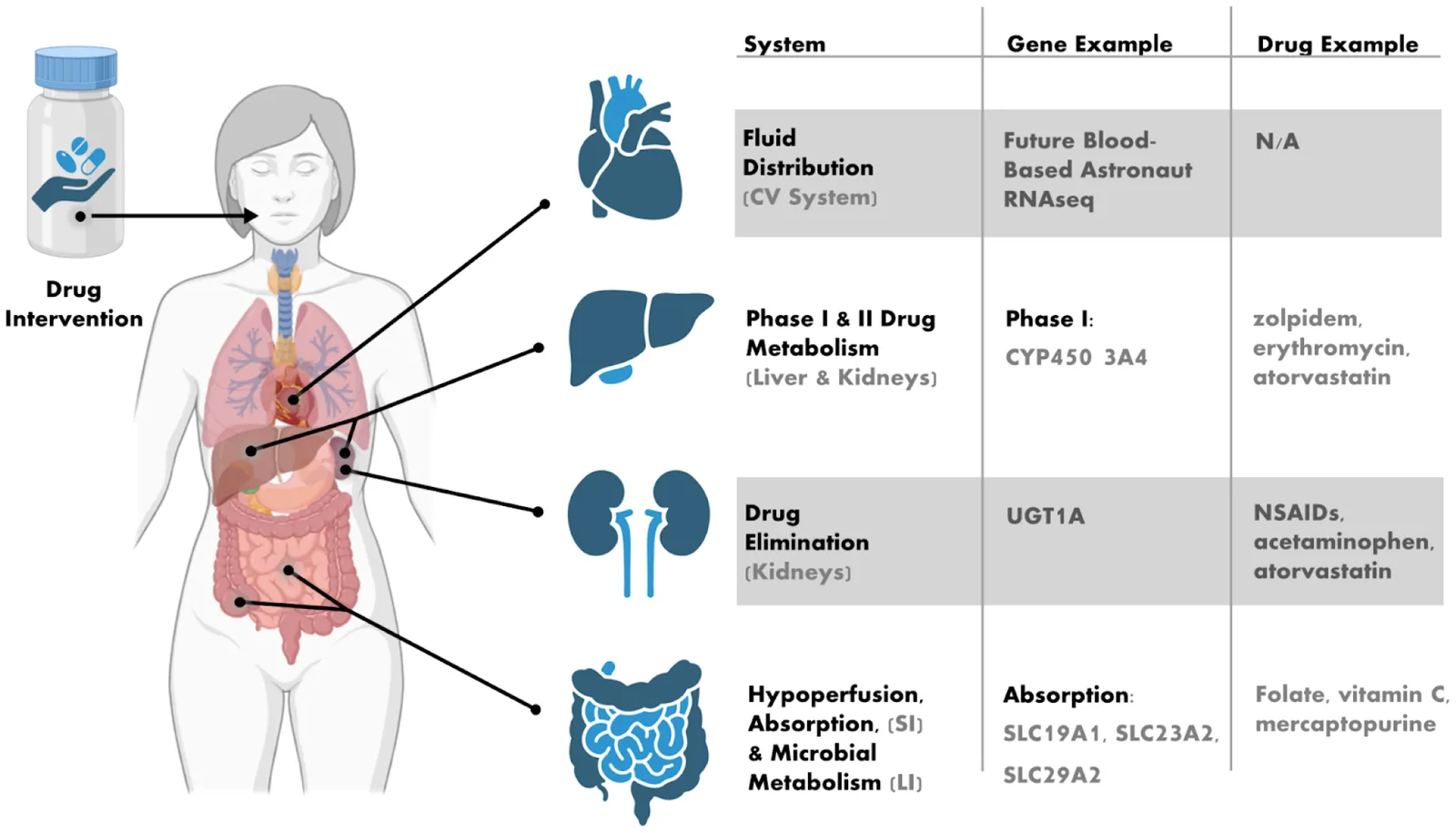
ADME model of altered drug metabolism in space. CV, cardiovascular; SI, small intestine; LI, large intestine.

While direct pharmacokinetic data in astronauts is limited, established terrestrial molecular underpinnings allow us to hypothesize how spaceflight-induced physiological changes, such as immune dysregulation, might alter ADME (**Figure 3**). This immune dysregulation is not merely a standalone phenomenon; it has profound, direct implications for the molecular machinery of pharmacokinetics (**Table 2**). On Earth, inflammatory cytokines are known to systemically remodel drug processing at the genetic and protein levels. For instance, elevated levels of Interleukin-6 (IL-6), a common marker in inflammatory states, directly suppress the hepatic expression and activity of crucial drug-metabolizing enzymes like CYP3A4 and CYP2C19 (Evers et al., 2013; Lanchote et al., 2015), which can dramatically increase patient exposure and toxicity risk for a number of common medications or result in treatment inefficacy. This cytokine-mediated downregulation extends to Phase II enzymes like UGTs and key transporters such as OATPs, broadly decreasing the liver’s ability to take up and clear drugs. Furthermore, inflammatory signals from immune cells can alter the expression of efflux transporters like P-glycoprotein in the gut and the blood-brain barrier, simultaneously altering the oral absorption of some drugs while changing the central nervous system penetration of others.

**Table 2:** Summary of frequently documented pharmaceuticals and representative ADME considerations.

Rank	Drug name	Studies	Target	ADME relevance	Observed effect
1	Zolpidem	12	CYP3A4	Metabolism (Phase I)	Inhibit. by Erythromycin
2	Ampicillin	10	–	Absorption/Stability	Acid-labile degradation
3	Erythromycin	10	CYP3A4	Metabolism (Phase I)	Inhibitor of 3A4
4	Gentamicin	10	–	Excretion (Renal)	Nephrotoxicity risk
5	Acetaminophen	8	–	Metabolism (Phase II)	Hydroquinone byproduct
6	Promethazine	8	–	Distribution/Efficacy	4.8% Sedation in flight
7	Tetracycline	8	–	Excretion (Renal)	EATC byproduct toxicity

“Studies” indicates the number of independent publications documenting the use or analysis of the drug in a spaceflight context. Detailed gene-drug mappings for 48 identified space-responsive targets are provided in **Supplementary Table 3**.

Beyond direct transcriptional regulation, systemic inflammation alters the physiological landscape through which drugs are distributed and cleared. During acute inflammatory responses, increased vascular permeability and shifts in protein synthesis can lead to hypoalbuminemia. This has a dual effect on pharmacokinetics: hydrophilic drugs (e.g., *β*-lactams) face a larger volume of distribution, creating a risk of underdosing, while highly protein-bound drugs (e.g., phenytoin, warfarin) have a higher free fraction, increasing the risk of toxicity. Organ function is also impacted; IL-6-mediated hemodynamic changes can reduce the glomerular filtration rate (GFR), impairing the clearance of renally-eliminated drugs. This systemic activation also has consequences for modern therapeutics, as heightened phagocytic activity by macrophages can accelerate the sequestration of nanomedicines and liposomal drugs, shifting their distribution away from intended targets and shortening their circulation time. Therefore, the well-documented immune shifts in astronauts likely create a dynamic and unpredictable pharmacokinetic background, which must be considered in concert with direct genetic changes in ADME pathways. Against this backdrop of systemic immune-driven effects, we will now examine the direct evidence from space-omics data for spaceflight-induced alterations in the genes governing each stage of the ADME process.

### Physiological extrapolation: hepatic and renal considerations

While this study utilizes cardiomyocyte and Caco-2 models, the liver and kidneys remain the primary sites for drug clearance and metabolism. The identified 48 genes, while expressed in the case study tissues, often have well-documented roles in hepatic and renal drug processing. For example, the upregulation of UGT1A4 and downregulation of CYP2D6 suggests a systematic shift in drug-conjugation and oxidation capacity that likely mirrors terrestrial inflammatory responses. Future work utilizing organ-on-a-chip or human liver organoids will be essential to bridge these tissue-specific observations to clinical astronaut outcomes.

Having identified a preliminary set of 48 candidate spaceflight-responsive genes, we will now explore their potential mechanistic implications for pharmacology. Using the classic ADME framework, we can begin to form data-driven hypotheses about how the unique environment of spaceflight may alter drug processing, efficacy, and safety at a molecular level.

## Effects of spaceflight on the genes of absorption

Once ingested during spaceflight, a drug encounters a myriad of biologically-related challenges to carry out its function. Orally ingested drugs, as opposed to injected drugs, are initially subject to delayed gastric emptying. While this primarily alters the timing of drug absorption (Tmax), it may also affect the stability of acid-labile compounds due to prolonged exposure to the gastric environment. Furthermore, bioavailability can be impacted by fluid shift-associated hypoperfusion of the GI tract and changes in the expression of GI enzymes and transporters. As an example, we observed three solute transporter genes (SLC19A1, SLC23A2, SLC29A2, for folate, vitamin C, and nucleosides, respectively) within our set of spaceflight-responsive genes. Both SLC19A1 and SLC23A2 demonstrated selective spaceflight-related downregulation, whereas SLC29A2 was upregulated. The differential expression of these transporters suggests a direct mechanism by which spaceflight could alter not only nutrient uptake but also the absorption of drugs that rely on these pathways.

Once absorbed, the spaceflight environment significantly alters drug distribution for orally ingested as well as injected drugs. These factors are myriad and individual response is variable, relating to the following: 1) fluid shift: the headward fluid shift increases natriuresis and diuresis, impairs lymph flow and lymphatic drainage, decreases thirst, and increases evaporation through the skin and lungs, 2) fluid redistribution from plasma to extracellular volume; and thereafter from extracellular volume to intracellular volume, 3) decreased plasma volume, which can lead to increased drug concentration, 4) altered protein binding expression and plasma concentration, 5) endothelial dysfunction, and 6) changes in hepatic blood flow.

## Effects of spaceflight on the genes of distribution

The distribution of drugs is significantly altered by the headward fluid shifts, decreased plasma volume, and altered protein binding that occur in space. Furthermore, systemic inflammation, a known effect of spaceflight, can drive changes in drug distribution. For example, increased vascular permeability can lead to a larger volume of distribution for hydrophilic drugs, while inflammatory-driven hypoalbuminemia can increase the free fraction of highly protein-bound drugs, creating risks of both under- and over-dosing.

## Effects of spaceflight on the genes of drug metabolism

Drug metabolism is particularly susceptible to spaceflight-induced changes, which can be affected by: 1) expression and, thus, enzyme concentration changes of the phase I and phase II drug metabolism system, 2) pharmacometabolomic signatures associated with a certain drug intervention or metabolic state, and 3) food components and other xenobiotics as a result of polypharmacy and other environmental exposures that are also known to be inducers and inhibitors of the Cytochrome P450 (CYP450) pathway. Within our set of spaceflight-responsive genes, we observed differential expression of CYP1B1, CYP2D6, and CYP3A43, which are all components of the CYP450 pathway. This superfamily of enzymes performs various oxidation reactions that are the dominant metabolic process in phase I metabolism, occurring mostly within the liver. Within the ISS-flown cardiomyocytes, CYP1B1 and CYP2D6 demonstrated downregulation during spaceflight, whereas CYP3A43 was upregulated.

Pharmaceutical inhibition or induction of CYP450 enzymes is the leading cause of drug-drug interactions, as altering the function of one enzyme can inhibit metabolism of another drug processed by the same enzyme. By this mechanism, if the volume or function of primary CYP450 enzymes was altered significantly in space, it could have detrimental effects on certain drugs. Multiple studies have observed changes in CYP450 expression during spaceflight, and one study of rats flown on Spacelab 3 reported a 50% decrease in hepatic cytochrome P450 content (Merrill et al., 1987). Future studies should determine whether these enzymes are similarly affected in human astronauts and what the downstream implications are for drug metabolism.

Liver homeostasis also relies in part on glutathione, an important compound for protecting against oxidative stress that additionally serves as a crucial molecule in drug conjugation. We noted upregulation of GCLC and GGT1, important for glutathione production, in our analysis. These results are consistent with current hypotheses regarding increased levels of oxidative stress within spaceflight environments and suggest a potential alteration in the body’s capacity for drug conjugation.

## Effects of spaceflight on the efficacy of a drug at the target

Separate from questions of drug absorption and processing is whether the mechanistic action with the primary target of interest is disrupted during spaceflight. In particular, the example of promethazine was particularly extreme from a clinical perspective. Pharmacodynamics were significantly altered on space shuttle flights, with 4.8% of astronauts experiencing sedation upon administration of this drug in comparison to the 60-73% who experienced sedation in ground controls (Bagian and Ward, 1994). While the precise molecular cause of this drastic shift is not yet known, it underscores the potential for spaceflight to profoundly alter drug efficacy.

Instead of relying solely on clinical observations, we can also use our data-driven framework to select strong interaction partners and predict potential mechanistic interruptions. To demonstrate this, we have selected the interaction with the greatest score in the DGIdb, between the MVK gene and alendronate sodium (**Supplementary Table 2**). The human MVK gene encodes for mevalonate kinase, a key enzyme in the isoprenoid pathway, which is vital to numerous bodily processes including cholesterol synthesis (Buhaescu and Izzedine, 2007; Chiarella et al., 2022; Anthony et al., 2009). This pathway is a target for statins and bisphosphonates like alendronate, a drug of interest for preventing bone loss in astronauts. Our analysis of the cardiomyocyte data showed that MVK is upregulated in spaceflight. As a result, we can hypothesize that this interaction could have different pharmacodynamics within the spaceflight environment, potentially altering the efficacy of alendronate as a countermeasure. Integrating further pharmacogenomic resources will allow better clinical recommendations regarding this drug and help determine if the change in gene expression is based on specific regulation of MVK or upstream pathway effects.

## Effects of spaceflight on drug excretion and abiotic degradation products

Finally, once drugs have exerted their primary effect, they may be metabolized, degraded, and/or excreted, depending on the drug. However, with regard to spaceflight-specific effects on the degradation of drugs within *in vivo* biological systems, there is a severe lack of data. Therefore, we must also consider the potential biological effects of abiotic drug degradation, where the byproducts themselves may pose a health risk. Novel degradation products have not been identified within studies of commonly used space pharmaceuticals, but several known degradation products warrant consideration.

Excretion is predominantly handled by the kidneys, involving renal epithelial cells. To date, very few space-omics studies have focused on human renal cell models. Our finding that UGT1A4—a key component of the glucuronidation pathway that is also expressed in the kidney—was upregulated in cardiomyocytes offers a preliminary clue that excretory pathways could be affected (Benoit-Biancamano et al., 2009; Wang et al., 2014). Beyond biological excretion, we must also consider the health risks posed by the abiotic degradation products of pharmaceuticals.

Zolpidem, a drug frequently used aboard the ISS as within our database, has four primary degradation products that have been studied: zolpacid, oxozolpidem, zolpaldehyde, and zolpyridine. These compounds were measured after photolysis and oxidation, two methods based on liquid formulations of zolpidem (Malesevic et al., 2014). While many of these zolpidem degradation products have the same basic structure as zolpidem, which consists of an imidazopyridine ring (Malesevic et al., 2014), they may have different toxicity, bioavailability, and therapeutic effects than zolpidem (Malesevic et al., 2011). This may be because the degradation products of zolpidem have highly variable polarity, zolpacid being highly polar, due to carboxylic groups, and zolpaldehyde being highly nonpolar (Malesevic et al., 2011). In terms of abiotic stability, it is important to note, that zolpidem has been deemed stable in its solid form, with degradation occurring more often in solution (Malesevic et al., 2014). Thus, the degradation products from this drug may not be as much of a concern as some other compounds aboard the ISS. Yet, there is still some indication of degradation of solid zolpidem under the stressors of heat and visible light to around 15% degradation (Souri, 2012).

Another prominent medication, as determined via the comparison between our compiled database and the 2016 ISS medical kit, was erythromycin. As erythromycin degrades, it is converted into ERY-H2O through dehydration of the compound (Ashraf et al., 2021; Fan et al., 2009). While ERY-H2O has primarily been studied in an ecological context, this pathway is relevant to the closed-loop water recycling systems of spacecraft. ERY-H2O contributes to bacterial erythromycin resistance despite the degradation of the compound itself (Fan et al., 2009; Majer, 1981), leading to declining drug efficacy, which would be problematic if this medication were to be brought along longer-duration space missions.

Regarding both previous drugs, there are also potential interactome interactions between these effector complexes. Zolpidem is known to rely on the CYP3A4 gene for the majority of its metabolism (Yoon et al., 2021). Erythromycin itself is an inhibitor of CYP3A4, suggesting that if these drugs were given together, metabolism of zolpidem could be inhibited, leading to increased plasma concentrations and prolonged sedation rather than the typical clearance rates (Orlando et al., 2003). These kinds of interactions within compound polypharmacy should also be considered in future space medicine prescription.

Acetaminophen is a drug with several studied impurities and degradation products which have the potential for negative human health impacts, indicating the importance of understanding spaceflight-related drug metabolism and degradation. The principal storage degradation product is p-aminophenol; hydroquinone has additionally been reported as a by-product of acetaminophen under advanced oxidative conditions (Qutob et al., 2022). Hydroquinone is a key product of study, being a benzenediol compound with debated human health impacts (Schwartz et al., 2023). More specifically, the carcinogenic nature of hydroquinone has been contested, mainly due to its use as a topical agent for skin lightening. Most studies of hydroquinone carcinogenicity have been conducted in rat models, such as a 2005 study indicating that hydroquinone can build up in the body, as it is not initially metabolized by the liver, which can increase the abundance of carcinogenic metabolites and lead to nephrotoxicity (Westerhof and Kooyers, 2005). However, a variety of human studies have not found significant correlation between hydroquinone and cancer (McGregor, 2007). Given the debated human health risks associated with hydroquinone, and the elevated oxidative conditions of the space environment, monitoring for its potential formation from acetaminophen aboard the space station or future long-duration space vessels is warranted, although such degradation has not yet been observed in spaceflight. Furthermore, the EPA lists a number of noncancerous effects such as increased tinnitus, nausea, vomiting, and abdominal cramps upon ingestion of this compound (https://www.epa.gov/sites/default/files/2016-09/documents/hydroquinone.pdf). As such, monitoring acetaminophen degradation in space will be increasingly important as flight durations increase.

Furthermore, promethazine degradation products have been studied, primarily using oxidative degradation methods. These products include formaldehyde, acetaldehyde, and dimethylamine, 10-methylphenothiazine, phenothiazine, 3H-phenothiazine-3-one, phenothiazine 5-oxide, promethazine 5-oxide, and 7-hydroxy-3Hphenothiazine-3-one (Underberg, 1978). Such products will be important to consider and monitor along long duration space missions. Formaldehyde is an endogenous genotoxin that can form DNA and DNA-protein crosslinks, and the Fanconi anemia DNA-repair pathway is required to protect cells from these lesions. Accordingly, formaldehyde accumulation can contribute to genome instability, particularly in Fanconi-pathway-deficient cells, although it remains unknown whether promethazine degradation during spaceflight would generate biologically relevant formaldehyde exposure (Reingruber and Pontel, 2018). Not only that, but ingestion of this compound in its liquid state has been associated with numerous health conditions in humans, including metabolic acidosis, proteinuria, abdominal pain, nausea, and dizziness (Kim et al., 2011). While degradation may not be driven by thermal oxidation or promethazine in solution aboard the ISS, and the body possesses endogenous mechanisms to metabolize trace formaldehyde, the degradation products shown via this methodology should continue to be examined when considering medication for space missions to avoid cumulative toxicity.

The last drug that will be analyzed in depth in this section is tetracycline, a broad- spectrum antibiotic (Shutter and Akhondi, 2023). The main degradation products of tetracycline are 5a,6-anhydrotetracycline hydrochloride (ATC), 4-epitetracycline hydrochloride (ETC), and 4-epi-anhydro-tetracycline hydrochloride (EATC) (Halling-Sørensen et al., 2002). ETC and EATC have been shown to have negative impacts on human health; EATC, for example, can induce Fanconi syndrome, a proximal tubular disorder characterized by glycosuria, aminoaciduria, proteinuria, hypophosphatemia, hypokalemia, and metabolic acidosis (Gross, 1963; Montoliu et al., 1981). With such problematic effects, the degradation products of tetracycline become a human health risk for long duration space missions.

Many degradation products have been studied in aquatic environments, where antibiotics have accumulated due to their increased prevalence. Thus, the degradation products arising from these environments may differ from those aboard the ISS or spacecraft for longer duration space missions. However, the fact that the observed degradation products that have been seen have the potential to negatively impact human health indicates the need to conduct further studies of drug degradation products and pathways in the space environment to protect astronauts and future space travelers. Ultimately, maintaining 90% potency, a target within Mehta and Bhayani (2017), for medications, likely, remains a future challenge for many classes of drugs within space pharmacogenomics, given the likely variable impacts of long-term spaceflight on drug degradation (Mehta and Bhayani, 2017).

## Limitations

This analysis represents a preliminary step toward spaceflight-guided pharmacogenomics and is subject to several key limitations. First, our database of space-flown drugs is derived from publicly available literature and technical reports, which may not capture the full scope of internal space agency medical records or the granular details of in-flight polypharmacy. This underscores the need for an explicit call to action for space agencies to release de-identified medication logs to the NASA Open Science Data Repository (OSDR) to facilitate more robust pharmacological analysis. Second, the omics datasets used as case studies—ISS-flown cardiomyocytes and simulated-microgravity Caco-2 cells—are disparate and do not reflect the primary drug-metabolizing tissue of the liver. The resulting lack of overlap in our comparative analysis underscores the urgent need for human hepatic models in future space research. Third, the interpretation of ADME changes based on immune dysregulation remains partially speculative, relying on terrestrial molecular underpinnings that may be further modified by radiation or long-duration spaceflight stressors. Finally, we must distinguish between abiotic drug degradation (chemical stability in storage) and biotic metabolism; while this review addresses both, a comprehensive model of their interaction is currently hindered by sparse in-flight longitudinal data.

## Future directions

Future research should prioritize high-resolution, longitudinal monitoring of astronaut pharmacokinetic profiles alongside multi-omics data curation. A clear roadmap to clinical application for the 48 identified genes involves a three-step validation process. First, the gene-drug associations identified here should be prioritized for *in vitro* testing in hepatic organoids under microgravity. Second, these findings should be correlated with longitudinal, de-identified medication logs from space agencies to confirm observed efficacy shifts and address the potential for cumulative toxicity. Finally, verified associations should be integrated into a modified Clinical Pharmacogenetics Implementation Consortium (CPIC) framework that incorporates spaceflight-specific phenoconversion factors. Specifically, addressing abiotic drug stability through inflight analytical monitoring—potentially utilizing portable mass spectrometry as a countermeasure for long-duration missions—will be critical to ensure therapeutic efficacy.

## Conclusion: a new framework for pharmacogenomics-guided spaceflight

In this review, we have addressed the critical need for a data-driven approach to pharmacology in space. We began by tackling the lack of FAIR data curation, presenting a novel and comprehensive database of space-flown pharmaceuticals. Using this foundation, we demonstrated a proof-of-concept framework for identifying pharmacologically-relevant, spaceflight-responsive genes by intersecting our drug catalog with available space-omics data. Finally, we used the resulting candidate genes to generate the first mechanistic hypotheses, grounded in the ADME model, for how drug stability, metabolism, and efficacy may be altered during spaceflight.

While our analysis has focused on transcriptomics to understand the dynamic changes induced by spaceflight, this is only one component of a complete pharmacogenomics strategy. The full promise of personalized medicine in space will be realized by integrating these in-flight molecular portraits with the established principles of terrestrial pharmacogenomics. This involves pre-flight genomic screening for known pharmacogenetic polymorphisms in critical ADME genes (e.g., CYP2D6, CYP2C19) to identify an astronaut’s baseline metabolizer phenotype (e.g., poor, intermediate, normal, or ultrarapid metabolizer). By applying established clinical guidelines from bodies like the CPIC, we can proactively identify individuals at higher risk for adverse drug reactions or therapeutic failure. Furthermore, understanding an astronaut’s baseline genotype is crucial for predicting the potential impact of phenoconversion, where the co-administration of an inhibitor drug can make a normal metabolizer respond like a poor metabolizer, a significant risk in the polypharmacy-common environment of spaceflight.

The path forward, therefore, requires a dual approach: pre-flight genomic profiling to understand an astronaut’s inherent genetic predispositions, combined with in-flight transcriptomic and multi-omic monitoring to understand how their body is dynamically responding to the space environment. This integrated strategy will be especially critical with the rise of civilian spaceflight, where participants will present with more diverse genetic backgrounds and pre-existing medical conditions. To make this vision a reality, future research must prioritize the collection of omics data from more physiologically relevant models, particularly those reflecting hepatic function, and adhere to FAIR data principles to enable the development of robust, evidence-based prescribing guidelines.

Ultimately, spaceflight likely has a profound and variable effect on the pharmacokinetics and pharmacodynamics of drugs. The work presented here, our foundational database, analytical framework, and initial data-driven hypotheses, provides a starting point for a new paradigm in space medicine. By systematically building upon this foundation, we can move from reactive problem-solving to proactive, personalized, and predictive pharmacology, ensuring the health and safety of the next generation of space explorers on long-duration missions to the Moon, Mars, and beyond.

## Data Availability

The original contributions presented in the study are included in the article/**Supplementary Material**. Further inquiries can be directed to the corresponding author.

## References

[B1] Amselem S. Eyal S. (2022). The blood-brain barrier in space: Implications for space travelers and for human health on earth 2, 931221. doi: 10.3389/fddev.2022.931221

[B2] Anthony J. R. Anthony L. C. Nowroozi F. Kwon G. Newman J. D. Keasling J. D. (2009). Optimization of the mevalonate-based isoprenoid biosynthetic pathway in Escherichia coli for production of the anti-malarial drug precursor amorpha-4,11-diene 11, 13–19. doi: 10.1016/j.ymben.2008.07.007 18775787

[B3] Ashraf A. Liu G. Yousaf B. Arif M. Ahmed R. Irshad S. . (2021). Recent trends in advanced oxidation process-based degradation of erythromycin: Pollution status, eco-toxicity and degradation mechanism in aquatic ecosystems 772, 145389. doi: 10.1016/j.scitotenv.2021.145389 33578171

[B4] Bagian J. P. Ward D. F. (1994). A retrospective study of promethazine and its failure to produce the expected incidence of sedation during space flight 34, 649–651. doi: 10.1002/j.1552-4604.1994.tb02019.x 9766972

[B5] Benoit-Biancamano M.-O. Adam J.-P. Bernard O. Court M. H. Leblanc M.-H. Caron P. . (2009). A pharmacogenetics study of the human glucuronosyltransferase UGT1A4 19, 945–954. doi: 10.1097/FPC.0b013e3283331637 19890225 PMC6177227

[B6] Berrios D. C. Galazka J. Grigorev K. Gebre S. Costes S. V. (2021). NASA GeneLab: Interfaces for the exploration of space omics data 49, D1515–D1522. doi: 10.1093/nar/gkaa887 33080015 PMC7778922

[B7] Bioinformatics and Drug Design (BIDD) Group (2024). Spacelid: Space Medicine Database. Available online at: https://bidd.group/spacelid/index.html (Accessed June 7, 2026).

[B8] Blue R. S. Bayuse T. M. Daniels V. R. Wotring V. E. Suresh R. Mulcahy R. A. . (2019). Supplying a pharmacy for NASA exploration spaceflight: Challenges and current understanding 5, 14. doi: 10.1038/s41526-019-0075-2 31231676 PMC6565689

[B9] Buhaescu I. Izzedine H. (2007). Mevalonate pathway: A review of clinical and therapeutical implications 40, 575–584. doi: 10.1016/j.clinbiochem.2007.03.016 17467679

[B10] Cannon M. Stevenson J. Stahl K. Basu R. Coffman A. Kiwala S. . (2023). Dgidb 5.0: Rebuilding the drug–gene interaction database for precision medicine and drug discovery platforms. Nucleic Acids Res. 52, D1227–D1235. doi: 10.1093/nar/gkad1040 37953380 PMC10767982

[B11] Castro-Wallace S. L. Chiu C. Y. John K. K. Stahl S. E. Rubins K. H. McIntyre A. B. R. . (2017). Nanopore DNA sequencing and genome assembly on the International Space Station 7, 18022. doi: 10.1038/s41598-017-18364-0 29269933 PMC5740133

[B12] Chiarella E. Nisticò C. Di Vito A. Morrone H. L. Mesuraca M. (2022). Targeting of mevalonateisoprenoid pathway in acute myeloid leukemia cells by bisphosphonate drugs 10, 1146. doi: 10.3390/biomedicines10051146 35625883 PMC9138592

[B13] Chuong M. C. Prasad D. LeDuc B. Du B. Putcha L. (2011). Stability of vitamin B complex in multivitamin and multimineral supplement tablets after space flight 55, 1197–1200. doi: 10.1016/j.jpba.2011.03.030 21515013

[B14] Crucian B. E. Choukèr A. Simpson R. J. Mehta S. Marshall G. Smith S. M. . (2018). Immune system dysregulation during spaceflight: Potential countermeasures for deep space exploration missions 9, 1437. doi: 10.3389/fimmu.2018.01437 30018614 PMC6038331

[B15] Davis J. Jennings R. Beck B. (1993). Comparison of treatment strategies for space motion sickness 29, 587–591. doi: 10.1016/0094-5765(93)90074-7 11541638

[B16] Du B. Daniels V. R. Vaksman Z. Boyd J. L. Crady C. Putcha L. (2011). Evaluation of physical and chemical changes in pharmaceuticals flown on space missions 13, 299–308. doi: 10.1208/s12248-011-9270-0 21479701 PMC3085701

[B17] Evers R. Dallas S. Dickmann L. J. Fahmi O. A. Kenny J. R. Kraynov E. . (2013). Critical review of preclinical approaches to investigate cytochrome p450-mediated therapeutic protein drug-drug interactions and recommendations for best practices: A white paper 41, 1598–1609. doi: 10.1124/dmd.113.052225 23792813

[B18] Fan C. Lee P. K. H. Ng W. J. Alvarez-Cohen L. Brodie E. L. Andersen G. L. . (2009). Influence of trace erythromycin and erythromycin-H2O on carbon and nutrients removal and on resistance selection in sequencing batch reactors (SBRs) 85, 185–195. doi: 10.1007/s00253-009-2201-7 19727707 PMC2765652

[B19] Garrett-Bakelman F. E. Darshi M. Green S. J. Gur R. C. Lin L. Macias B. R. . (2019). The NASA Twins Study: A multidimensional analysis of a year-long human spaceflight 364. doi: 10.1126/science.aau8650 30975860 PMC7580864

[B20] Gross J. M. (1963). Fanconi syndrome (adult type) developing secondary to the ingestion of outdated tetracycline. Ann. Intern. Med. 58 (3), 523–528. doi: 10.7326/0003-4819-58-3-523 13950771

[B21] Halling-Sørensen B. Sengeløv G. Tjørnelund J. (2002). Toxicity of tetracyclines and tetracycline degradation products to environmentally relevant bacteria, including selected tetracycline-resistant bacteria 42, 263–271. doi: 10.1007/s00244-001-0017-2 11910453

[B22] Kim K.-H. Jahan S. A. Lee J.-T. (2011). Exposure to formaldehyde and its potential human health hazards 29, 277–299. doi: 10.1080/10590501.2011.629972 22107164

[B23] Lanchote V. L. Almeida R. Barral A. Barral-Netto M. Marques M. P. Moraes N. V. . (2015). Impact of visceral leishmaniasis and curative chemotherapy on cytochrome P450 activity in Brazilian patients 80, 1160–1168. doi: 10.1111/bcp.12677 25940755 PMC4631188

[B24] Majer J. (1981). *In vitro* induction of resistance to erythromycin by its metabolite 19, 628–633. doi: 10.1128/AAC.19.4.628 7247386 PMC181491

[B25] Malesevic M. Zivanovic L. Protic A. Jovic Z. (2011). Multiobjective optimization approach in evaluation of chromatographic behaviour of zolpidem tartrate and its degradation products 74, 197–208. doi: 10.1007/s10337-011-2064-930311153

[B26] Malesevic M. Zivanovic L. Protic A. Radisic M. Lausevic M. Jovic Z. . (2014). Stress degradation studies on zolpidem tartrate using LC-DAD and LC-MS methods 26, 81–96. doi: 10.1556/AChrom.26.2014.1.8

[B27] McGregor D. (2007). Hydroquinone: An evaluation of the human risks from its carcinogenic and mutagenic properties 37, 887–914. doi: 10.1080/10408440701638970 18027166

[B28] McIntyre A. B. R. Rizzardi L. Yu A. M. Alexander N. Rosen G. L. Botkin D. J. . (2016). Nanopore sequencing in microgravity 2, 16035. doi: 10.1038/npjmgrav.2016.35 28725742 PMC5515536

[B29] Mehta P. Bhayani D. (2017). Impact of space environment on stability of medicines: Challenges and prospects 136, 111–119. doi: 10.1016/j.jpba.2016.12.040 28068518

[B30] Merrill A. H. Jr. Wang E. Jones D. P. Hargrove J. L. (1987). Hepatic function in rats after spaceflight: effects on lipids, glycogen, and enzymes. Am. J. Physiol. 252 (2 Pt 2), R222-6. doi: 10.1152/ajpregu.1987.252.2.R222 3812760

[B31] Montoliu J. Carrera M. Darnell A. Revert L. (1981). Lactic acidosis and Fanconi's syndrome due to degraded tetracycline. Br. Med. J. (Clin. Res. Ed.) 283 (6306), 1576–1577. doi: 10.1136/bmj.283.6306.1576-a 6796174 PMC1508026

[B32] Morrison M. D. Thissen J. B. Karouia F. Mehta S. Urbaniak C. Venkateswaran K. . (2021). Investigation of spaceflight induced changes to astronaut microbiomes 12, 659179. doi: 10.3389/fmicb.2021.659179 34149649 PMC8207296

[B33] Orlando R. Piccoli P. De Martin S. Padrini R. Palatini P. (2003). Effect of the CYP3A4 inhibitor erythromycin on the pharmacokinetics of lignocaine and its pharmacologically active metabolites in subjects with normal and impaired liver function 55, 86–93. doi: 10.1046/j.1365-2125.2003.01718.x 12534644 PMC1884183

[B34] Pavez Loriè E. Baatout S. Choukér A. Buchheim J.-I. Baselet B. Dello Russo C. . (2021). The future of personalized medicine in space: From observations to countermeasures 9, 739747. doi: 10.3389/fbioe.2021.739747 34966726 PMC8710508

[B35] Putcha L. Cintrón N. M. (1991). Pharmacokinetic consequences of spaceflight 618, 615–618. doi: 10.1111/j.1749-6632.1991.tb27292.x 11537657

[B36] Qutob M. Hussein M. A. Alamry K. A. Rafatullah M. (2022). A review on the degradation of acetaminophen by advanced oxidation process: pathway, by-products, biotoxicity, and density functional theory calculation. RSC Adv. 12 (29), 18373–18396. doi: 10.1039/d2ra02469a 35799916 PMC9214717

[B37] Reichard J. F. Phelps S. E. Lehnhardt K. R. Young M. Easter B. D. (2023). The effect of long-term spaceflight on drug potency and the risk of medication failure 9, 35. doi: 10.1038/s41526-023-00271-6 37147378 PMC10163248

[B38] Reingruber H. Pontel L. B. (2018). Formaldehyde metabolism and its impact on human health 9, 28–34. doi: 10.1016/j.cotox.2018.07.00142574925

[B39] Schmidt M. A. Goodwin T. J. (2013). Personalized medicine in human space flight: Using omics based analyses to develop individualized countermeasures that enhance astronaut safety and performance 9, 1134–1156. doi: 10.1007/s11306-013-0556-3 24273472 PMC3825629

[B40] Schmidt M. A. Schmidt C. M. Schmidt J. C. Meydan C. Afshinnekoo E. Beheshti A. . (2026). Systemic elevation of gut derived p-cresol during one year in space: Implications for brain, heart, bone, liver, and microbiome in astronauts Special Issue: Bioconvergence: A New Frontier for Understanding and Enhancing Human Adaptations to Extreme Environments (in press).

[B41] Schmidt M. A. Schmidt C. M. Goodwin T. J. (2019). “ Pharmacogenomics in spaceflight,” in Handbook of Space Pharmaceuticals. Eds. Pathak Y. Araujo´ Dos Santos M. Zea L. ( Springer International Publishing), 1–39. doi: 10.1007/978-3-319-50909-9_26-1

[B42] Schmidt M. A. Schmidt J. C. Schmidt C. M. (2025). “ Precision medicine in human spaceflight: Applying systems thinking to principles, concepts, and methods of assessment,” in Fundamentals of Space Medicine and Clinical Technology. Eds. Waisberg E. Ong J. Lee A. G. ( Academic Press, Elsevier). doi: 10.1016/B978-0-443-32904-3.00014-542574925

[B43] Schwartz C. Jan A. Zito P. M. (2023). “ Hydroquinone,” in Statpearls (Treasure Island (FL): StatPearls Publishing).

[B44] Scott R. T. Grigorev K. Mackintosh G. Gebre S. G. Mason C. E. Del Alto M. E. . (2020). Advancing the integration of biosciences data sharing to further enable space exploration 33, 108441. doi: 10.1016/j.celrep.2020.108441 33242404

[B45] Shutter M. C. Akhondi H. (2023). “ Tetracycline,” in Statpearls (Treasure Island (FL): StatPearls Publishing). 31751095

[B46] Souri E. (2012). Validated stability indicating HPLC method for determination of zolpidem in the presence of its degradation products 3, 13–17. doi: 10.2174/2210289201203010013

[B47] Stratis D. Trudel G. Rocheleau L. Pelchat M. Laneuville O. (2023). The transcriptome response of astronaut leukocytes to long missions aboard the International Space Station reveals immune modulation 14, 1171103. doi: 10.3389/fimmu.2023.1171103 37426644 PMC10324659

[B48] Tietze K. J. Putcha L. (1994). Factors affecting drug bioavailability in space 34, 671–676. doi: 10.1002/j.1552-4604.1994.tb02022.x 8083399

[B49] Traon A. P.-L. Saivin S. Soulez-LaRivière C. Pujos M. Güell A. Houin G. (1997). “ Chapter 4 pharmacology in space: pharmacotherapy,” in Advances in Space Biology and Medicine, vol. 6. ( Elsevier), 93–105. doi: 10.1016/S1569-2574(08)60079-9 9048135

[B50] Underberg W. (1978). Oxidative degradation of pharmaceutically important phenothiazines I: Isolation and identification of oxidation products of promethazine 67, 1128–1131. doi: 10.1002/jps.2600670826 671251

[B51] Wang Z. Wong T. Hashizume T. Dickmann L. Z. Scian M. Koszewski N. J. . (2014). Human UGT1A4 and UGT1A3 conjugate 25-hydroxyvitamin D3: Metabolite structure, kinetics, inducibility, and interindividual variability 155, 2052–2063. doi: 10.1210/en.2013-2013 24641623 PMC4020929

[B52] Westerhof W. Kooyers T. J. (2005). Hydroquinone and its analogues in dermatology – a potential health risk 4, 55–59. doi: 10.1111/j.1473-2165.2005.40202.x 17166200

[B53] Wilkinson M. D. Dumontier M. Aalbersberg I. J. g.-i. Appleton G. Axton M. Baak A. . (2016). The FAIR Guiding Principles for scientific data management and stewardship 3, 160018. doi: 10.1038/sdata.2016.18 26978244 PMC4792175

[B54] Wnorowski A. Sharma A. Chen H. Wu H. Shao N.-Y. Sayed N. . (2019). Effects of spaceflight on human induced pluripotent stem cell-derived cardiomyocyte structure and function 13, 960–969. doi: 10.1016/j.stemcr.2019.10.006 31708475 PMC6915842

[B55] Wotring V. E. (2015). Medication use by U.S. crewmembers on the International Space Station 29, 4417–4423. doi: 10.1096/fj.14-264838 26187345

[B56] Wotring V. E. (2016). Chemical Potency and Degradation Products of Medications Stored Over 550 Earth Days at the International Space Station. AAPS J. 18 (1), 210–216. doi: 10.1208/s12248-015-9834-5 26546565 PMC4706284

[B57] Yoon S. Jeong S. Jung E. Kim K. S. Jeon I. Lee Y. . (2021). Effect of CYP3A4 metabolism on sex differences in the pharmacokinetics and pharmacodynamics of zolpidem 11, 19150. doi: 10.1038/s41598-021-98689-z 34580385 PMC8476623

