# Supplementary material for: Spaceflight-guided pharmacogenomics: a foundational analysis of pharmaceuticals and their responsive gene targets in the space environment
Source: Front Physiol. 2026 Sep 7;17:1879838. doi: 10.3389/fphys.2026.1879838 (PMC13590397; doi:10.3389/fphys.2026.1879838)
Supplement: Supplementary file 1 [file DataSheet1.pdf]

# Supplementary Material

## SUPPLEMENTARY FIGURES

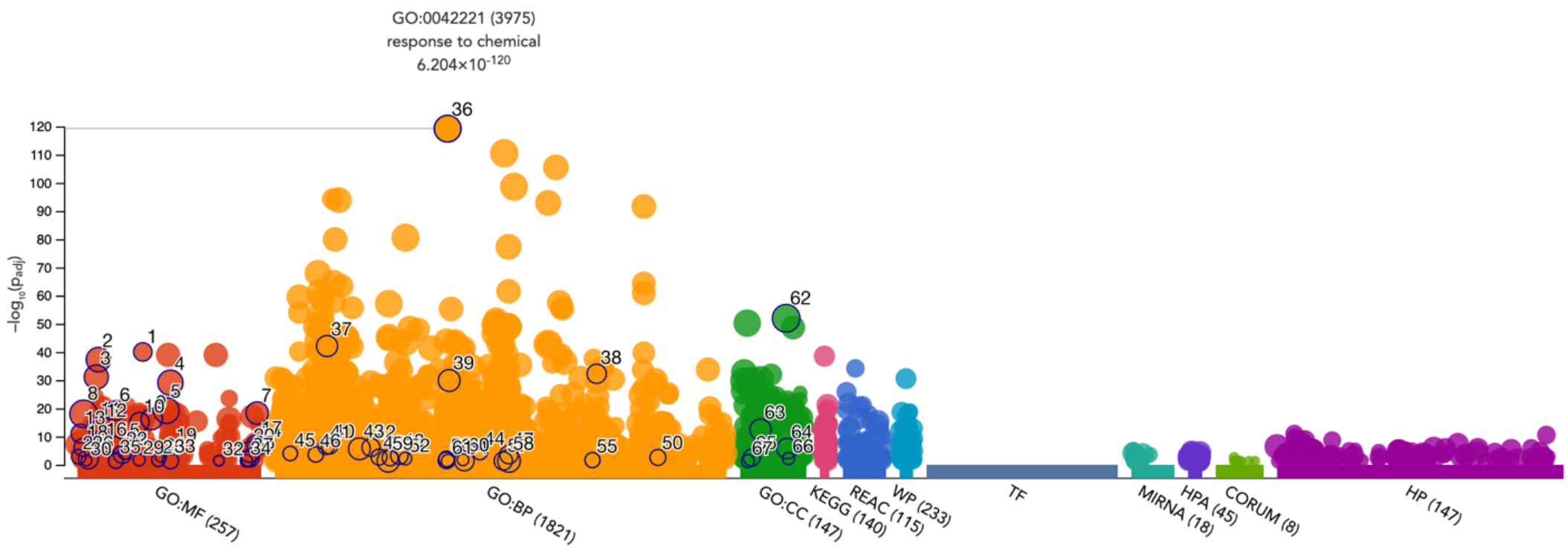


**Figure S1.** **Gene ontology of the 772 unique spaceflight-associated genes.**

# SUPPLEMENTARY TABLES

## SUPPLEMENTARY TABLE S1A: CATALOG OF CLINICAL PHARMACEUTICALS USED IN SPACEFLIGHT

Table S1: **Clinical Pharmaceuticals.**

| Drug Name | Publication | PMID | Condition | Timing | Mission | Crew | Location |
|---|---|---|---|---|---|---|---|
| 2,2-DIPYRIDYL | Cultivation in Space Flight Produces Minimal Alterations in the Susceptibility of Bacillus subtilis Cells to 72 Different Antibiotics and Growth-Inhibiting Compounds | 28821547 | bacterial infection | post-flight | Bacillus subtilis | 1 | ISS |
| 5-FLUOROTIC ACID | Cultivation in Space Flight Produces Minimal Alterations in the Susceptibility of Bacillus subtilis Cells to 72 Different Antibiotics and Growth-Inhibiting Compounds | 28821547 | bacterial infection | post-flight | Bacillus subtilis | 1 | ISS |
| 5-FLUOROURACIL | Cultivation in Space Flight Produces Minimal Alterations in the Susceptibility of Bacillus subtilis Cells to 72 Different Antibiotics and Growth-Inhibiting Compounds | 28821547 | bacterial infection | post-flight | Bacillus subtilis | 1 | ISS |
| 6-MERCAPTOPURINE | Cultivation in Space Flight Produces Minimal Alterations in the Susceptibility of Bacillus subtilis Cells to 72 Different Antibiotics and Growth-Inhibiting Compounds | 28821547 | bacterial infection | post-flight | Bacillus subtilis | 1 | ISS |
| 9-RIBOFURANYZYL-HYPOXANTHIN | Chapter 4 Pharmacology in Space: Pharmacotherapy | 9048135 | increases myofibril potential | in-flight | 166-day MIR mission | 2 | MIR Space Station |
| ACETAMINOPHEN | Medication Use by US Crewmembers on the International Space Station | 26187345 | pain (back, joint, muscle, and headaches) | in-flight | Various missions from 2002-2012 (mix of short and long duration spaceflight) | 24 | ISS |
| ACETAMINOPHEN | Pharmaceutical Use by US Astronauts on Space Shuttle Missions (Source: "Supplying a Pharmacy for NASA Exploration Spaceflight: Challenges and Current Understanding") | 10417009 (31231-676) | pain relief (not entirely clear which type of pain) | in-flight | 79 space shuttle missions | 219 | Space shuttles |
| ACETAMINOPHEN | Factors Affecting Drug Bioavailability in Space | 8083399 | pain relief (not entirely clear which type of pain) | in-flight | First 33 space shuttle missions | A specific number is unclear, but 500 individual doses were administered over 83% of the crewmembers | 33 space shuttles |
| ACETAMINOPHEN | Where Failure is not an Option - Personalized Medicine in Astronauts | 26489089 | pain / fever | in-flight | Not one specific mission, but an accumulation of drugs aboard the ISS (as of 2014) | Unclear; not referenced specifically | ISS |
| ACETAMINOPHEN | Pharmacokinetic Consequences of Spaceflight | 11537657 | pain relief | In-flight | Did not specify | Did not specify | Did not specify |
| ACETAMINOPHEN | Pharmacokinetics of Acetaminophen Administered in Tablets and Capsules Under Long-Term Spaceflight Conditions | | pain relief | in-flight | ISS space expeditions | 10 | ISS |
| ACETAMINOPHEN | Chemical Potency and Degradation Products of Medications Stored Over 550 Earth Days at the International Space Station | 26546565 | pain relief | tested post-flight | Sent via Russian Progress vehicle; returned via SpaceX Dragon Capsule | N/A (drugs were not administered to patients, but tested for efficacy) | ISS / ground |
| ACETYLSALICYLIC ACID | Chemical Potency and Degradation Products of Medications Stored Over 550 Earth Days at the International Space Station | 26546565 | pain relief | tested post-flight | Sent via Russian Progress vehicle; returned via SpaceX Dragon Capsule | N/A (drugs were not administered to patients, but tested for efficacy) | ISS / ground |
| ACETYLSALICYLIC ACID | Pharmaceutical Use by US Astronauts on Space Shuttle Missions (Source: "Supplying a Pharmacy for NASA Exploration Spaceflight: Challenges and Current Understanding") | 10417009 (31231-676) | pain relief (not entirely clear which type of pain) | in-flight | 79 space shuttle missions | 219 | Space shuttles |
| ACETYLSALICYLIC ACID | Factors Affecting Drug Bioavailability in Space | 8083399 | pain relief (not entirely clear which type of pain) | in-flight | First 33 space shuttle missions | A specific number is unclear, but 500 individual doses were administered over 83% of the crewmembers | 33 space shuttles |

*Continued on next page*

Table S1 – *Continued from previous page*

| Drug Name | Publication | PMID | Condition | Timing | Mission | Crew | Location |
|---|---|---|---|---|---|---|---|
| ACETYLSALICYLIC ACID; ACETAMINOPHEN; CAFFEINE | Azithromycin | 26187345 | headaches | in-flight | Various missions from 2002-2012 (mix of short and long duration spaceflight) | 24 | ISS |
| ACETYLSALICYLIC ACID; MAGNESIUM OXIDE | Factors Affecting Drug Bioavailability in Space | 8083399 | pain relief / gastrointestinal discomfort (not entirely clear) | in-flight | First 33 space shuttle missions | A specific number is unclear, but 500 individual doses were administered over 83% of the crewmembers | 33 space shuttles |
| ACETYLSALICYLIC ACID | Where Failure is not an Option - Personalized Medicine in Astronauts | 26489089 | pain / fever / cardiovascular health | in-flight | Not one specific mission, but an accumulation of drugs aboard the ISS (as of 2014) | Unclear; not referenced specifically | ISS |
| ACICLOVIR | Evaluation of Physical and Chemical Changes in Pharmaceuticals Flown on Space Missions | 21479701 | viral infections | in-flight | ISS palyloads | N/A (these drugs were just stored, not administered) | ISS |
| ALENDRONATE | Biphosphonates as a Supplement to Excercise to Protect Bone During Long-Duration Spaceflight | 23334732 | bone protection / osteoporosis | in-flight | Doesn't specify when the mission was; only says that the dates ranged from 4.5 to 6.2 months | 7 | ISS |
| AMIKACIN | Multidrug-resistant Acinetobacter pittii is adapting to and exhibiting potential succession aboard the International Space Station | 36503581 | bacterial infection | post-flight | A. pittii | 1 | ISS |
| AMIKACIN | Cultivation in Space Flight Produces Minimal Alterations in the Susceptibility of Bacillus subtilis Cells to 72 Different Antibiotics and Growth-Inhibiting Compounds | 28821547 | bacterial infection | post-flight | Bacillus subtilis | 1 | ISS |
| AMOXICILLIN | Resilient microorganisms in dust samples of the International Space Station-survival of the adaptation specialists | 27998314 | bacterial infection | post-flight | 8-12 years old dust samples | 1 | Russian ISS Modules |
| AMOXICILLIN | Cultivation in Space Flight Produces Minimal Alterations in the Susceptibility of Bacillus subtilis Cells to 72 Different Antibiotics and Growth-Inhibiting Compounds | 28821547 | bacterial infection | post-flight | Bacillus subtilis | 1 | ISS |
| AMOXICILLIN / CLAVULANATE | Evaluation of Physical and Chemical Changes in Pharmaceuticals Flown on Space Missions | 21479701 | infections | in-flight | ISS/ STS-122 | N/A (these drugs were just stored, not administered) | ISS |
| AMOXICILLIN / CLAVULANIC ACID | Evaluation of Acquired Antibiotic Resistance in Escericihia coli Exposed to Long-Term Low-Shear Stress Modeled Microgravity and Background Antibiotic Exposure | 30647159 | E.coli infection | simulated microgravity | No specific mission (simulated microgravity) | N/A (not crew members, but bacterial strains) | N/A (simulated microgravity) |
| AMPICILLIN | Novel Antibiotic Testing Approaches Reveal Reduced Antibiotic Efficacy Against Shiga Toxin-Producing Escherichia coli O157:H7 Under Simulated Microgravity | 30619237 | bacterial infection | RCSS simulation | | Shiga Toxin-Producing Escherichia coli O157:H7 | RCSS simulation |
| AMPICILLIN | Multi-resistant biofilm-forming pathogens on the International Space Station | 31719234 | bacterial infection | in-flight | 2013-2015 - S. Aureus | 1 | ISS |
| AMPICILLIN | Multi-resistant biofilm-forming pathogens on the International Space Station | 31719234 | bacterial infection | in-flight | 2013-2015 - S. hominis | 1 | ISS |
| AMPICILLIN | Multi-resistant biofilm-forming pathogens on the International Space Station | 31719234 | bacterial infection | in-flight | 2013-2015 - S. epidermidis | 1 | ISS |
| AMPICILLIN | Multi-resistant biofilm-forming pathogens on the International Space Station | 31719234 | bacterial infection | in-flight | 2013-2015 - diverse surfaces | 1 | ISS |
| AMPICILLIN | Multidrug-resistant Acinetobacter pittii is adapting to and exhibiting potential succession aboard the International Space Station | 36503581 | bacterial infection | post-flight | A. pittii | 1 | ISS |
| AMPICILLIN | Resilient microorganisms in dust samples of the International Space Station-survival of the adaptation specialists | 27998314 | bacterial infection | post-flight | 8-12 years old dust samples | 1 | Russian ISS Modules |

*Continued on next page*

Table S1 – *Continued from previous page*

| Drug Name | Publication | PMID | Condition | Timing | Mission | Crew | Location |
|---|---|---|---|---|---|---|---|
| AMPICILLIN | Cultivation in Space Flight Produces Minimal Alterations in the Susceptibility of Bacillus subtilis Cells to 72 Different Antibiotics and Growth-Inhibiting Compounds | 28821547 | bacterial infection | post-flight | Bacillus subtilis | 1 | ISS |
| AMPICILLIN | Evaluation of Acquired Antibiotic Resistance in Escericihia coli Exposed to Long-Term Low-Shear Stress Modeled Microgravity and Background Antibiotic Exposure | 30647159 | E.coli infection | simulated microgravity | No specific mission (simulated microgravity) | N/A (not crew members, but bacterial strains) | N/A (simulated microgravity) |
| AMPICILLIN | Genome Sequence of Escherichia coli Strain LCT-EC52, Which Acquired Changes in Antibiotic Resistance Properties after the Shenzhou-VIII Mission | 24604641 | E.coli associated diseaseses (ex. dysentery, bacteremia, UTIs) | In-flight | Shenzhou-VIII | N/A (not crew members, but bacterial strains) | Shenzhou-VIII (17 days aboard spacecraft) |
| APIXABAN | Venous Thrombosis during Spaceflight | 31893522 | venous thrombosis (blood clots) | In-flight | Did not specify | Did not specify | ISS |
| ARIPIPRAZOLE | Where Failure is not an Option - Personalized Medicine in Astronauts | 26489089 | psychosis | in-flight | Not one specific mission, but an accumulation of drugs aboard the ISS (as of 2014) | Unclear; not referenced specifically | ISS |
| ASCORBIC ACID; N-ACETYL CYSTEINE; L-SELENOMETHIONINE; DIHYDROLIPOIC ACID; VITAMIN E | Dried plum diet protects from bone loss caused by ionizing radiation | 26867002 | microgravity--induced bone loss | radiation exposure | | n/a (marrow cells in vitro) | simulated radiation |
| ATROVASTATIN | Evaluation of Physical and Chemical Changes in Pharmaceuticals Flown on Space Missions | 21479701 | high cholesterol / triglycerides | in-flight | ISS payloads | N/A (these drugs were just stored, not administered) | ISS |
| AZITHROMYCIN | Genome Sequence of Escherichia coli Strain LCT-EC52, Which Acquired Changes in Antibiotic Resistance Properties after the Shenzhou-VIII Mission | 24604641 | E.coli associated diseaseses (ex. dysentery, bacteremia, UTIs) | In-flight | Shenzhou-VIII | N/A (not crew members, but bacterial strains) | Shenzhou-VIII (17 days aboard spacecraft) |
| AZITHROMYCIN | Evaluation of Physical and Chemical Changes in Pharmaceuticals Flown on Space Missions | 21479701 | bacterial infections | in-flight | ISS payloads | N/A (these drugs were just stored, not administered) | ISS |
| AZLOCILLIN | Cultivation in Space Flight Produces Minimal Alterations in the Susceptibility of Bacillus subtilis Cells to 72 Different Antibiotics and Growth-Inhibiting Compounds | 28821547 | bacterial infection | post-flight | Bacillus subtilis | 1 | ISS |
| BACITRACIN | Responses of haloarchaea to simulated microgravity | 21417742 | archea resistance | RCSS simulation | | Haloferax mediterranei | RCSS simulation |
| BENZETHONIUM CHLORIDE | Cultivation in Space Flight Produces Minimal Alterations in the Susceptibility of Bacillus subtilis Cells to 72 Different Antibiotics and Growth-Inhibiting Compounds | 28821547 | bacterial infection | post-flight | Bacillus subtilis | 1 | ISS |
| BENZYLPENICILLIN (PENICILLIN G) | Cultivation in Space Flight Produces Minimal Alterations in the Susceptibility of Bacillus subtilis Cells to 72 Different Antibiotics and Growth-Inhibiting Compounds | 28821547 | bacterial infection | post-flight | Bacillus subtilis | 1 | ISS |
| BIFIDUMBACTERIN | Chapter 4 Pharmacology in Space: Pharmacotherapy | 9048135 | prevents dysbacteriosis | in-flight | 166-day MIR mission | 2 | MIR Space Station |
| BISACODYL | Pharmaceutical Use by US Astronauts on Space Shuttle Missions (Source: "Supplying a Pharmacy for NASA Exploration Spaceflight: Challenges and Current Understanding") | 10417009 (31231-676) | gastrointestinal discomfort | in-flight | 79 space shuttle missions | 219 | Space shuttles |
| BISACODYL | Factors Affecting Drug Bioavailability in Space | 8083399 | gastrointestinal discomfort | in-flight | First 33 space shuttle missions | A specific number is unclear, but 500 individual doses were administered over 83% of the crewmembers | 33 space shuttles |
| BLEOMYCIN | Cultivation in Space Flight Produces Minimal Alterations in the Susceptibility of Bacillus subtilis Cells to 72 Different Antibiotics and Growth-Inhibiting Compounds | 28821547 | bacterial infection | post-flight | Bacillus subtilis | 1 | ISS |

*Continued on next page*

Table S1 – *Continued from previous page*

| Drug Name | Publication | PMID | Condition | Timing | Mission | Crew | Location |
|---|---|---|---|---|---|---|---|
| BROMPHENIRAMINE MALEATE; PHENYLPROPANOLAMINE | Factors Affecting Drug Bioavailability in Space | 8083399 | allergies / congestion | in-flight | First 33 space shuttle missions | A specific number is unclear, but 500 individual doses were administered over 83% of the crewmembers | 33 space shuttles |
| CAFFEINE | Prevalence of Sleep Deficiency and use of hypnotic drugs in astronauts before, during, and after spaceflight: an observational study (NOTE: this is from the supplemental materials section) | 25127232 | irregular sleep / alertness | in-flight | Various space shuttle missions | 72 of 80 shuttle crewmembers | Space shuttles |
| CAFFEINE | Where Failure is not an Option - Personalized Medicine in Astronauts | 26489089 | alertness | in-flight | Not one specific mission, but an accumulation of drugs aboard the ISS (as of 2014) | Unclear; not referenced specifically | ISS |
| CAPREOMYCIN | Cultivation in Space Flight Produces Minimal Alterations in the Susceptibility of Bacillus subtilis Cells to 72 Different Antibiotics and Growth-Inhibiting Compounds | 28821547 | bacterial infection | post-flight | Bacillus subtilis | 1 | ISS |
| CARBENICILLIN | Cultivation in Space Flight Produces Minimal Alterations in the Susceptibility of Bacillus subtilis Cells to 72 Different Antibiotics and Growth-Inhibiting Compounds | 28821547 | bacterial infection | post-flight | Bacillus subtilis | 1 | ISS |
| CEFALEXIN / CEPHALEXIN | Simulated microgravity affects some biological characteristics of Lactobacillus acidophilus | 28013406 | bacterial infection | RCSS simulation | | Lactobacillus acidophilus | RCSS simulation |
| CEFALOTIN / CEFALOTHIN | Immunity in Space: Prokaryote Adaptations and Immune Response in Microgravity | 33540536 | bacterial infection | RCSS simulation | | e.coli | RCSS simulation for 1000 generations |
| CEFALOTIN / CEPHALOTHIN | Evaluation of Acquired Antibiotic Resistance in Escericihia coli Exposed to Long-Term Low-Shear Stress Modeled Microgravity and Background Antibiotic Exposure | 30647159 | E.coli resistance | simulated microgravity | No specific mission (simulated microgravity) | N/A (not crew members, but bacterial strains) | N/A (simulated microgravity) |
| CEFAZOLIN | Detection of antimicrobial resistance genes associated with the International Space Station environmental surfaces | 29339831 | bacterial infection | post-flight | Fifty-seven Biosafety Level-2 (BSL-2) microorganisms | 1 | ISS |
| CEFAZOLIN | Cultivation in Space Flight Produces Minimal Alterations in the Susceptibility of Bacillus subtilis Cells to 72 Different Antibiotics and Growth-Inhibiting Compounds | 28821547 | bacterial infection | post-flight | Bacillus subtilis | 1 | ISS |
| CEFAZOLIN | Evaluation of Acquired Antibiotic Resistance in Escericihia coli Exposed to Long-Term Low-Shear Stress Modeled Microgravity and Background Antibiotic Exposure | 30647159 | E.coli infection | simulated microgravity | No specific mission (simulated microgravity) | N/A (not crew members, but bacterial strains) | N/A (simulated microgravity) |
| CEFAZOLIN | Genome Sequence of Escherichia coli Strain LCT-EC52, Which Acquired Changes in Antibiotic Resistance Properties after the Shenzhou-VIII Mission | 24604641 | E.coli associated diseaseses (ex. dysentery, bacteremia, UTIs) | In-flight | Shenzhou-VIII | N/A (not crew members, but bacterial strains) | Shenzhou-VIII (17 days aboard spacecraft) |
| CEFEPIME | Multidrug-resistant Acinetobacter pittii is adapting to and exhibiting potential succession aboard the International Space Station | 36503581 | bacterial infection | post-flight | A. pittii | 1 | ISS |
| CEFEPIME | Evaluation of Acquired Antibiotic Resistance in Escericihia coli Exposed to Long-Term Low-Shear Stress Modeled Microgravity and Background Antibiotic Exposure | 30647159 | E.coli infection | simulated microgravity | No specific mission (simulated microgravity) | N/A (not crew members, but bacterial strains) | N/A (simulated microgravity) |
| CEFOTAXIME | Multidrug-resistant Acinetobacter pittii is adapting to and exhibiting potential succession aboard the International Space Station | 36503581 | bacterial infection | post-flight | A. pittii | 1 | ISS |
| CEFOTAXIME | Resilient microorganisms in dust samples of the International Space Station-survival of the adaptation specialists | 27998314 | bacterial infection | post-flight | 8-12 years old dust samples | 1 | Russian ISS Modules |
| CEFOXITIN | Immunity in Space: Prokaryote Adaptations and Immune Response in Microgravity | 33540536 | bacterial infection | RCSS simulation | | e.coli | RCSS simulation for 1000 generations |

*Continued on next page*

Table S1 – *Continued from previous page*

| Drug Name | Publication | PMID | Condition | Timing | Mission | Crew | Location | |
|---|---|---|---|---|---|---|---|---|
| CEFOXITIN | Detection of antimicrobial resistance genes associated with the International Space Station environmental surfaces | 29339831 | bacterial infection | post-flight | Fifty-seven Biosafety Level-2 (BSL-2) microorganisms | 1 | ISS | |
| CEFOXITIN | Evaluation of Acquired Antibiotic Resistance in Escericihia coli Exposed to Long-Term Low-Shear Stress Modeled Microgravity and Background Antibiotic Exposure | 30647159 | E.coli resistance | simulated microgravity | No specific mission (simulated microgravity) | N/A (not crew members, but bacterial strains) | N/A (simulated microgravity) | |
| CEFPODOXIME | Evaluation of Acquired Antibiotic Resistance in Escericihia coli Exposed to Long-Term Low-Shear Stress Modeled Microgravity and Background Antibiotic Exposure | 30647159 | E.coli infection | simulated microgravity | No specific mission (simulated microgravity) | N/A (not crew members, but bacterial strains) | N/A (simulated microgravity) | |
| CEFRADROXIL | Evaluation of Physical and Chemical Changes in Pharmaceuticals Flown on Space Missions | 21479701 | bacterial infections | in-flight | ISS payloads | N/A (these drugs were just stored, not administered) | ISS | |
| CEFTAZIDIME | Multidrug-resistant Acinetobacter pittii is adapting to and exhibiting potential succession aboard the International Space Station | 36503581 | bacterial infection | post-flight | A. pittii | 1 | ISS | |
| CEFTAZIDIME | Evaluation of Acquired Antibiotic Resistance in Escericihia coli Exposed to Long-Term Low-Shear Stress Modeled Microgravity and Background Antibiotic Exposure | 30647159 | E.coli infection | simulated microgravity | No specific mission (simulated microgravity) | N/A (not crew members, but bacterial strains) | N/A (simulated microgravity) | |
| CEFTAZIDIME | Genome Sequence of Escherichia coli Strain LCT-EC52, Which Acquired Changes in Antibiotic Resistance Properties after the Shenzhou-VIII Mission | 24604641 | E.coli associated diseaseses (ex. dysentery, bacteremia, UTIs) | In-flight | Shenzhou-VIII | N/A (not crew members, but bacterial strains) | Shenzhou-VIII (17 days aboard spacecraft) | |
| CEFTRIAXONE | Multidrug-resistant Acinetobacter pittii is adapting to and exhibiting potential succession aboard the International Space Station | 36503581 | bacterial infection | post-flight | A. pittii | 1 | ISS | |
| CEFTRIAXONE | Resilient microorganisms in dust samples of the International Space Station-survival of the adaptation specialists | 27998314 | bacterial infection | post-flight | 8-12 years old dust samples | 1 | Russian ISS Modules | |
| CEFTRIAXONE | Cultivation in Space Flight Produces Minimal Alterations in the Susceptibility of Bacillus subtilis Cells to 72 Different Antibiotics and Growth-Inhibiting Compounds | 28821547 | bacterial infection | post-flight | Bacillus subtilis | 1 | ISS | |
| CEFTRIAXONE | Evaluation of Acquired Antibiotic Resistance in Escericihia coli Exposed to Long-Term Low-Shear Stress Modeled Microgravity and Background Antibiotic Exposure | 30647159 | E.coli infection | simulated microgravity | No specific mission (simulated microgravity) | N/A (not crew members, but bacterial strains) | N/A (simulated microgravity) | |
| CEFTRIAXONE | Genome Sequence of Escherichia coli Strain LCT-EC52, Which Acquired Changes in Antibiotic Resistance Properties after the Shenzhou-VIII Mission | 24604641 | E.coli associated diseaseses (ex. dysentery, bacteremia, UTIs) | In-flight | Shenzhou-VIII | N/A (not crew members, but bacterial strains) | Shenzhou-VIII (17 days aboard spacecraft) | |
| CEFUROXIME | Immunity in Space: Prokaryote Adaptations and Immune Response in Microgravity | 33540536 | bacterial infection | RCSS simulation | | e.coli | RCSS simulation for 1000 generations | |
| CEFUROXIME | Immunity in Space: Prokaryote Adaptations and Immune Response in Microgravity | 33540536 | bacterial infection | RCSS simulation | | e.coli | RCSS simulation for 1000 generations | |
| CEFUROXIME | Cultivation in Space Flight Produces Minimal Alterations in the Susceptibility of Bacillus subtilis Cells to 72 Different Antibiotics and Growth-Inhibiting Compounds | 28821547 | bacterial infection | post-flight | Bacillus subtilis | 1 | ISS | |
| CEFUROXIME | Evaluation of Acquired Antibiotic Resistance in Escericihia coli Exposed to Long-Term Low-Shear Stress Modeled Microgravity and Background Antibiotic Exposure | 30647159 | E.coli resistance | simulated microgravity | No specific mission (simulated microgravity) | N/A (not crew members, but bacterial strains) | N/A (simulated microgravity) | |

*Continued on next page*

Table S1 – *Continued from previous page*

| Drug Name | Publication | PMID | Condition | Timing | Mission | Crew | Location |
|---|---|---|---|---|---|---|---|
| CEFUROXIME ACETIL | Evaluation of Acquired Antibiotic Resistance in Escericihia coli Exposed to Long-Term Low-Shear Stress Modeled Microgravity and Background Antibiotic Exposure | 30647159 | E.coli resistance | simulated microgravity | No specific mission (simulated microgravity) | N/A (not crew members, but bacterial strains) | N/A (simulated microgravity) |
| CEFUROZIME AXETIL | Immunity in Space: Prokaryote Adaptations and Immune Response in Microgravity | 33540536 | bacterial infection | RCSS simulation | | e.coli | RCSS simulation for 1000 generations |
| CEPHALOTHIN | Cultivation in Space Flight Produces Minimal Alterations in the Susceptibility of Bacillus subtilis Cells to 72 Different Antibiotics and Growth-Inhibiting Compounds | 28821547 | bacterial infection | post-flight | Bacillus subtilis | 1 | ISS |
| CESIUM CHLORIDE | Cultivation in Space Flight Produces Minimal Alterations in the Susceptibility of Bacillus subtilis Cells to 72 Different Antibiotics and Growth-Inhibiting Compounds | 28821547 | bacterial infection | post-flight | Bacillus subtilis | 1 | ISS |
| CETIRIZINE | Where Failure is not an Option - Personalized Medicine in Astronauts | 26489089 | vomiting / allergic rhinitis | in-flight | Not one specific mission, but an accumulation of drugs aboard the ISS (as of 2014) | Unclear; not referenced specifically | ISS |
| CHLORAMPHENICOL | Immunity in Space: Prokaryote Adaptations and Immune Response in Microgravity | 33540536 | bacterial infection | RCSS simulation | | e.coli | RCSS simulation for 1000 generations |
| CHLORAMPHENICOL | Multi-resistant biofilm-forming pathogens on the International Space Station | 31719234 | bacterial infection | in-flight | Salyut 7 | 1 | Salyut 7 |
| CHLORAMPHENICOL | Multi-resistant biofilm-forming pathogens on the International Space Station | 31719234 | bacterial infection | in-flight | 2013-2015 - E. faecalis | 1 | ISS |
| CHLORAMPHENICOL | Cultivation in Space Flight Produces Minimal Alterations in the Susceptibility of Bacillus subtilis Cells to 72 Different Antibiotics and Growth-Inhibiting Compounds | 28821547 | bacterial infection | post-flight | Bacillus subtilis | 1 | ISS |
| CHLORAMPHENICOL | Study of minimal inhibitory concentration of antibiotics on bacteria cultivated in vitro in space (Cytos 2 experiment) | 3899095 | Weak increase in Staphylococcus aureus resistance to antibiotics | in-flight | Cytos 2 | N/A (Not crewmate but bacteria | Not named (French-Soviet manned flight in July 1982) |
| CHLORTETRACYCLINE | Cultivation in Space Flight Produces Minimal Alterations in the Susceptibility of Bacillus subtilis Cells to 72 Different Antibiotics and Growth-Inhibiting Compounds | 28821547 | bacterial infection | post-flight | Bacillus subtilis | 1 | ISS |
| CHLORZOXAZONE | Factors Affecting Drug Bioavailability in Space | 8083399 | muscle pain | in-flight | First 33 space shuttle missions | A specific number is unclear, but 500 individual doses were administered over 83% of the crewmembers | 33 space shuttles |
| CINNARIZINE; DIMENHYDRINATE | Antihistamines for motion sickness | 36250781 | decreased risk of developing motion sickness symptoms | rotating chair model | | 658 | ground-study seven countries |
| CIPROFLOXACIN | Resilient microorganisms in dust samples of the International Space Station-survival of the adaptation specialists | 27998314 | bacterial infection | post-flight | 8-12 years old dust samples | 1 | Russian ISS Modules |
| CIPROFLOXACIN | Detection of antimicrobial resistance genes associated with the International Space Station environmental surfaces | 29339831 | bacterial infection | post-flight | Fifty-seven Biosafety Level-2 (BSL-2) microorganisms | 1 | ISS |
| CIPROFLOXACIN | Evaluation of Acquired Antibiotic Resistance in Escericihia coli Exposed to Long-Term Low-Shear Stress Modeled Microgravity and Background Antibiotic Exposure | 30647159 | E.coli infection | simulated microgravity | No specific mission (simulated microgravity) | N/A (not crew members, but bacterial strains) | N/A (simulated microgravity) |
| CIPROFLOXACIN | Effect of Simulated Microgravity on the Disposition and Tissue Penetration of Ciprofloxacin in Healthy Volunteers | 15951472 | anti-infection (including anti UTIs) | simulated microgravity (using the antiorthostatic bed rest model) | No specific mission (simulated microgravity) | 6 | On ground (antiorthostatic bed rest) |

*Continued on next page*

Table S1 – *Continued from previous page*

| Drug Name | Publication | PMID | Condition | Timing | Mission | Crew | Location |
|---|---|---|---|---|---|---|---|
| CIPROFLOXACIN | Evaluation of Physical and Chemical Changes in Pharmaceuticals Flown on Space Missions | 21479701 | infections | in-flight | ISS / STS-122 | N/A (these drugs were just stored, not administered) | ISS |
| CIPROFLOXACIN | Evaluation of Physical and Chemical Changes in Pharmaceuticals Flown on Space Missions | 21479701 | conjunctivitis | in-flight | ISS / STS-122 | N/A (these drugs were just stored, not administered) | ISS |
| CLARITHROMYCIN | Resilient microorganisms in dust samples of the International Space Station-survival of the adaptation specialists | 27998314 | bacterial infection | post-flight | 8-12 years old dust samples | 1 | Russian ISS Modules |
| CLINDAMYCIN | Multi-resistant biofilm-forming pathogens on the International Space Station | 31719234 | bacterial infection | in-flight | filter samples | 1 | ISS |
| CLINDAMYCIN | Multi-resistant biofilm-forming pathogens on the International Space Station | 31719234 | bacterial infection | in-flight | 2013-2015 - S. Aureus | 1 | ISS |
| CLINDAMYCIN | Multi-resistant biofilm-forming pathogens on the International Space Station | 31719234 | bacterial infection | in-flight | 2013-2015 - S. hominis | 1 | ISS |
| CLINDAMYCIN | Multi-resistant biofilm-forming pathogens on the International Space Station | 31719234 | bacterial infection | in-flight | 2013-2015 - E. faecalis | 1 | ISS |
| CLINDAMYCIN | Resilient microorganisms in dust samples of the International Space Station-survival of the adaptation specialists | 27998314 | bacterial infection | post-flight | 8-12 years old dust samples | 1 | Russian ISS Modules |
| CLOTRIMAZOLE | Evaluation of Physical and Chemical Changes in Pharmaceuticals Flown on Space Missions | 21479701 | fungal infections | in-flight | ISS payloads | N/A (these drugs were just stored, not administered) | ISS |
| CLOXACILLIN | Cultivation in Space Flight Produces Minimal Alterations in the Susceptibility of Bacillus subtilis Cells to 72 Different Antibiotics and Growth-Inhibiting Compounds | 28821547 | bacterial infection | post-flight | Bacillus subtilis | 1 | ISS |
| COBALT CHLORIDE | Cultivation in Space Flight Produces Minimal Alterations in the Susceptibility of Bacillus subtilis Cells to 72 Different Antibiotics and Growth-Inhibiting Compounds | 28821547 | bacterial infection | post-flight | Bacillus subtilis | 1 | ISS |
| COBOLAMINE | Evaluation of Physical and Chemical Changes in Pharmaceuticals Flown on Space Missions | 21479701 | maintenance of vitmain B12 | in-flight | ISS payloads | N/A (these drugs were just stored, not administered) | ISS |
| CODEINE | Factors Affecting Drug Bioavailability in Space | 8083399 | pain relief (not entirely clear which type of pain) | in-flight | First 33 space shuttle missions | A specific number is unclear, but 500 individual doses were administered over 83% of the crewmembers | 33 space shuttles |
| COLISTIN | Chapter 4 Pharmacology in Space: Pharmacotherapy | 9048135 | urinary tract infection ; Pseudomonas aerogunisae | post-flight | Apollo 13 | 1 | Moon Landing |
| COLISTIN | Multi-resistant biofilm-forming pathogens on the International Space Station | 31719234 | bacterial infection | in-flight | Salyut 7 | 1 | Salyut 7 |
| COLISTIN | Resilient microorganisms in dust samples of the International Space Station-survival of the adaptation specialists | 27998314 | bacterial infection | post-flight | 8-12 years old dust samples | 1 | Russian ISS Modules |
| COLISTIN | Cultivation in Space Flight Produces Minimal Alterations in the Susceptibility of Bacillus subtilis Cells to 72 Different Antibiotics and Growth-Inhibiting Compounds | 28821547 | bacterial infection | post-flight | Bacillus subtilis | 1 | ISS |
| COLISTIN | Study of minimal inhibitory concentration of antibiotics on bacteria cultivated in vitro in space (Cytos 2 experiment) | 3899095 | Strong increase in Ecoli resistance to antibiotic | in-flight | Cytos 2 | N/A (Not crewmate but bacteria | Not named (French-Soviet manned flight in July 1982) |
| CUPRIC CHLORIDE | Cultivation in Space Flight Produces Minimal Alterations in the Susceptibility of Bacillus subtilis Cells to 72 Different Antibiotics and Growth-Inhibiting Compounds | 28821547 | bacterial infection | post-flight | Bacillus subtilis | 1 | ISS |
| CYCLIZINE | Chapter 4 Pharmacology in Space: Pharmacotherapy | 9048135 | space motion sickness | in-flight | Apollo 7-10 | 15 | Orbital Flights + Moon Landing |

*Continued on next page*

Table S1 – *Continued from previous page*

| Drug Name | Publication | PMID | Condition | Timing | Mission | Crew | Location |
|---|---|---|---|---|---|---|---|
| CYTOSINE-1-*ta*-D-ARABINOFURAN-OSIDE | Cultivation in Space Flight Produces Minimal Alterations in the Susceptibility of Bacillus subtilis Cells to 72 Different Antibiotics and Growth-Inhibiting Compounds | 28821547 | bacterial infection | post-flight | Bacillus subtilis | 1 | ISS |
| DEMECLOCYCLINE | Cultivation in Space Flight Produces Minimal Alterations in the Susceptibility of Bacillus subtilis Cells to 72 Different Antibiotics and Growth-Inhibiting Compounds | 28821547 | bacterial infection | post-flight | Bacillus subtilis | 1 | ISS |
| DEQUALINIUM CHLORIDE | Cultivation in Space Flight Produces Minimal Alterations in the Susceptibility of Bacillus subtilis Cells to 72 Different Antibiotics and Growth-Inhibiting Compounds | 28821547 | bacterial infection | post-flight | Bacillus subtilis | 1 | ISS |
| DEXAMETHASONE | Dexamethasone mimicks the antimotion sickness effects of amphetamine and scopolamine | 11538662 | space motion sickness | rotating chair model | | 19 | ground--study |
| DEXTROAMPHETAMINE | Evaluation of Physical and Chemical Changes in Pharmaceuticals Flown on Space Missions | 21479701 | ADHD | in-flight | ISS payloads | N/A (these drugs were just stored, not administered) | ISS |
| DEXTROAMPHETAMINE SULFATE | Factors Affecting Drug Bioavailability in Space | 8083399 | not entirely clear; it is associated with ADHD | in-flight | First 33 space shuttle missions | A specific number is unclear, but 500 individual doses were administered over 83% of the crewmembers | 33 space shuttles |
| DIAZEPAM | Factors Affecting Drug Bioavailability in Space | 8083399 | muscle spasms / seizures | in-flight | First 33 space shuttle missions | A specific number is unclear, but 500 individual doses were administered over 83% of the crewmembers | 33 space shuttles |
| DIAZEPAM | Where Failure is not an Option - Personalized Medicine in Astronauts | 26489089 | sleep irregularities | in-flight | Not one specific mission, but an accumulation of drugs aboard the ISS (as of 2014) | Unclear; not referenced specifically | ISS |
| DIHYDROLIPOIC ACID | Dried plum diet protects from bone loss caused by ionizing radiation | 26867002 | microgravity--induced bone loss | radiation exposure | | n/a (marrow cells in vitro) | simulated radiation |
| DIPHENHYDRAMINE | Where Failure is not an Option - Personalized Medicine in Astronauts | 26489089 | vomiting / allergic rhinitis | in-flight | Not one specific mission, but an accumulation of drugs aboard the ISS (as of 2014) | Unclear; not referenced specifically | ISS |
| DL-SERINE HYDROXAMATE | Cultivation in Space Flight Produces Minimal Alterations in the Susceptibility of Bacillus subtilis Cells to 72 Different Antibiotics and Growth-Inhibiting Compounds | 28821547 | bacterial infection | post-flight | Bacillus subtilis | 1 | ISS |
| DOCUSATE | Factors Affecting Drug Bioavailability in Space | 8083399 | gastrointestinal discomfort | in-flight | First 33 space shuttle missions | A specific number is unclear, but 500 individual doses were administered over 83% of the crewmembers | 33 space shuttles |
| DODECYLTRIMETHYLAMMONIUM BROMIDE | Cultivation in Space Flight Produces Minimal Alterations in the Susceptibility of Bacillus subtilis Cells to 72 Different Antibiotics and Growth-Inhibiting Compounds | 28821547 | bacterial infection | post-flight | Bacillus subtilis | 1 | ISS |
| DOXYCYCLINE | Multi-resistant biofilm-forming pathogens on the International Space Station | 31719234 | bacterial infection | in-flight | 2013-2015 - S. hominis | 1 | ISS |
| DOXYCYCLINE | Multi-resistant biofilm-forming pathogens on the International Space Station | 31719234 | bacterial infection | in-flight | 2013-2015 - S. epidermidis | 1 | ISS |
| DOXYCYCLINE | Multi-resistant biofilm-forming pathogens on the International Space Station | 31719234 | bacterial infection | in-flight | 2013-2015 - E. faecalis | 1 | ISS |
| DOXYCYCLINE | Resilient microorganisms in dust samples of the International Space Station-survival of the adaptation specialists | 27998314 | bacterial infection | post-flight | 8-12 years old dust samples | 1 | Russian ISS Modules |
| DOXYCYCLINE | Cultivation in Space Flight Produces Minimal Alterations in the Susceptibility of Bacillus subtilis Cells to 72 Different Antibiotics and Growth-Inhibiting Compounds | 28821547 | bacterial infection | post-flight | Bacillus subtilis | 1 | ISS |

*Continued on next page*

Table S1 – *Continued from previous page*

| Drug Name | Publication | PMID | Condition | Timing | Mission | Crew | Location |
|---|---|---|---|---|---|---|---|
| DRIED PLUM | Dried plum diet protects from bone loss caused by ionizing radiation | 26867002 | microgravity--induced bone loss | radiation exposure | | n/a (marrow cells in vitro) | simulated radiation |
| ENOXACIN | Cultivation in Space Flight Produces Minimal Alterations in the Susceptibility of Bacillus subtilis Cells to 72 Different Antibiotics and Growth-Inhibiting Compounds | 28821547 | bacterial infection | post-flight | Bacillus subtilis | 1 | ISS |
| ENOXAPARIN | Venous Thrombosis during Spaceflight | 31893522 | venous thrombosis (blood clots) | In-flight | Did not specify | Did not specify | ISS |
| EPINEPHRINE | Evaluation of Physical and Chemical Changes in Pharmaceuticals Flown on Space Missions | 21479701 | allergic reactions | in-flight | ISS payloads | N/A (these drugs were just stored, not administered) | ISS |
| ERYTHROMYCIN | Responses of haloarchaea to simulated microgravity | 21417742 | archea resistance | RCSS simulation | | Haloferax mediterranei | RCSS simulation |
| ERYTHROMYCIN | Multi-resistant biofilm-forming pathogens on the International Space Station | 31719234 | bacterial infection | in-flight | Salyut 7 | 1 | Salyut 7 |
| ERYTHROMYCIN | Multi-resistant biofilm-forming pathogens on the International Space Station | 31719234 | bacterial infection | in-flight | Permanent Multipurpose Module - S. Aureus | 1 | ISS |
| ERYTHROMYCIN | Multi-resistant biofilm-forming pathogens on the International Space Station | 31719234 | bacterial infection | in-flight | 2013-2015 - S. Aureus | 1 | ISS |
| ERYTHROMYCIN | Multi-resistant biofilm-forming pathogens on the International Space Station | 31719234 | bacterial infection | in-flight | 2013-2015 - S. hominis | 1 | ISS |
| ERYTHROMYCIN | Multi-resistant biofilm-forming pathogens on the International Space Station | 31719234 | bacterial infection | in-flight | 2013-2015 - S. epidermidis | 1 | ISS |
| ERYTHROMYCIN | Multi-resistant biofilm-forming pathogens on the International Space Station | 31719234 | bacterial infection | in-flight | 2013-2015 - diverse surfaces | 1 | ISS |
| ERYTHROMYCIN | Detection of antimicrobial resistance genes associated with the International Space Station environmental surfaces | 29339831 | bacterial infection | post-flight | Fifty-seven Biosafety Level-2 (BSL-2) microorganisms | 1 | ISS |
| ERYTHROMYCIN | Cultivation in Space Flight Produces Minimal Alterations in the Susceptibility of Bacillus subtilis Cells to 72 Different Antibiotics and Growth-Inhibiting Compounds | 28821547 | bacterial infection | post-flight | Bacillus subtilis | 1 | ISS |
| ERYTHROMYCIN | Study of minimal inhibitory concentration of antibiotics on bacteria cultivated in vitro in space (Cytos 2 experiment) | 3899095 | Weak increase in Staphylococcus aureus resistance to antibiotics | in-flight | Cytos 2 | N/A (Not crewmate but bacteria | Not named (French-Soviet manned flight in July 1982) |
| ESZOPICLONE | Prevalence of Sleep Deficiency and use of hypnotic drugs in astronauts before, during, and after spaceflight: an observational study | 25127232 | irregular sleep | in-flight | Various space shuttle missions | 61 of the 78 crewmembers | Space shuttles |
| FLUCANAZOLE | A case of persistent skin rash and rhinitis with immune system dysregulation onboard the International Space Station | 27036643 | fungal infections from rash | in-flight | ISS Expedition 16-31/32 (Not exactly sure which one as NASA lists all these expedition as associated with study). Paper mentions Soyuz (not exactly sure which specific Soyuz mission). Mentions a 191 flight day mission | 1 | ISS/Soyuz |
| FLUCANOZOLE | Evaluation of Physical and Chemical Changes in Pharmaceuticals Flown on Space Missions | 21479701 | yeast / fungal infections | in-flight | ISS / STS-122 | N/A (these drugs were just stored, not administered) | ISS |
| FLURAZEPAM | Pharmaceutical Use by US Astronauts on Space Shuttle Missions (Source: "Supplying a Pharmacy for NASA Exploration Spaceflight: Challenges and Current Understanding") | 10417009 (31231-676) | insomnia | in-flight | 79 space shuttle missions | 219 | Space shuttles |
| FLURAZEPAM HYDROCHLORIDE | Factors Affecting Drug Bioavailability in Space | 8083399 | insomnia | in-flight | First 33 space shuttle missions | A specific number is unclear, but 500 individual doses were administered over 83% of the crewmembers | 33 space shuttles |

*Continued on next page*

Table S1 – *Continued from previous page*

| Drug Name | Publication | PMID | Condition | Timing | Mission | Crew | Location |
|---|---|---|---|---|---|---|---|
| FUROSEMIDE | Evaluation of Physical and Chemical Changes in Pharmaceuticals Flown on Space Missions | 21479701 | fluid retention / swelling | in-flight | ISS payloads | N/A (these drugs were just stored, not administered) | ISS |
| GENETICIN | Cultivation in Space Flight Produces Minimal Alterations in the Susceptibility of Bacillus subtilis Cells to 72 Different Antibiotics and Growth-Inhibiting Compounds | 28821547 | bacterial infection | post-flight | Bacillus subtilis | 1 | ISS |
| GENTAMICIN | Multi-resistant biofilm-forming pathogens on the International Space Station | 31719234 | bacterial infection | in-flight | 2013-2015 - E. faecalis | 1 | ISS |
| GENTAMICIN | Resilient microorganisms in dust samples of the International Space Station-survival of the adaptation specialists | 27998314 | bacterial infection | post-flight | 8-12 years old dust samples | 1 | Russian ISS Modules |
| GENTAMICIN | Detection of antimicrobial resistance genes associated with the International Space Station environmental surfaces | 29339831 | bacterial infection | post-flight | Fifty-seven Biosafety Level-2 (BSL-2) microorganisms | 1 | ISS |
| GENTAMICIN | Cultivation in Space Flight Produces Minimal Alterations in the Susceptibility of Bacillus subtilis Cells to 72 Different Antibiotics and Growth-Inhibiting Compounds | 28821547 | bacterial infection | post-flight | Bacillus subtilis | 1 | ISS |
| GENTAMICIN SULFATE | Phenotypic Changes Exhibited by E. coli Cultured in Space | 28894439 | bacterial infection | in-flight | Cygnus spacecraft | 44 (e. coli samples) | ISS |
| GENTAMICIN SULFATE | Simulated microgravity affects some biological characteristics of Lactobacillus acidophilus | 28013406 | bacterial infection | RCSS simulation | | Lactobacillus acidophilus | RCSS simulation |
| GENTAMICIN SULFATE | Novel Antibiotic Testing Approaches Reveal Reduced Antibiotic Efficacy Against Shiga Toxin-Producing Escherichia coli O157:H7 Under Simulated Microgravity | 30619237 | bacterial infection | RCSS simulation | | Shiga Toxin-Producing Escherichia coli O157:H7 | RCSS simulation |
| GENTAMICIN SULFATE | Spaceflight Modifies Escherichia coli Gene Expression in Response to Antibiotic Exposure and Reveals Role of Oxidative Stress Response | 29615983 | bacterial infection | in-flight | | e.coli | ISS |
| GENTAMICIN SULFATE | EcAMSat spaceflight measurements of the role of $\sigma$s in antibiotic resistance of stationary phase Escherichia coli in microgravity | 31987476 | bacterial infection | in-flight | Orbital ATK OA-8 resupply | e.coli | low-earth orbit |
| GENTAMICIN | Evaluation of Acquired Antibiotic Resistance in Escericihia coli Exposed to Long-Term Low-Shear Stress Modeled Microgravity and Background Antibiotic Exposure | 30647159 | E.coli infection | simulated microgravity | No specific mission (simulated microgravity) | N/A (not crew members, but bacterial strains) | N/A (simulated microgravity) |
| GLYCINE | Cultivation in Space Flight Produces Minimal Alterations in the Susceptibility of Bacillus subtilis Cells to 72 Different Antibiotics and Growth-Inhibiting Compounds | 28821547 | bacterial infection | post-flight | Bacillus subtilis | 1 | ISS |
| HYDROCODONE | Where Failure is not an Option - Personalized Medicine in Astronauts | 26489089 | pain | in-flight | Not one specific mission, but an accumulation of drugs aboard the ISS (as of 2014) | Unclear; not referenced specifically | ISS |
| HYDROCORTISONE | A case of persistent skin rash and rhinitis with immune system dysregulation onboard the International Space Station | 27036643 | Skin rash, dermatitis | in-flight | ISS Expedition 16-31/32 (Not exactly sure which one as NASA lists all these expedition as associated with study). Paper mentions Soyuz (not exactly sure which specific Soyuz mission). Mentions a 191 flight day mission | 1 | ISS/Soyuz |
| HYDROGEN PEROXIDE | Exposure of Mycobacterium marinum to low-shear modeled microgravity: effect on growth, the transcriptome and survival under stress | 28725743 | bacterial infection | RCSS simulation | | Mycobacterium marinum | RCSS simulation |
| IBUPROFEM | Chemical Potency and Degradation Products of Medications Stored Over 550 Earth Days at the International Space Station | 26546565 | pain relief | tested post-flight | Sent via Russian Progress vehicle; returned via SpaceX Dragon Capsule | N/A (drugs were not administered to patients, but tested for efficacy) | ISS / ground |

*Continued on next page*

Table S1 – *Continued from previous page*

| Drug Name | Publication | PMID | Condition | Timing | Mission | Crew | Location |
|---|---|---|---|---|---|---|---|
| IBUPROFEN | Dried plum diet protects from bone loss caused by ionizing radiation | 26867002 | microgravity--induced bone loss | radiation exposure | | n/a (marrow cells in vitro) | simulated radiation |
| IBUPROFEN | Medication Use by US Crewmembers on the International Space Station | 26187345 | pain (primarily back, joint and muscle pain; often attributed to the advanced resistive exercise device/ARED) | in-flight | Various missions from 2002-2012 (mix of short and long duration spaceflight) | 24 | ISS |
| IBUPROFEN | Factors Affecting Drug Bioavailability in Space | 8083399 | pain relief | in-flight | First 33 space shuttle missions | A specific number is unclear, but 500 individual doses were administered over 83% of the crewmembers | 33 space shuttles |
| IBUPROFEN | Where Failure is not an Option - Personalized Medicine in Astronauts | 26489089 | pain / fever | in-flight | Not one specific mission, but an accumulation of drugs aboard the ISS (as of 2014) | Unclear; not referenced specifically | ISS |
| IBUPROFEN | Evaluation of Physical and Chemical Changes in Pharmaceuticals Flown on Space Missions | 21479701 | pain / fever | in-flight | ISS payloads | N/A (these drugs were just stored, not administered) | ISS |
| IMIPENEM / CILASTATIN | Evaluation of Physical and Chemical Changes in Pharmaceuticals Flown on Space Missions | 21479701 | bacterial infections | in-flight | ISS / STS-117 | N/A (these drugs were just stored, not administered) | ISS |
| INFLUENZA VIRUS, BETA-PROPIOLACTONE, SODIUM TAURODEOXYCHOLATE, SODIUM CHLORIDE, MONOBASIC SODIUM PHOSPHATE, MONOBASIC POTASSIUM PHOSPHATE-, POTASSIUM CHLORIDE, CALCIUM CHLORIDE, OVALBUMIN, SUCROSE, NEOMYCIN SULFATE, POLYMYXIN, HYDROCORTISONE | The NASA Twins Study: A Multidimensional Analysis of a Year-Long Human Spaceflight | 30975860 | influenza | in-flight | Twin Study | 1 | ISS |
| KANAMYCIN | Multi-resistant biofilm-forming pathogens on the International Space Station | 31719234 | bacterial infection | in-flight | Salyut 7 | 1 | Salyut 7 |
| KANAMYCIN | Multi-resistant biofilm-forming pathogens on the International Space Station | 31719234 | bacterial infection | in-flight | 2013-2015 - S. Aureus | 1 | ISS |
| KANAMYCIN | Multi-resistant biofilm-forming pathogens on the International Space Station | 31719234 | bacterial infection | in-flight | 2013-2015 - S. hominis | 1 | ISS |
| KANAMYCIN | Multi-resistant biofilm-forming pathogens on the International Space Station | 31719234 | bacterial infection | in-flight | 2013-2015 - E. faecalis | 1 | ISS |
| KANAMYCIN | Cultivation in Space Flight Produces Minimal Alterations in the Susceptibility of Bacillus subtilis Cells to 72 Different Antibiotics and Growth-Inhibiting Compounds | 28821547 | bacterial infection | post-flight | Bacillus subtilis | 1 | ISS |
| KANAMYCIN | Study of minimal inhibitory concentration of antibiotics on bacteria cultivated in vitro in space (Cytos 2 experiment) | 3899095 | Strong increase in Ecoli resistance to antibiotic | in-flight | Cytos 2 | N/A (Not crewmate but bacteria | Not named (French-Soviet manned flight in July 1982) |
| KETAMINE | Where Failure is not an Option - Personalized Medicine in Astronauts | 26489089 | pain / anesthesia | in-flight | Not one specific mission, but an accumulation of drugs aboard the ISS (as of 2014) | Unclear; not referenced specifically | ISS |
| L-ASPARTIC-*ta*-HYDROXAMATE | Cultivation in Space Flight Produces Minimal Alterations in the Susceptibility of Bacillus subtilis Cells to 72 Different Antibiotics and Growth-Inhibiting Compounds | 28821547 | bacterial infection | post-flight | Bacillus subtilis | 1 | ISS |
| LEVOFLOXACIN | Resilient microorganisms in dust samples of the International Space Station-survival of the adaptation specialists | 27998314 | bacterial infection | post-flight | 8-12 years old dust samples | 1 | Russian ISS Modules |

*Continued on next page*

Table S1 – *Continued from previous page*

| Drug Name | Publication | PMID | Condition | Timing | Mission | Crew | Location |
|---|---|---|---|---|---|---|---|
| LEVOFLOXACIN | Evaluation of Acquired Antibiotic Resistance in Escericihia coli Exposed to Long-Term Low-Shear Stress Modeled Microgravity and Background Antibiotic Exposure | 30647159 | E.coli infection | simulated microgravity | No specific mission (simulated microgravity) | N/A (not crew members, but bacterial strains) | N/A (simulated microgravity) |
| LEVOFLOXACIN | Evaluation of Physical and Chemical Changes in Pharmaceuticals Flown on Space Missions | 21479701 | infections | in-flight | ISS payloads | N/A (these drugs were just stored, not administered) | ISS |
| LEVOTHYROXINE | Evaluation of Physical and Chemical Changes in Pharmaceuticals Flown on Space Missions | 21479701 | hypothyroidism | in-flight | ISS / STS-122 | N/A (these drugs were just stored, not administered) | ISS |
| LIDOCAINE | Where Failure is not an Option - Personalized Medicine in Astronauts | 26489089 | anesthaetic | in-flight | Not one specific mission, but an accumulation of drugs aboard the ISS (as of 2014) | Unclear; not referenced specifically | ISS |
| LIDOCAINE | Evaluation of Physical and Chemical Changes in Pharmaceuticals Flown on Space Missions | 21479701 | pain prevention | in-flight | ISS payloads | N/A (these drugs were just stored, not administered) | ISS |
| LINCOMYCIN | Cultivation in Space Flight Produces Minimal Alterations in the Susceptibility of Bacillus subtilis Cells to 72 Different Antibiotics and Growth-Inhibiting Compounds | 28821547 | bacterial infection | post-flight | Bacillus subtilis | 1 | ISS |
| LINEZOLID | Resilient microorganisms in dust samples of the International Space Station-survival of the adaptation specialists | 27998314 | bacterial infection | post-flight | 8-12 years old dust samples | 1 | Russian ISS Modules |
| LOMEFLOXACIN | Cultivation in Space Flight Produces Minimal Alterations in the Susceptibility of Bacillus subtilis Cells to 72 Different Antibiotics and Growth-Inhibiting Compounds | 28821547 | bacterial infection | post-flight | Bacillus subtilis | 1 | ISS |
| LOPERAMIDE | Where Failure is not an Option - Personalized Medicine in Astronauts | 26489089 | diarrhea | in-flight | Not one specific mission, but an accumulation of drugs aboard the ISS (as of 2014) | Unclear; not referenced specifically | ISS |
| LOPERAMIDE | Chemical Potency and Degradation Products of Medications Stored Over 550 Earth Days at the International Space Station | 26546565 | antidiarrheal | tested post-flight | Sent via Russian Progress vehicle; returned via SpaceX Dragon Capsule | N/A (drugs were not administered to patients, but tested for efficacy) | ISS / ground |
| LORATADINE | Where Failure is not an Option - Personalized Medicine in Astronauts | 26489089 | vomiting / allergic rhinitis / urticaria | in-flight | Not one specific mission, but an accumulation of drugs aboard the ISS (as of 2014) | Unclear; not referenced specifically | ISS |
| LORATIDINE | Chemical Potency and Degradation Products of Medications Stored Over 550 Earth Days at the International Space Station | 26546565 | antihistamine | tested post-flight | Sent via Russian Progress vehicle; returned via SpaceX Dragon Capsule | N/A (drugs were not administered to patients, but tested for efficacy) | ISS / ground |
| MAGNESIUM ASPERTATE-; POTASSIUM ASPERTATE | Chapter 4 Pharmacology in Space: Pharmacotherapy | 9048135 | supplies potassium and other minerals | in-flight | 166-day MIR mission | 2 | MIR Space Station |
| MANGANESE (II) CHLORIDE | Cultivation in Space Flight Produces Minimal Alterations in the Susceptibility of Bacillus subtilis Cells to 72 Different Antibiotics and Growth-Inhibiting Compounds | 28821547 | bacterial infection | post-flight | Bacillus subtilis | 1 | ISS |
| MANGANESE CHLORIDE | Cultivation in Space Flight Produces Minimal Alterations in the Susceptibility of Bacillus subtilis Cells to 72 Different Antibiotics and Growth-Inhibiting Compounds | 28821547 | bacterial infection | post-flight | Bacillus subtilis | 1 | ISS |
| MECLIZINE | Medication Use by US Crewmembers on the International Space Station | 26187345 | SAS (space adaptation syndrome) | in-flight | Various missions from 2002-2012 (mix of short and long duration spaceflight) | 24 | ISS |
| MECLIZINE | Where Failure is not an Option - Personalized Medicine in Astronauts | 26489089 | vomiting / allergic rhinitis | in-flight | Not one specific mission, but an accumulation of drugs aboard the ISS (as of 2014) | Unclear; not referenced specifically | ISS |

*Continued on next page*

Table S1 – *Continued from previous page*

| Drug Name | Publication | PMID | Condition | Timing | Mission | Crew | Location |
|---|---|---|---|---|---|---|---|
| MELATONIN | Medication Use by US Crewmembers on the International Space Station | 26187345 | sleep irregularities / slam shift | in-flight | Various missions from 2002-2012 (mix of short and long duration spaceflight) | 24 | ISS |
| MELATONIN | Prevalence of Sleep Deficiency and use of hypnotic drugs in astronauts before, during, and after spaceflight: an observational study | 25127232 | irregular sleep | in-flight | Various space shuttle missions | 61 of the 78 crewmembers | Space shuttles |
| MELATONIN | Where Failure is not an Option - Personalized Medicine in Astronauts | 26489089 | insomnia | in-flight | Not one specific mission, but an accumulation of drugs aboard the ISS (as of 2014) | Unclear; not referenced specifically | ISS |
| MELATONIN | Chemical Potency and Degradation Products of Medications Stored Over 550 Earth Days at the International Space Station | 26546565 | insomnia | tested post-flight | Sent via Russian Progress vehicle; returned via SpaceX Dragon Capsule | N/A (drugs were not administered to patients, but tested for efficacy) | ISS / ground |
| MELATONIN | Sleep, performance, circadian rhythms, and light-dark cycles during two space shuttle flights | 11641138 | Sleep Disturbance | in-flight | STS 90/95 | 5 | STS 90/95 Space Shuttle |
| MELATONIN | Melatonin attenuates the sympathetic nerve responses to orthostatic stress in humans | 12869610 | decrease in muscle sympathetic nerve activity | In-flight | Did not specify | 12 | ISS |
| MEROPENEM | Multi-resistant biofilm-forming pathogens on the International Space Station | 31719234 | bacterial infection | in-flight | 2013-2015 - S. hominis | 1 | ISS |
| MEROPENEM | Multi-resistant biofilm-forming pathogens on the International Space Station | 31719234 | bacterial infection | in-flight | 2013-2015 - E. faecalis | 1 | ISS |
| MEROPENEM | Resilient microorganisms in dust samples of the International Space Station-survival of the adaptation specialists | 27998314 | bacterial infection | post-flight | 8-12 years old dust samples | 1 | Russian ISS Modules |
| METOCLOPRAMIDE | Factors Affecting Drug Bioavailability in Space | 8083399 | gastrointestinal discomfort | in-flight | First 33 space shuttle missions | A specific number is unclear, but 500 individual doses were administered over 83% of the crewmembers | 33 space shuttles |
| METOPROLOL | Where Failure is not an Option - Personalized Medicine in Astronauts | 26489089 | heart failure / hypertension | in-flight | Not one specific mission, but an accumulation of drugs aboard the ISS (as of 2014) | Unclear; not referenced specifically | ISS |
| METOPROLOL SUCCINATE | Evaluation of Physical and Chemical Changes in Pharmaceuticals Flown on Space Missions | 21479701 | high blood pressure and chest pain | in-flight | ISS payloads | N/A (these drugs were just stored, not administered) | ISS |
| METRONIDAZOLE | Evaluation of Physical and Chemical Changes in Pharmaceuticals Flown on Space Missions | 21479701 | infections | in-flight | ISS payloads | N/A (these drugs were just stored, not administered) | ISS |
| METRYAPONE | Dexamethasone mimicks the antimotion sickness effects of amphetamine and scopolamine | 11538662 | space motion sickness | rotating chair model | | 19 | ground-study |
| MINOCYCLINE | Multidrug-resistant Acinetobacter pittii is adapting to and exhibiting potential succession aboard the International Space Station | 36503581 | bacterial infection | post-flight | A. pittii | 1 | ISS |
| MINOCYCLINE | Cultivation in Space Flight Produces Minimal Alterations in the Susceptibility of Bacillus subtilis Cells to 72 Different Antibiotics and Growth-Inhibiting Compounds | 28821547 | bacterial infection | post-flight | Bacillus subtilis | 1 | ISS |
| MODAFINIL | Prevalence of Sleep Deficiency and use of hypnotic drugs in astronauts before, during, and after spaceflight: an observational study (NOTE: this is from the supplemental materials section) | 25127232 | irregular sleep / alertness | in-flight | Various space shuttle missions | 10 shuttle crewmembers | Space shuttles |
| MODAFINIL | Chemical Potency and Degradation Products of Medications Stored Over 550 Earth Days at the International Space Station | 26546565 | alertness | tested post-flight | Sent via Russian Progress vehicle; returned via SpaceX Dragon Capsule | N/A (drugs were not administered to patients, but tested for efficacy) | ISS / ground |
| MOXALACTAM | Cultivation in Space Flight Produces Minimal Alterations in the Susceptibility of Bacillus subtilis Cells to 72 Different Antibiotics and Growth-Inhibiting Compounds | 28821547 | bacterial infection | post-flight | Bacillus subtilis | 1 | ISS |

*Continued on next page*

Table S1 – *Continued from previous page*

| Drug Name | Publication | PMID | Condition | Timing | Mission | Crew | Location |
|---|---|---|---|---|---|---|---|
| MUPIROCIN | Evaluation of Physical and Chemical Changes in Pharmaceuticals Flown on Space Missions | 21479701 | skin infections | in-flight | ISS payloads | N/A (these drugs were just stored, not administered) | ISS |
| NAFCILLIN | Cultivation in Space Flight Produces Minimal Alterations in the Susceptibility of Bacillus subtilis Cells to 72 Different Antibiotics and Growth-Inhibiting Compounds | 28821547 | bacterial infection | post-flight | Bacillus subtilis | 1 | ISS |
| NALIDIXIC ACID | Novel Antibiotic Testing Approaches Reveal Reduced Antibiotic Efficacy Against Shiga Toxin-Producing Escherichia coli O157:H7 Under Simulated Microgravity | 30619237 | bacterial infection | RCSS simulation | | Shiga Toxin-Producing Escherichia coli O157:H7 | RCSS simulation |
| NALIDIXIC ACID | Cultivation in Space Flight Produces Minimal Alterations in the Susceptibility of Bacillus subtilis Cells to 72 Different Antibiotics and Growth-Inhibiting Compounds | 28821547 | bacterial infection | post-flight | Bacillus subtilis | 1 | ISS |
| NALOXONE HYDROCHLORIDE | Factors Affecting Drug Bioavailability in Space | 8083399 | reverse effects of opioids | in-flight | First 33 space shuttle missions | A specific number is unclear, but 500 individual doses were administered over 83% of the crewmembers | 33 space shuttles |
| NEOMYCIN | Cultivation in Space Flight Produces Minimal Alterations in the Susceptibility of Bacillus subtilis Cells to 72 Different Antibiotics and Growth-Inhibiting Compounds | 28821547 | bacterial infection | post-flight | Bacillus subtilis | 1 | ISS |
| NICKEL CHLORIDE | Cultivation in Space Flight Produces Minimal Alterations in the Susceptibility of Bacillus subtilis Cells to 72 Different Antibiotics and Growth-Inhibiting Compounds | 28821547 | bacterial infection | post-flight | Bacillus subtilis | 1 | ISS |
| NITROFURANTOIN | Chapter 4 Pharmacology in Space: Pharmacotherapy | 9048135 | urinary tract infection ; Pseudomonas aerogunisae | post-flight | Apollo 13 | 1 | Moon Landing |
| NITROFURANTOIN | Evaluation of Acquired Antibiotic Resistance in Escericihia coli Exposed to Long-Term Low-Shear Stress Modeled Microgravity and Background Antibiotic Exposure | 30647159 | E.coli infection | simulated microgravity | No specific mission (simulated microgravity) | N/A (not crew members, but bacterial strains) | N/A (simulated microgravity) |
| NOOTROPLY-2--OXO-1-PYRROLIDINYL-ACETAMIDE | Chapter 4 Pharmacology in Space: Pharmacotherapy | 9048135 | increases myofibril potential | in-flight | 166-day MIR mission | 2 | MIR Space Station |
| NOVOBIOCIN (OR ALBAMYCIN OR CATHOMYCIN) | Cultivation in Space Flight Produces Minimal Alterations in the Susceptibility of Bacillus subtilis Cells to 72 Different Antibiotics and Growth-Inhibiting Compounds | 28821547 | bacterial infection | post-flight | Bacillus subtilis | 1 | ISS |
| OFLOXACIN | Cultivation in Space Flight Produces Minimal Alterations in the Susceptibility of Bacillus subtilis Cells to 72 Different Antibiotics and Growth-Inhibiting Compounds | 28821547 | bacterial infection | post-flight | Bacillus subtilis | 1 | ISS |
| OMEPRAZOLE | A case of persistent skin rash and rhinitis with immune system dysregulation onboard the International Space Station | 27036643 | Rash, relieve itching/allergic reactions | in-flight | ISS Expedition 16-31/32 (Not exactly sure which one as NASA lists all these expedition as associated with study). Paper mentions Soyuz (not exactly sure which specific Soyuz mission). Mentions a 191 flight day mission | 1 | ISS/Soyuz |
| OMEPRAZOLE | Where Failure is not an Option - Personalized Medicine in Astronauts | 26489089 | reflux | in-flight | Not one specific mission, but an accumulation of drugs aboard the ISS (as of 2014) | Unclear; not referenced specifically | ISS |
| ONDANSETRON | Where Failure is not an Option - Personalized Medicine in Astronauts | 26489089 | vomiting | in-flight | Not one specific mission, but an accumulation of drugs aboard the ISS (as of 2014) | Unclear; not referenced specifically | ISS |

*Continued on next page*

Table S1 – *Continued from previous page*

| Drug Name | Publication | PMID | Condition | Timing | Mission | Crew | Location |
|---|---|---|---|---|---|---|---|
| OXACILLIN | Multi-resistant biofilm-forming pathogens on the International Space Station | 31719234 | bacterial infection | in-flight | Salyut 7 | 1 | Salyut 7 |
| OXACILLIN | Detection of antimicrobial resistance genes associated with the International Space Station environmental surfaces | 29339831 | bacterial infection | post-flight | Fifty-seven Biosafety Level-2 (BSL-2) microorganisms | 1 | ISS |
| OXACILLIN | Cultivation in Space Flight Produces Minimal Alterations in the Susceptibility of Bacillus subtilis Cells to 72 Different Antibiotics and Growth-Inhibiting Compounds | 28821547 | bacterial infection | post-flight | Bacillus subtilis | 1 | ISS |
| OXACILLIN | Study of minimal inhibitory concentration of antibiotics on bacteria cultivated in vitro in space (Cytos 2 experiment) | 3899095 | Weak increase in Staphylococcus aureus resistance to antibiotics | in-flight | Cytos 2 | N/A (Not crewmate but bacteria | Not named (French-Soviet manned flight in July 1982) |
| OXOLINIC ACID | Cultivation in Space Flight Produces Minimal Alterations in the Susceptibility of Bacillus subtilis Cells to 72 Different Antibiotics and Growth-Inhibiting Compounds | 28821547 | bacterial infection | post-flight | Bacillus subtilis | 1 | ISS |
| OXYMETAZOLINE | Pharmaceutical Use by US Astronauts on Space Shuttle Missions (Source: "Supplying a Pharmacy for NASA Exploration Spaceflight: Challenges and Current Understanding") | 10417009 (31231-676) | congestion | in-flight | 79 space shuttle missions | 219 | Space shuttles |
| OXYMETAZOLINE HYDRROCHLORIDE | Factors Affecting Drug Bioavailability in Space | 8083399 | congestion | in-flight | First 33 space shuttle missions | A specific number is unclear, but 500 individual doses were administered over 83% of the crewmembers | 33 space shuttles |
| PAROMOMYCIN | Cultivation in Space Flight Produces Minimal Alterations in the Susceptibility of Bacillus subtilis Cells to 72 Different Antibiotics and Growth-Inhibiting Compounds | 28821547 | bacterial infection | post-flight | Bacillus subtilis | 1 | ISS |
| PENICILLIN | Simulated microgravity affects some biological characteristics of Lactobacillus acidophilus | 28013406 | bacterial infection | RCSS simulation | | Lactobacillus acidophilus | RCSS simulation |
| PENICILLIN | Multi-resistant biofilm-forming pathogens on the International Space Station | 31719234 | bacterial infection | in-flight | Permanent Multipurpose Module - S. Aureus | 1 | ISS |
| PENICILLIN | Resilient microorganisms in dust samples of the International Space Station-survival of the adaptation specialists | 27998314 | bacterial infection | post-flight | 8-12 years old dust samples | 1 | Russian ISS Modules |
| PENICILLIN | Detection of antimicrobial resistance genes associated with the International Space Station environmental surfaces | 29339831 | bacterial infection | post-flight | Fifty-seven Biosafety Level-2 (BSL-2) microorganisms | 1 | ISS |
| PENIMEPICYCLINE | Cultivation in Space Flight Produces Minimal Alterations in the Susceptibility of Bacillus subtilis Cells to 72 Different Antibiotics and Growth-Inhibiting Compounds | 28821547 | bacterial infection | post-flight | Bacillus subtilis | 1 | ISS |
| PHENAZOPYRIDINE | Chapter 4 Pharmacology in Space: Pharmacotherapy | 9048135 | urinary tract infection ; Pseudomonas aerogunisae | post-flight | Apollo 13 | 1 | Moon Landing |
| PHENYLEPHRINE; PHENYLPROPANOLAMINE | Pharmaceutical Use by US Astronauts on Space Shuttle Missions (Source: "Supplying a Pharmacy for NASA Exploration Spaceflight: Challenges and Current Understanding") | 10417009 (31231-676) | congestion | in-flight | 79 space shuttle missions | 219 | Space shuttles |
| PHENYTOIN | Where Failure is not an Option - Personalized Medicine in Astronauts | 26489089 | epilepsia / seizures | in-flight | Not one specific mission, but an accumulation of drugs aboard the ISS (as of 2014) | Unclear; not referenced specifically | ISS |
| PHENYTOIN | Evaluation of Physical and Chemical Changes in Pharmaceuticals Flown on Space Missions | 21479701 | epilepsy / seizures | in-flight | ISS payloads | N/A (these drugs were just stored, not administered) | ISS |
| PIPERACILLIM | Multidrug-resistant Acinetobacter pittii is adapting to and exhibiting potential succession aboard the International Space Station | 36503581 | bacterial infection | post-flight | A. pittii | 1 | ISS |

*Continued on next page*

Table S1 – *Continued from previous page*

| Drug Name | Publication | PMID | Condition | Timing | Mission | Crew | Location |
|---|---|---|---|---|---|---|---|
| POLYMIXIN B, 2,4-DIAMINO-6,7--DIISOPROPYL-PTERIDINE | Cultivation in Space Flight Produces Minimal Alterations in the Susceptibility of Bacillus subtilis Cells to 72 Different Antibiotics and Growth-Inhibiting Compounds | 28821547 | bacterial infection | post-flight | Bacillus subtilis | 1 | ISS |
| POTASSIUM CHLORIDE | Chapter 4 Pharmacology in Space: Pharmacotherapy | 9048135 | cardiac arrhythmia | in-flight | Apollo 16 and 17 | 6 | Moon Landing |
| POTASSIUM CHROMATE | Cultivation in Space Flight Produces Minimal Alterations in the Susceptibility of Bacillus subtilis Cells to 72 Different Antibiotics and Growth-Inhibiting Compounds | 28821547 | bacterial infection | post-flight | Bacillus subtilis | 1 | ISS |
| POTASSIUM OROTATE | Chapter 4 Pharmacology in Space: Pharmacotherapy | 9048135 | nonsteroidal anabolic drug | in-flight | 166-day MIR mission | 2 | MIR Space Station |
| POTASSIUM TELLURITE | Cultivation in Space Flight Produces Minimal Alterations in the Susceptibility of Bacillus subtilis Cells to 72 Different Antibiotics and Growth-Inhibiting Compounds | 28821547 | bacterial infection | post-flight | Bacillus subtilis | 1 | ISS |
| PROCHLORPERAZINE | Factors Affecting Drug Bioavailability in Space | 8083399 | nausea / vomiting (possibly SMS) | in-flight | First 33 space shuttle missions | A specific number is unclear, but 500 individual doses were administered over 83% of the crewmembers | 33 space shuttles |
| PROGESTIN / ESTROGEN | Evaluation of Physical and Chemical Changes in Pharmaceuticals Flown on Space Missions | 21479701 | contraceptive | in-flight | ISS payloads | N/A (these drugs were just stored, not administered) | ISS |
| PROGESTIN / ESTROGEN | Albumin, oral contraceptives, and venous thromboembolism risk in astronauts | 35389755 | higher serum concentrations of the acute phase reactant ceruloplasmin | In-flight | various ISS expeditions | 6 (females) | ISS |
| PROGESTIN / ESTROGEN | Albumin, oral contraceptives, and venous thromboembolism risk in astronauts | 35389755 | lower concentrations of serum albumin | In-flight | various ISS expeditions | 6 (females) | ISS |
| PROGESTIN / ESTROGEN | Albumin, oral contraceptives, and venous thromboembolism risk in astronauts | 35389755 | higher concentrations of transferrin | In-flight | various ISS expeditions | 6 (females) | ISS |
| PROMETHAZINE | Evaluation of Physical and Chemical Changes in Pharmaceuticals Flown on Space Missions | 21479701 | allergies | in-flight | ISS / STS-126 | N/A (these drugs were just stored, not administered) | ISS |
| PROMETHAZINE | Chapter 4 Pharmacology in Space: Pharmacotherapy | 9048135 | space motion sickness | in-flight | Space Shuttles | 29 | Space Shuttle |
| PROMETHAZINE | Medication Use by US Crewmembers on the International Space Station | 26187345 | SAS (space adaptation syndrome) | in-flight | Various missions from 2002-2012 (mix of short and long duration spaceflight) | 24 | ISS |
| PROMETHAZINE | Comparison of Treatment Strategies for Space Motion Sickness | 11541442 | SMS (space motion sickness) | in-flight | Various space shuttle missions | 15 | Space shuttles |
| PROMETHAZINE | Pharmaceutical Use by US Astronauts on Space Shuttle Missions (Source: "Supplying a Pharmacy for NASA Exploration Spaceflight: Challenges and Current Understanding") | 10417009 (31231-676) | SMS (space motion sickness) | in-flight | 79 space shuttle missions | 219 | Space shuttles |
| PROMETHAZINE | Where Failure is not an Option - Personalized Medicine in Astronauts | 26489089 | rhinitis / urticaria / sedation / vomiting | in-flight | Not one specific mission, but an accumulation of drugs aboard the ISS (as of 2014) | Unclear; not referenced specifically | ISS |
| PROMETHAZINE HYDROCHLORIDE | Factors Affecting Drug Bioavailability in Space | 8083399 | SMS (space motion sickness) | in-flight | First 33 space shuttle missions | A specific number is unclear, but 500 individual doses were administered over 83% of the crewmembers | 33 space shuttles |
| PROMETHAZINE; DEXTROAMPHETAMINE | Pharmaceutical Use by US Astronauts on Space Shuttle Missions (Source: "Supplying a Pharmacy for NASA Exploration Spaceflight: Challenges and Current Understanding") | 10417009 (31231-676) | SMS (space motion sickness) | in-flight | 79 space shuttle missions | 219 | Space shuttles |
| PROPOFOL | Drugs in Space: Pharmacokinetics and Pharmacodynamics in Astronauts (however, this specific drug was taken from a study cited in the text titled "Effects of Simulated Microgravity on the Anesthetic Properties of Propofol") | 28533143 | anesthetic agent | simulated microgravity (2 day head-down bed rest (HDBR)) | No specific mission (simulated microgravity) | Did not specify | On ground (head-down bed rest) |

*Continued on next page*

Table S1 – *Continued from previous page*

| Drug Name | Publication | PMID | Condition | Timing | Mission | Crew | Location | |
|---|---|---|---|---|---|---|---|---|
| PROTAMINE (SULFATE?) | Venous Thrombosis during Spaceflight | 31893522 | venous thr-ombosis (blood clots) | In-flight | Did not specify | Did not specify | ISS | |
| PROTHROMBIN | Venous Thrombosis during Spaceflight | 31893522 | venous thr-ombosis (blood clots) | In-flight | Did not specify | Did not specify | ISS | |
| PSEUDOEPHEDR-INE | Pharmaceutical Use by US Astronauts on Space Shuttle Missions (Source: "Supplying a Pharmacy for NASA Explorati-on Spaceflight: Challenges and Current Understanding") | 10417009 (31231-676) | congestion / allergies | in-flight | 79 space shuttle missions | 219 | Space shuttles | |
| PSEUDOEPHEDR-INE | Factors Affecting Drug Bioavaila-bility in Space | 8083399 | congestion | in-flight | First 33 space shuttle missions | A specific number is unclear, but 500 ind-ividual doses were administered over 83% of the crewmembers | 33 space shuttles | |
| PSEUDOEPHEDR-INE HYDROCHLO-RIDE | Chemical Potency and Degrad-ation Products of Medications Stored Over 550 Earth Days at the International Space Station | 26546565 | decongestant | tested pos-t-flight | Sent via Russian Progress vehicl-e; returned via SpaceX Dragon Capsule | N/A (drugs were not administered to pat-ients, but tested for efficacy) | ISS / ground | |
| QUETIAPINE FUMARATE | Prevalence of Sleep Deficiency and use of hypnotic drugs in ast-ronauts before, during, and after spaceflight: an observational study | 25127232 | irregular sleep | in-flight | Various space shuttle missions | 61 of the 78 crewme-mbers | Space shuttles | |
| RIBOXIN | Chapter 4 Pharmacology in Space: Pharmacotherapy | 9048135 | myocardia-l metabolic disorders | in-flight | 166-day MIR mission | 2 | MIR Space Station | |
| RIFAMPIN | Responses of haloarchaea to simulated microgravity | 21417742 | archea resistance | RCSS sim-ulation | | Haloferax mediterranei | RCSS simulation | |
| RIFAMPIN | Multi-resistant biofilm-forming pathogens on the International Space Station | 31719234 | bacterial infect-ion | in-flight | Permanent Mul-tipurpose Module - S. Aureus | 1 | ISS | |
| RIFAMPIN | Detection of antimicrobial res-istance genes associated with the International Space Station environmental surfaces | 29339831 | bacterial infect-ion | post-flight | Fifty-seven Biosafety Lev-el-2 (BSL-2) microorganisms | 1 | ISS | |
| RIFAMPIN | Cultivation in Space Flight Pro-duces Minimal Alterations in the Susceptibility of Bacillus subtilis Cells to 72 Different Antibi-otics and Growth-Inhibiting Compounds | 28821547 | bacterial infect-ion | post-flight | Bacillus subtilis | 1 | ISS | |
| RISEDRONIC ACID | Evaluation of Physical and Chemical Changes in Pha-rmaceuticals Flown on Space Missions | 21479701 | osteoporosis | in-flight | ISS payloads | N/A (these drugs were just stored, not administered) | ISS | |
| ROLITETRACYC-LINE | Cultivation in Space Flight Pro-duces Minimal Alterations in the Susceptibility of Bacillus subtilis Cells to 72 Different Antibi-otics and Growth-Inhibiting Compounds | 28821547 | bacterial infect-ion | post-flight | Bacillus subtilis | 1 | ISS | |
| SCOPOLAMINE | Medication Use by US Crewme-mbers on the International Space Station | 26187345 | SAS (space adaptation syndrome) | in-flight | Various missions from 2002-2012 (mix of short and long duration spaceflight) | 24 | ISS | |
| SCOPOLAMINE | Pharmacokinetic Consequences of Spaceflight | 11537657 | pain relief | In-flight | Did not specify | Did not specify | Did not specify | |
| SCOPOLAMINE HYDROBROMIDE | Factors Affecting Drug Bioavaila-bility in Space | 8083399 | nausea / vom-iting (possibly SMS) | in-flight | First 33 space shuttle missions | A specific number is unclear, but 500 ind-ividual doses were administered over 83% of the crewmembers | 33 space shuttles | |
| SCOPOLAMINE HYDROBROM-IDE; DEXTRO-AMPHETAMINE SULFATE | Factors Affecting Drug Bioavaila-bility in Space | 8083399 | SMS (space motion sickness) | in-flight | First 33 space shuttle missions | A specific number is unclear, but 500 ind-ividual doses were administered over 83% of the crewmembers | 33 space shuttles | |
| SCOPOLAMINE; DEXTROAMPHET-AMINE | Chapter 4 Pharmacology in Space: Pharmacotherapy | 9048135 | space motion sickness | in-flight | post Apollo 11 | ? | Orbital Flights + Moon Landing | |
| SCOPOLAMINE; DEXTROAMPHET-AMINE | Comparison of Treatment Strate-gies for Space Motion Sickness | 11541442 | SMS (space motion sickness) | in-flight (and pre-fl-ight); used prophylac-tically | Various space shuttle missions | 19 | Space shuttles | |
| SERTRALINE | Where Failure is not an Opt-ion - Personalized Medicine in Astronauts | 26489089 | depression | in-flight | Not one specific mission, but an accumulation of drugs aboard the ISS (as of 2014) | Unclear; not referenced specifically | ISS | |

*Continued on next page*

Table S1 – *Continued from previous page*

| Drug Name | Publication | PMID | Condition | Timing | Mission | Crew | Location |
|---|---|---|---|---|---|---|---|
| SERTRALINE | Evaluation of Physical and Chemical Changes in Pharmaceuticals Flown on Space Missions | 21479701 | depression and other disorders treatable with SSRIs | in-flight | ISS payloads | N/A (these drugs were just stored, not administered) | ISS |
| SILVER SULFADIAZINE | Evaluation of Physical and Chemical Changes in Pharmaceuticals Flown on Space Missions | 21479701 | topical antibiotic | in-flight | ISS payloads | N/A (these drugs were just stored, not administered) | ISS |
| SIMETHECONE | Pharmaceutical Use by US Astronauts on Space Shuttle Missions (Source: "Supplying a Pharmacy for NASA Exploration Spaceflight: Challenges and Current Understanding") | 10417009 (31231-676) | gastrointestinal discomfort | in-flight | 79 space shuttle missions | 219 | Space shuttles |
| SISOMICIN | Cultivation in Space Flight Produces Minimal Alterations in the Susceptibility of Bacillus subtilis Cells to 72 Different Antibiotics and Growth-Inhibiting Compounds | 28821547 | bacterial infection | post-flight | Bacillus subtilis | 1 | ISS |
| SPECTINOMYCIN | Cultivation in Space Flight Produces Minimal Alterations in the Susceptibility of Bacillus subtilis Cells to 72 Different Antibiotics and Growth-Inhibiting Compounds | 28821547 | bacterial infection | post-flight | Bacillus subtilis | 1 | ISS |
| SPIRAMYCIN | Cultivation in Space Flight Produces Minimal Alterations in the Susceptibility of Bacillus subtilis Cells to 72 Different Antibiotics and Growth-Inhibiting Compounds | 28821547 | bacterial infection | post-flight | Bacillus subtilis | 1 | ISS |
| SPOPOLAMIDE / DEXTROAMPHETAMINE | Comparison of Treatment Strategies for Space Motion Sickness | 11541442 | space motion sickness | In-flight | STS-26 | 5 | Shuttle |
| STREPTOMYCIN | Multi-resistant biofilm-forming pathogens on the International Space Station | 31719234 | bacterial infection | in-flight | filter samples | 1 | ISS |
| SULFADIAZINE | Cultivation in Space Flight Produces Minimal Alterations in the Susceptibility of Bacillus subtilis Cells to 72 Different Antibiotics and Growth-Inhibiting Compounds | 28821547 | bacterial infection | post-flight | Bacillus subtilis | 1 | ISS |
| SULFAMETHAZINE | Cultivation in Space Flight Produces Minimal Alterations in the Susceptibility of Bacillus subtilis Cells to 72 Different Antibiotics and Growth-Inhibiting Compounds | 28821547 | bacterial infection | post-flight | Bacillus subtilis | 1 | ISS |
| SULFAMETHOXAZOLE | Where Failure is not an Option - Personalized Medicine in Astronauts | 26489089 | antiobiotic | in-flight | Not one specific mission, but an accumulation of drugs aboard the ISS (as of 2014) | Unclear; not referenced specifically | ISS |
| SULFAMETHOXAZOLE | Multi-resistant biofilm-forming pathogens on the International Space Station | 31719234 | bacterial infection | in-flight | 2013-2015 - S. Aureus | 1 | ISS |
| SULFAMETHOXAZOLE | Multi-resistant biofilm-forming pathogens on the International Space Station | 31719234 | bacterial infection | in-flight | 2013-2015 - S. epidermidis | 1 | ISS |
| SULFAMETHOXAZOLE | Multi-resistant biofilm-forming pathogens on the International Space Station | 31719234 | bacterial infection | in-flight | 2013-2015 - E. faecalis | 1 | ISS |
| SULFAMETHOXAZOLE | Multi-resistant biofilm-forming pathogens on the International Space Station | 31719234 | bacterial infection | in-flight | 2013-2015 - diverse surfaces | 1 | ISS |
| SULFAMETHOXAZOLE | Cultivation in Space Flight Produces Minimal Alterations in the Susceptibility of Bacillus subtilis Cells to 72 Different Antibiotics and Growth-Inhibiting Compounds | 28821547 | bacterial infection | post-flight | Bacillus subtilis | 1 | ISS |
| SULFATHIAZOLE | Cultivation in Space Flight Produces Minimal Alterations in the Susceptibility of Bacillus subtilis Cells to 72 Different Antibiotics and Growth-Inhibiting Compounds | 28821547 | bacterial infection | post-flight | Bacillus subtilis | 1 | ISS |
| TAMSULOSIN | Where Failure is not an Option - Personalized Medicine in Astronauts | 26489089 | prostate hyperplasia | in-flight | Not one specific mission, but an accumulation of drugs aboard the ISS (as of 2014) | Unclear; not referenced specifically | ISS |

*Continued on next page*

Table S1 – *Continued from previous page*

| Drug Name | Publication | PMID | Condition | Timing | Mission | Crew | Location | |
|---|---|---|---|---|---|---|---|---|
| TEMAZEPAM | Temazepam, but not zolpidem, causes orthostatic hypotension in astronauts after spaceflight | 12500019 | orthostatic hypotension | in-flight ; night before landing | 7-year period from January 1990 until December 1996 | 57 | space exposure | |
| TEMAZEPAM | Pharmaceutical Use by US Astronauts on Space Shuttle Missions (Source: "Supplying a Pharmacy for NASA Explorati-on Spaceflight: Challenges and Current Understanding") | 10417009 (31231-676) | insomnia | in-flight | 79 space shuttle missions | 219 | Space shuttles | |
| TEMAZEPAM | Prevalence of Sleep Deficiency and use of hypnotic drugs in ast-ronauts before, during, and after spaceflight: an observational study | 25127232 | irregular sleep | in-flight | Various space shuttle missions | 61 of the 78 crewme-mbers | Space shuttles | |
| TEMAZOPAM | Evaluation of Physical and Chemical Changes in Pha-rmaceuticals Flown on Space Missions | 21479701 | insomnia | in-flight | ISS payloads | N/A (these drugs were just stored, not administered) | ISS | |
| TERBINAFINE | A case of persistent skin rash and rhinitis with immune sys-tem dysregulation onboard the International Space Station | 27036643 | fungal infection | in-flight | ISS Expedition 16-31/32 (Not exactly sure which one as NASA lists all these expedi-tion as associated with study). Paper mentions Soyuz (not exactly sure which specific Soyuz mission). Mentions a 191 flight day mission | 1 | ISS/Soyuz | |
| TETRACYCLINE | Chapter 4 Pharmacology in Space: Pharmacotherapy | 9048135 | urinary tra-ct infection ; Pseudomonas aerogunisae | in-flight | Apollo 13 | 1 | Moon Landing | |
| TETRACYCLINE | Immunity in Space: Prokar-yote Adaptations and Immune Response in Microgravity | 33540536 | bacterial infect-ion | RCSS sim-ulation | | e.coli | RCSS simulation for 1000 generations | |
| TETRACYCLINE | Multi-resistant biofilm-forming pathogens on the International Space Station | 31719234 | bacterial infect-ion | in-flight | filter samples | 1 | ISS | |
| TETRACYCLINE | Multi-resistant biofilm-forming pathogens on the International Space Station | 31719234 | bacterial infect-ion | in-flight | 2013-2015 - S. Aureus | 1 | ISS | |
| TETRACYCLINE | Multi-resistant biofilm-forming pathogens on the International Space Station | 31719234 | bacterial infect-ion | in-flight | 2013-2015 - S. hominis | 1 | ISS | |
| TETRACYCLINE | Multi-resistant biofilm-forming pathogens on the International Space Station | 31719234 | bacterial infect-ion | in-flight | 2013-2015 - E. faecalis | 1 | ISS | |
| TETRACYCLINE | Cultivation in Space Flight Pro-duces Minimal Alterations in the Susceptibility of Bacillus subtilis Cells to 72 Different Antibi-otics and Growth-Inhibiting Compounds | 28821547 | bacterial infect-ion | post-flight | Bacillus subtilis | 1 | ISS | |
| TETRACYCLINE | Evaluation of Acquired Antibi-otic Resistance in Escericihia coli Exposed to Long-Term Low-Sh-ear Stress Modeled Microgravity and Background Antibiotic Exposure | 30647159 | E.coli resistance | simulated micrograv-ity | No specific mis-sion (simulated microgravity) | N/A (not crew mem-bers, but bacterial strains) | N/A (si-mulated microg-ravity) | |
| THALLIUM (I) ACETATE | Cultivation in Space Flight Pro-duces Minimal Alterations in the Susceptibility of Bacillus subtilis Cells to 72 Different Antibi-otics and Growth-Inhibiting Compounds | 28821547 | bacterial infect-ion | post-flight | Bacillus subtilis | 1 | ISS | |
| TIGECYCLINE | Multi-resistant biofilm-forming pathogens on the International Space Station | 31719234 | bacterial infect-ion | in-flight | 2013-2015 - S. hominis | 1 | ISS | |
| TOBRAMYCIN | Multidrug-resistant Acinetoba-cter pittii is adapting to and exhibiting potential succession aboard the International Space Station | 36503581 | bacterial infect-ion | post-flight | A. pittii | 1 | ISS | |
| TOBRAMYCIN | Detection of antimicrobial res-istance genes associated with the International Space Station environmental surfaces | 29339831 | bacterial infect-ion | post-flight | Fifty-seven Biosafety Lev-el-2 (BSL-2) microorganisms | 1 | ISS | |

*Continued on next page*

Table S1 – *Continued from previous page*

| Drug Name | Publication | PMID | Condition | Timing | Mission | Crew | Location |
|---|---|---|---|---|---|---|---|
| TOBRAMYCIN | Cultivation in Space Flight Produces Minimal Alterations in the Susceptibility of Bacillus subtilis Cells to 72 Different Antibiotics and Growth-Inhibiting Compounds | 28821547 | bacterial infection | post-flight | Bacillus subtilis | 1 | ISS |
| TOBRAMYCIN | Evaluation of Acquired Antibiotic Resistance in Escericihia coli Exposed to Long-Term Low-Shear Stress Modeled Microgravity and Background Antibiotic Exposure | 30647159 | E.coli infection | simulated micrograv-ity | No specific mission (simulated microgravity) | N/A (not crew members, but bacterial strains) | N/A (simulated microg-ravity) |
| TRIAMCINOLONE | Evaluation of Physical and Chemical Changes in Pharmaceuticals Flown on Space Missions | 21479701 | corticosteroid | in-flight | ISS / STS-126 | N/A (these drugs were just stored, not administered) | ISS |
| TRIAMCINOLONE ACETONIDE | A case of persistent skin rash and rhinitis with immune system dysregulation onboard the International Space Station | 27036643 | Skin rash, dermatitis | in-flight | ISS Expedition 16-31/32 (Not exactly sure which one as NASA lists all these expedition as associated with study). Paper mentions Soyuz (not exactly sure which specific Soyuz mission). Mentions a 191 flight day mission | 1 | ISS/Soyuz |
| TRIAZOLAM | Factors Affecting Drug Bioavailability in Space | 8083399 | insomnia | in-flight | First 33 space shuttle missions | A specific number is unclear, but 500 individual doses were administered over 83% of the crewmembers | 33 space shuttles |
| TRIFLUOPERAZ-INE | Cultivation in Space Flight Produces Minimal Alterations in the Susceptibility of Bacillus subtilis Cells to 72 Different Antibiotics and Growth-Inhibiting Compounds | 28821547 | bacterial infection | post-flight | Bacillus subtilis | 1 | ISS |
| TRIMETHOPRIM | Multidrug-resistant Acinetobacter pittii is adapting to and exhibiting potential succession aboard the International Space Station | 36503581 | bacterial infection | post-flight | A. pittii | 1 | ISS |
| TRIMETHOPRIM | Resilient microorganisms in dust samples of the International Space Station-survival of the adaptation specialists | 27998314 | bacterial infection | post-flight | 8-12 years old dust samples | 1 | Russian ISS Modules |
| TRIMETHOPRIM / SULFAMETHAXO-ZOLE | Evaluation of Physical and Chemical Changes in Pharmaceuticals Flown on Space Missions | 21479701 | antibiotic | in-flight | ISS payloads | N/A (these drugs were just stored, not administered) | ISS |
| TRIMETHOPRIM / SULFAMETHOXA-ZOLE | Evaluation of Acquired Antibiotic Resistance in Escericihia coli Exposed to Long-Term Low-Shear Stress Modeled Microgravity and Background Antibiotic Exposure | 30647159 | E.coli infection | simulated micrograv-ity | No specific mission (simulated microgravity) | N/A (not crew members, but bacterial strains) | N/A (simulated microg-ravity) |
| TRIPROLIDINE; PSEUDOEPHEDR-INE | Factors Affecting Drug Bioavailability in Space | 8083399 | congestion | in-flight | First 33 space shuttle missions | A specific number is unclear, but 500 individual doses were administered over 83% of the crewmembers | 33 space shuttles |
| TYLOSIN | Cultivation in Space Flight Produces Minimal Alterations in the Susceptibility of Bacillus subtilis Cells to 72 Different Antibiotics and Growth-Inhibiting Compounds | 28821547 | bacterial infection | post-flight | Bacillus subtilis | 1 | ISS |
| VALACYCLOVIR | A case of persistent skin rash and rhinitis with immune system dysregulation onboard the International Space Station | 27036643 | antiviral medication (herpesviral reactivtivation) | in-flight | ISS Expedition 16-31/32 (Not exactly sure which one as NASA lists all these expedition as associated with study). Paper mentions Soyuz (not exactly sure which specific Soyuz mission). Mentions a 191 flight day mission | 1 | ISS/Soyuz |
| VANCOMYCIN | Resilient microorganisms in dust samples of the International Space Station-survival of the adaptation specialists | 27998314 | bacterial infection | post-flight | 8-12 years old dust samples | 1 | Russian ISS Modules |

*Continued on next page*

Table S1 – *Continued from previous page*

| Drug Name | Publication | PMID | Condition | Timing | Mission | Crew | Location |
|---|---|---|---|---|---|---|---|
| VANCOMYCIN | Cultivation in Space Flight Produces Minimal Alterations in the Susceptibility of Bacillus subtilis Cells to 72 Different Antibiotics and Growth-Inhibiting Compounds | 28821547 | bacterial infection | post-flight | Bacillus subtilis | 1 | ISS |
| VENLAFAXINE | Where Failure is not an Option - Personalized Medicine in Astronauts | 26489089 | depression | in-flight | Not one specific mission, but an accumulation of drugs aboard the ISS (as of 2014) | Unclear; not referenced specifically | ISS |
| VITAMIN D | The NASA Twins Study: A Multidimensional Analysis of a Year-Long Human Spaceflight | 30975860 | mineral supplementation | in-flight | Twin Study (though this is regularly provided to astronauts) | 1 | ISS |
| WATER / SALT | Water and Electrolyte Studies During Long-Term Missions Onboard the Space Stations SALYUT and MIR | 8012159 | space fluid / salt deconditionings | in-flight | Missions aboard Salyut and Mir (not specified which missions) | Unspecified number | Salyut and Mir |
| ZALEPLON | Medication Use by US Crewmembers on the International Space Station | 26187345 | sleep irregularities / slam shift | in-flight | Various missions from 2002-2012 (mix of short and long duration spaceflight) | 24 | ISS |
| ZALEPLON | Prevalence of Sleep Deficiency and use of hypnotic drugs in astronauts before, during, and after spaceflight: an observational study | 25127232 | irregular sleep | in-flight | Various space shuttle missions | 61 of the 78 crewmembers | Space shuttles |
| ZOLPIDEM | Prevalence of sleep deficiency and use of hypnotic drugs in astronauts before, during, and after spaceflight: an observational study | 25127232 | spaceflight induces sleep deficiency; hypnotic drugs provide only marginal benefit | both | 80 shuttle crew members and 21 ISS crew members in missions from 2001-2011 | 101 | Shuttles + ISS |
| ZOLPIDEM | Temazepam, but not zolpidem, causes orthostatic hypotension in astronauts after spaceflight | 12500019 | orthostatic hypotension | in-flight ; night before landing | 7-year period from January 1990 until December 1996 | 57 | space exposure |
| ZOLPIDEM | Medication Use by US Crewmembers on the International Space Station | 26187345 | sleep irregularities / slam shift | in-flight | Various missions from 2002-2012 (mix of short and long duration spaceflight) | 24 | ISS |
| ZOLPIDEM | Pharmaceutical Use by US Astronauts on Space Shuttle Missions (Source: "Supplying a Pharmacy for NASA Exploration Spaceflight: Challenges and Current Understanding") | 10417009 (31231-676) | insomnia | in-flight | 79 space shuttle missions | 219 | Space shuttles |
| ZOLPIDEM | Prevalence of Sleep Deficiency and use of hypnotic drugs in astronauts before, during, and after spaceflight: an observational study | 25127232 | irregular sleep | in-flight | Various space shuttle missions | 61 of the 78 crewmembers | Space shuttles |
| ZOLPIDEM | Prevalence of Sleep Deficiency and use of hypnotic drugs in astronauts before, during, and after spaceflight: an observational study | 25127232 | irregular sleep | in-flight | Various space shuttle missions | 61 of the 78 crewmembers | Space shuttles |
| ZOLPIDEM | Chemical Potency and Degradation Products of Medications Stored Over 550 Earth Days at the International Space Station | 26546565 | insomnia | tested post-flight | Sent via Russian Progress vehicle; returned via SpaceX Dragon Capsule | N/A (drugs were not administered to patients, but tested for efficacy) | ISS / ground |
| ZOLPIDEM | Sleep, performance, circadian rhythms, and light-dark cycles during two space shuttle flights | 11641138 | Sleep Disturbance | in-flight | STS 90/95 | 1 | STS 90/95 Space Shuttle |
| ZOLPIDEM; MELATONIN | Prevalence of Sleep Deficiency and use of hypnotic drugs in astronauts before, during, and after spaceflight: an observational study | 25127232 | irregular sleep | in-flight | Various space shuttle missions | 61 of the 78 crewmembers | Space shuttles |
| ZOLPIDEM; MELATONIN | Prevalence of Sleep Deficiency and use of hypnotic drugs in astronauts before, during, and after spaceflight: an observational study | 25127232 | irregular sleep | in-flight | Various space shuttle missions | 61 of the 78 crewmembers | Space shuttles |
| ZOLPIDEM; ZALEPLON | Prevalence of Sleep Deficiency and use of hypnotic drugs in astronauts before, during, and after spaceflight: an observational study | 25127232 | irregular sleep | in-flight | Various space shuttle missions | 61 of the 78 crewmembers | Space shuttles |

*Continued on next page*

Table S1 – *Continued from previous page*

| Drug Name | Publication | PMID | Condition | Timing | Mission | Crew | Location | |
|---|---|---|---|---|---|---|---|---|
| ZOLPIDEM; ZALEPLON | Prevalence of Sleep Deficiency and use of hypnotic drugs in astronauts before, during, and after spaceflight: an observational study | 25127232 | irregular sleep | in-flight | Various space shuttle missions | 61 of the 78 crewmembers | Space shuttles | |
| | | | | | | | | |
| NO STANDARD NAME | | | | | | | | |
| | Potential Venous Thromboembolism Risk in Female Astronauts | 32327017 | venous thromboembolism ; never observed during spaceflight | in-flight | short and long duration missions between 2000 and 2014 | 38 | Shuttles + ISS | |
| | Albumin, oral contraceptives, and venous thromboembolism risk in astronauts | 35389755 | venous thromboembolism occured ; lower concentrations of serum albumin and higher concentrations of transferrin | in-flight | long-duration spaceflight on the International Space Station | 65 | ISS | |
| | Chapter 4 Pharmacology in Space: Pharmacotherapy | 9048135 | prevents disruption of vitamin and mineral metabolism | in-flight | 166-day MIR mission | 2 | MIR Space Station | |
| | Chapter 4 Pharmacology in Space: Pharmacotherapy | 9048135 | space motion sickness | in-flight | Skylab-3 and 4 | 6 | Skylab Space Station | |
| | Impact of 60 days of 6˜extdegree head down bed rest on cardiopulmonary function, and the effects of Taikong Yangxin Prescription as a countermeasure | 24810476 | cardiopulmonary function | 6˜extdegree head down bed rest (HDBR) | | 14 | 6˜extdegree head down bed rest (HDBR) | |
| | Medication Use by US Crewmembers on the International Space Station | 26187345 | pain (back, joint, muscle, and headaches) | in-flight | Various missions from 2002-2012 (mix of short and long duration spaceflight) | 24 | ISS | |
| | Medication Use by US Crewmembers on the International Space Station | 26187345 | rashes | in-flight | Various missions from 2002-2012 (mix of short and long duration spaceflight) | 24 | ISS | |
| | Medication Use by US Crewmembers on the International Space Station | 26187345 | rashes | in-flight | Various missions from 2002-2012 (mix of short and long duration spaceflight) | 24 | ISS | |
| | Medication Use by US Crewmembers on the International Space Station | 26187345 | rashes | in-flight | Various missions from 2002-2012 (mix of short and long duration spaceflight) | 24 | ISS | |
| | Medication Use by US Crewmembers on the International Space Station | 26187345 | rashes | in-flight | Various missions from 2002-2012 (mix of short and long duration spaceflight) | 24 | ISS | |
| | Medication Use by US Crewmembers on the International Space Station | 26187345 | SAS (space adaptation syndrome) | in-flight | Various missions from 2002-2012 (mix of short and long duration spaceflight) | 24 | ISS | |
| | Medication Use by US Crewmembers on the International Space Station | 26187345 | eye inflammation; urinary tract infection; upper respiratory infection | in-flight | Various missions from 2002-2012 (mix of short and long duration spaceflight) | 24 | ISS | |
| | Multi-resistant biofilm-forming pathogens on the International Space Station | 31719234 | bacterial infection | in-flight | filter samples | 1 | ISS | |
| | Multi-resistant biofilm-forming pathogens on the International Space Station | 31719234 | bacterial infection | in-flight | filter samples | 1 | ISS | |
| | Multi-resistant biofilm-forming pathogens on the International Space Station | 31719234 | bacterial infection | in-flight | filter samples | 1 | ISS | |
| | Multi-resistant biofilm-forming pathogens on the International Space Station | 31719234 | bacterial infection | in-flight | filter samples | 1 | ISS | |

# SUPPLEMENTARY TABLE S1B: CATALOG OF EXPERIMENTAL REAGENTS AND ANIMAL STUDIES

Table S2: **Experimental Reagents.**

| Drug Name | Publication | PMID | Condition | Timing | Mission | Crew | Location |
|---|---|---|---|---|---|---|---|
| ACETAMINOPHEN | Simulated Microgravity Alters P-Glycoprotein Efflux Function and Expression via the Wnt/ | | | | | | |
| -beta$-Catenin Signaling Pathway in Rat Intestine and Brain$ | 36982513 | oral drug absorption and distribution | simulated microgravity | | n/a (not crew members, but rat model) | simulated microgravity | |
| ANTIPYRENE | Antipyrene Pharmacokinetics in the Tail-Suspended Rat Model | 7616419 | used on earth for ear infections and swelling; it is an anti-inflammatory drug | neither; tail-suspended rat model | N/A (tested on ground; simulated weightlessness) | N/A (not crew members, but rat model) | Ground study (simulated weightlessness) |
| CAFFEINE | Effects of spaceflight and ground recovery on mesenteric artery and vein constrictor properties in mice | 23099650 | orthostatic hypotension | post-landing on arteries | (STS)-131 (n=11), STS-133 (n=6), and STS-135 (n=3) | n/a (not crew members, but rat model) | Space Transportation System |
| CAFFEINE | Calcium movement of sarcoplasmic reticulum from hindlimb suspended muscle | 11540780 | increased calcium leakage | hind limb unloading (HLU) rats' model | | n/a (not crew members, but rat model) | ground-study |
| CALYCOSIN | Calycosin prevents bone loss induced by hindlimb unloading | 35794112 | microgravity-induced bone loss | hind limb unloading (HLU) rats' model | | n/a (not crew members, but rat model) | ground-study |
| CHLORAL HYDRATE | Factors Affecting Drug Bioavailability in Space | 8083399 | insomnia | in-flight | First 33 space shuttle missions | A specific number is unclear, but 500 individual doses were administered over 83% of the crewmembers | 33 space shuttles |
| ENROFLOXACIN | Broad-spectrum antibiotic or G-CSF as potential countermeasures for impaired control of bacterial infection associated with an SPE exposure during spaceflight | 25793272 | Pseudomonas aeruginosa infection | hind limb unloading (HLU) mice model | | n/a (not crew members, but rat model) | ground-study |
| NOREPINEPHRINE | Effects of spaceflight and ground recovery on mesenteric artery and vein constrictor properties in mice | 23099650 | orthostatic hypotension | post-landing on arteries | (STS)-131 (n=11), STS-133 (n=6), and STS-135 (n=3) | n/a (not crew members, but rat model) | Space Transportation System |
| POTASSIUM CHLORIDE | Effects of spaceflight and ground recovery on mesenteric artery and vein constrictor properties in mice | 23099650 | orthostatic hypotension | post-landing on arteries | (STS)-131 (n=11), STS-133 (n=6), and STS-135 (n=3) | n/a (not crew members, but rat model) | Space Transportation System |
| POTASSIUM CITRATE | Effect of Potassium Citrate Therapy on the Risk of Renal Ston Formation During Spaceflight | 19765769 | Maintain levels of urinary calcium excretion to reduce renal stones | in-flight | ISS Expeditions 3-6, 8 and 11-13. | 30 | ISS and MIR space station |
| TEMAZEPAM | Temazepam, but Not Zolpidem, Causes Orthostatic Hypotensionin Astronauts After Spaceflight | 12500019 | Increase in heart rate | in-flight | various space missions from Jan 1990-Dec 1996 | 9 | |
| | Broad-spectrum antibiotic or G-CSF as potential countermeasures for impaired control of bacterial infection associated with an SPE exposure during spaceflight | 25793272 | Pseudomonas aeruginosa infection | hind limb unloading (HLU) mice model | | n/a (not crew members, but rat model) | ground-study |

# SUPPLEMENTARY TABLE S2: DRUG-GENE INTERACTIONS

Table S3: **Spaceflight-Flown Drug-Gene Interaction Database.**

| Gene | Drug Name | Score | Type/Mechanism | Approval |
|---|---|---|---|---|
| A2M | THROMBIN | NA | NA | NA |
| AANAT | MELATONIN | MELATO-NIN | MELATONIN | Definite |
| ABCA1 | ATORVASTATIN CALCIUM TRIHYDRATE | NA | NA | NA |
| ABCB1 | ACETAMINOPHEN | NA | NA | NA |
| ABCB1 | ARIPIPRAZOLE | ARIPIP-RAZOLE | ARIPIPRAZOLE | Definite |
| ABCB1 | ATORVASTATIN CALCIUM TRIHYDRATE | NA | NA | NA |
| ABCB1 | AZITHROMYCIN | NA | NA | NA |
| ABCB1 | BLEOMYCIN | BLEOMY-CIN | BLEOMYCIN | Ambiguous |
| ABCB1 | CLARITHROMYCIN | CLARIT-HROMYC-IN | CLARITHROMYCIN | Definite |
| ABCB1 | CODEINE | CODEINE | CODEINE | Definite |
| ABCB1 | CODEINE ANHYDROUS | NA | NA | NA |
| ABCB1 | DEXAMETHASONE | DEXAME-THASONE | DEXAMETHASONE | Definite |
| ABCB1 | DOXYCYCLINE | DOXYCY-CLINE | DOXYCYCLINE | Ambiguous |
| ABCB1 | DOXYCYCLINE ANHYDROUS | NA | NA | NA |
| ABCB1 | ERYTHROMYCIN | ERYTHR-OMYCIN | ERYTHROMYCIN | Definite |
| ABCB1 | FLUOROURACIL | NA | NA | NA |
| ABCB1 | HYDROCORTISONE | HYDROC-ORTISONE | HYDROCORTISONE | Definite |
| ABCB1 | HYDROCORTISONE BUTYRA-TE | NA | NA | NA |
| ABCB1 | IBUPROFEN | IBUPRO-FEN | IBUPROFEN | Definite |
| ABCB1 | IBUPROFEN, SODIUM SALT | NA | NA | NA |
| ABCB1 | LEVOFLOXACIN | LEVOFL-OXACIN | LEVOFLOXACIN | Definite |
| ABCB1 | LEVOFLOXACIN ANHYDROUS | NA | NA | NA |
| ABCB1 | LOPERAMIDE | LOPERA-MIDE | LOPERAMIDE | Definite |
| ABCB1 | METHYLPREDNISOLONE | METHYL-PREDNI-SOLONE | METHYLPREDNISOLONE | Definite |
| ABCB1 | MODAFINIL | MODAFI-NIL | MODAFINIL | Definite |
| ABCB1 | OMEPRAZOLE | OMEPRA-ZOLE | OMEPRAZOLE | Definite |
| ABCB1 | ONDANSETRON | ONDANS-ETRON | ONDANSETRON | Definite |
| ABCB1 | ONDANSETRON HYDROCHLO-RIDE | NA | NA | NA |
| ABCB1 | PROPOFOL | PROPOF-OL | PROPOFOL | Definite |
| ABCB1 | RIFAMPIN | RIFAMPIN | RIFAMPIN | Definite |
| ABCB1 | SERTRALINE | SERTRA-LINE | SERTRALINE | Definite |
| ABCB1 | TETRACYCLINE | TETRAC-YCLINE | TETRACYCLINE | Definite |
| ABCB1 | VENLAFAXINE | VENLAF-AXINE | VENLAFAXINE | Definite |
| ABCB1 | VITAMIN B12 | NA | NA | NA |
| ABCB1 | VITAMIN E | NA | NA | NA |
| ABCC1 | AZITHROMYCIN | AZITHR-OMYCIN | AZITHROMYCIN | Definite |
| ABCC1 | ERYTHROMYCIN | ERYTHR-OMYCIN | ERYTHROMYCIN | Definite |
| ABCC1 | NOVOBIOCIN | NOVOBI-OCIN | NOVOBIOCIN | Definite |
| ABCC1 | OFLOXACIN | OFLOXA-CIN | OFLOXACIN | Definite |
| ABCC11 | FLUOROURACIL | NA | NA | NA |
| ABCC2 | CEFOXITIN | CEFOXI-TIN | CEFOXITIN | Definite |
| ABCC2 | CEFOXITIN SODIUM | NA | NA | NA |

*Continued on next page*

Table S3 – *Continued from previous page*

| Gene | Drug Name | Score | Type/Mechanism | Approval |
|---|---|---|---|---|
| ABCC2 | CEFTRIAXONE | CEFTRI-AXONE | CEFTRIAXONE | Definite |
| ABCC2 | CEFUROXIME | CEFURO-XIME | CEFUROXIME | Definite |
| ABCC2 | CEFUROXIME AXETIL | NA | NA | NA |
| ABCC2 | CEPHALEXIN | CEPHAL-EXIN | CEPHALEXIN | Definite |
| ABCC2 | CEPHALEXIN ANHYDROUS | NA | NA | NA |
| ABCC2 | ERYTHROMYCIN | ERYTHR-OMYCIN | ERYTHROMYCIN | Definite |
| ABCC2 | FLUOROURACIL | NA | NA | NA |
| ABCC3 | CEFOXITIN | CEFOXI-TIN | CEFOXITIN | Definite |
| ABCC3 | CEFOXITIN SODIUM | NA | NA | NA |
| ABCC3 | CEFTRIAXONE | CEFTRI-AXONE | CEFTRIAXONE | Definite |
| ABCC3 | CEFUROXIME | CEFURO-XIME | CEFUROXIME | Definite |
| ABCC3 | CEFUROXIME AXETIL | NA | NA | NA |
| ABCC3 | CEPHALEXIN | CEPHAL-EXIN | CEPHALEXIN | Definite |
| ABCC3 | CEPHALEXIN ANHYDROUS | NA | NA | NA |
| ABCC4 | CEFOXITIN | CEFOXI-TIN | CEFOXITIN | Definite |
| ABCC4 | CEFOXITIN SODIUM | NA | NA | NA |
| ABCC4 | CEFTRIAXONE | CEFTRI-AXONE | CEFTRIAXONE | Definite |
| ABCC4 | CEFUROXIME | CEFURO-XIME | CEFUROXIME | Definite |
| ABCC4 | CEFUROXIME AXETIL | NA | NA | NA |
| ABCC4 | CEPHALEXIN | CEPHAL-EXIN | CEPHALEXIN | Definite |
| ABCC4 | CEPHALEXIN ANHYDROUS | NA | NA | NA |
| ABCC4 | FLUOROURACIL | NA | NA | NA |
| ABCC4 | FUROSEMIDE | FUROSE-MIDE | FUROSEMIDE | Definite |
| ABCC5 | FLUOROURACIL | NA | NA | NA |
| ABCC6 | SODIUM CHLORIDE | SODIUM CHLORIDE | SODIUM CHLORIDE | Definite |
| ABCG1 | FLUOROURACIL | NA | NA | NA |
| ABCG2 | APIXABAN | APIXABAN | APIXABAN | Definite |
| ABCG2 | CEFTRIAXONE | CEFTRI-AXONE | CEFTRIAXONE | Definite |
| ABCG2 | LEVOFLOXACIN ANHYDROUS | NA | NA | NA |
| ABCG2 | NITROFURANTOIN | NITROF-URANTOIN | NITROFURANTOIN | Definite |
| ABCG2 | NITROFURANTOIN, MACROC-RYSTALS | NA | NA | NA |
| ABL1 | ACYCLOVIR | NA | NA | NA |
| ACE | ASPIRIN | NA | NA | NA |
| ACE | ATORVASTATIN CALCIUM TRIHYDRATE | NA | NA | NA |
| ACE | FUROSEMIDE | FUROSE-MIDE | FUROSEMIDE | Definite |
| ACE | METOPROLOL | METOPR-OLOL | METOPROLOL | Definite |
| ACE | SERTRALINE | SERTRA-LINE | SERTRALINE | Definite |
| ACTC1 | DEXAMETHASONE | DEXAME-THASONE | DEXAMETHASONE | Definite |
| ADA | HYDROCORTISONE | HYDROC-ORTISONE | HYDROCORTISONE | Definite |
| ADA | HYDROCORTISONE BUTYRA-TE | NA | NA | NA |
| ADAMTSL4--AS1 | ASPIRIN | NA | NA | NA |
| ADAMTSL4--AS1 | OMEPRAZOLE | NA | NA | NA |
| ADAMTSL4--AS1 | PROCHLORPERAZINE | NA | NA | NA |
| ADCYAP1R1 | NALOXONE HYDROCHLORIDE | NA | NA | NA |
| ADD1 | FUROSEMIDE | FUROSE-MIDE | FUROSEMIDE | Definite |
| ADK | BLEOMYCIN | BLEOMY-CIN | BLEOMYCIN | Ambiguous |
| ADORA1 | ASPIRIN | NA | NA | NA |

*Continued on next page*

Table S3 – *Continued from previous page*

| Gene | Drug Name | Score | Type/Mechanism | Approval | |
|---|---|---|---|---|---|
| ADORA1 | CAFFEINE | CAFFEINE | CAFFEINE | Definite | |
| ADORA2A | CAFFEINE | CAFFEINE | CAFFEINE | Definite | |
| ADORA2B | CAFFEINE | CAFFEINE | CAFFEINE | Definite | |
| ADORA3 | CAFFEINE | CAFFEINE | CAFFEINE | Definite | |
| ADRA1A | DEXTROAMPHETAMINE SULFATE | NA | NA | NA | |
| ADRA1A | EPINEPHRINE | EPINEP-HRINE | EPINEPHRINE | Definite | |
| ADRA1A | NOREPINEPHRINE | NOREPI-NEPHRINE | NOREPINEPHRINE | Definite | |
| ADRA1A | OXYMETAZOLINE | OXYMET-AZOLINE | OXYMETAZOLINE | Definite | |
| ADRA1A | OXYMETAZOLINE HYDROC-HLORIDE | NA | NA | NA | |
| ADRA1A | PHENYLEPHRINE | PHENYL-EPHRINE | PHENYLEPHRINE | Definite | |
| ADRA1A | PHENYLPROPANOLAMINE | PHENYL-PROPAN-OLAMINE | PHENYLPROPANOLAMINE | Definite | |
| ADRA1A | PSEUDOEPHEDRINE | PSEUDO-EPHEDR-INE | PSEUDOEPHEDRINE | Definite | |
| ADRA1A | TAMSULOSIN | TAMSUL-OSIN | TAMSULOSIN | Definite | |
| ADRA1A | TAMSULOSIN HYDROCHLO-RIDE | NA | NA | NA | |
| ADRA1A | TRIFLUOPERAZINE | TRIFLU-OPERAZ-INE | TRIFLUOPERAZINE | Definite | |
| ADRA1B | EPINEPHRINE | EPINEP-HRINE | EPINEPHRINE | Definite | |
| ADRA1B | NOREPINEPHRINE | NOREPI-NEPHRINE | NOREPINEPHRINE | Definite | |
| ADRA1B | OXYMETAZOLINE HYDROC-HLORIDE | NA | NA | NA | |
| ADRA1B | PHENYLEPHRINE | PHENYL-EPHRINE | PHENYLEPHRINE | Definite | |
| ADRA1B | QUETIAPINE FUMARATE | NA | NA | NA | |
| ADRA1B | TAMSULOSIN | TAMSUL-OSIN | TAMSULOSIN | Definite | |
| ADRA1B | TAMSULOSIN HYDROCHLO-RIDE | NA | NA | NA | |
| ADRA1D | EPINEPHRINE | EPINEP-HRINE | EPINEPHRINE | Definite | |
| ADRA1D | METOPROLOL SUCCINATE | NA | NA | NA | |
| ADRA1D | MODAFINIL | NA | NA | NA | |
| ADRA1D | NOREPINEPHRINE | NOREPI-NEPHRINE | NOREPINEPHRINE | Definite | |
| ADRA1D | OXYMETAZOLINE HYDROC-HLORIDE | NA | NA | NA | |
| ADRA1D | PHENYLEPHRINE | PHENYL-EPHRINE | PHENYLEPHRINE | Definite | |
| ADRA1D | TAMSULOSIN | TAMSUL-OSIN | TAMSULOSIN | Definite | |
| ADRA1D | TAMSULOSIN HYDROCHLO-RIDE | NA | NA | NA | |
| ADRA2A | DEXTROAMPHETAMINE SULFATE | NA | NA | NA | |
| ADRA2A | EPINEPHRINE | EPINEP-HRINE | EPINEPHRINE | Definite | |
| ADRA2A | NOREPINEPHRINE | NOREPI-NEPHRINE | NOREPINEPHRINE | Definite | |
| ADRA2A | OXYMETAZOLINE | OXYMET-AZOLINE | OXYMETAZOLINE | Definite | |
| ADRA2A | OXYMETAZOLINE HYDROC-HLORIDE | NA | NA | NA | |
| ADRA2A | PHENYLPROPANOLAMINE | PHENYL-PROPAN-OLAMINE | PHENYLPROPANOLAMINE | Definite | |
| ADRA2A | PSEUDOEPHEDRINE | PSEUDO-EPHEDR-INE | PSEUDOEPHEDRINE | Definite | |
| ADRA2A | SODIUM CHLORIDE | NA | NA | NA | |
| ADRA2B | EPINEPHRINE | EPINEP-HRINE | EPINEPHRINE | Definite | |
| ADRA2B | NOREPINEPHRINE | NOREPI-NEPHRINE | NOREPINEPHRINE | Definite | |

*Continued on next page*

Table S3 – *Continued from previous page*

| Gene | Drug Name | Score | Type/Mechanism | Approval |
|---|---|---|---|---|
| ADRA2B | OXYMETAZOLINE HYDROC-HLORIDE | NA | NA | NA |
| ADRA2C | EPINEPHRINE | EPINEP-HRINE | EPINEPHRINE | Definite |
| ADRA2C | METOPROLOL | NA | NA | NA |
| ADRA2C | NOREPINEPHRINE | NOREPI-NEPHRINE | NOREPINEPHRINE | Definite |
| ADRA2C | OXYMETAZOLINE HYDROC-HLORIDE | NA | NA | NA |
| ADRB1 | EPINEPHRINE | EPINEP-HRINE | EPINEPHRINE | Definite |
| ADRB1 | METOPROLOL | METOPR-OLOL | METOPROLOL | Definite |
| ADRB1 | METOPROLOL SUCCINATE | METOPR-OLOL SUCCIN-ATE | METOPROLOL SUCCINATE | Definite |
| ADRB1 | NOREPINEPHRINE | NOREPI-NEPHRINE | NOREPINEPHRINE | Definite |
| ADRB2 | CLOTRIMAZOLE | CLOTRI-MAZOLE | CLOTRIMAZOLE | Definite |
| ADRB2 | EPINEPHRINE | EPINEP-HRINE | EPINEPHRINE | Definite |
| ADRB2 | METOPROLOL | METOPR-OLOL | METOPROLOL | Definite |
| ADRB2 | NOREPINEPHRINE | NOREPI-NEPHRINE | NOREPINEPHRINE | Definite |
| ADRB2 | PHENYLEPHRINE | PHENYL-EPHRINE | PHENYLEPHRINE | Definite |
| ADRB2 | PROPOFOL | PROPOF-OL | PROPOFOL | Definite |
| ADRB2 | PSEUDOEPHEDRINE | PSEUDO-EPHEDR-INE | PSEUDOEPHEDRINE | Definite |
| ADRB2 | SODIUM CHLORIDE | NA | NA | NA |
| ADRB3 | EPINEPHRINE | EPINEP-HRINE | EPINEPHRINE | Definite |
| AFP | ASPIRIN | NA | NA | NA |
| AFP | BLEOMYCIN | BLEOMY-CIN | BLEOMYCIN | Ambiguous |
| AGBL4 | RIFAMPIN | RIFAMPIN | RIFAMPIN | Definite |
| AGER | VITAMIN B12 | NA | NA | NA |
| AGT | ASPIRIN | NA | NA | NA |
| AGT | HYDROCORTISONE | HYDROC-ORTISONE | HYDROCORTISONE | Definite |
| AGT | HYDROCORTISONE BUTYRA-TE | NA | NA | NA |
| AGTR1 | ASCORBIC ACID | NA | NA | NA |
| AGTR1 | ATORVASTATIN CALCIUM TRIHYDRATE | NA | NA | NA |
| AGTR1 | DEXAMETHASONE | DEXAME-THASONE | DEXAMETHASONE | Definite |
| AGTR1 | SODIUM CHLORIDE | NA | NA | NA |
| AHR | LEVOTHYROXINE | LEVOTH-YROXINE | LEVOTHYROXINE | Definite |
| AHR | OMEPRAZOLE | OMEPRA-ZOLE | OMEPRAZOLE | Definite |
| AHR | PHENAZOPYRIDINE HYDROC-HLORIDE | NA | NA | NA |
| AKT1 | TRIFLUOPERAZINE | TRIFLU-OPERAZ-INE | TRIFLUOPERAZINE | Definite |
| ALB | DIAZEPAM | DIAZEPAM | DIAZEPAM | Definite |
| ALB | FLUCONAZOLE | NA | NA | NA |
| ALCAM | FLUOROURACIL | NA | NA | NA |
| ALDH1A1 | CHLORAMPHENICOL | CHLORA-MPHENI-COL | CHLORAMPHENICOL | Definite |
| ALDH1A1 | MERCAPTOPURINE | NA | NA | NA |
| ALDH1A1 | METRONIDAZOLE | METRON-IDAZOLE | METRONIDAZOLE | Definite |
| ALDH3A1 | FLUOROURACIL | NA | NA | NA |
| ALK | DEXAMETHASONE | NA | NA | NA |
| ALOX15 | PSEUDOEPHEDRINE | PSEUDO-EPHEDR-INE | PSEUDOEPHEDRINE | Definite |

*Continued on next page*

Table S3 – *Continued from previous page*

| Gene | Drug Name | Score | Type/Mechanism | Approval |
|---|---|---|---|---|
| ALOX15 | SILVER SULFADIAZINE | SILVER SULFAD-IAZINE | SILVER SULFADIAZINE | Definite |
| ALOX15B | PSEUDOEPHEDRINE | PSEUDO-EPHEDR-INE | PSEUDOEPHEDRINE | Definite |
| ALOX5 | MELATONIN | MELATO-NIN | MELATONIN | Definite |
| AMACR | IBUPROFEN | IBUPRO-FEN | IBUPROFEN | Definite |
| AMACR | IBUPROFEN, SODIUM SALT | NA | NA | NA |
| ANKK1 | ARIPIPRAZOLE | ARIPIP-RAZOLE | ARIPIPRAZOLE | Definite |
| ANKK1 | PROCHLORPERAZINE | PROCHL-ORPERA-ZINE | PROCHLORPERAZINE | Definite |
| ANKK1 | QUETIAPINE FUMARATE | NA | NA | NA |
| ANPEP | GENTAMICIN | GENTAM-ICIN | GENTAMICIN | Definite |
| ANPEP | NALOXONE HYDROCHLORIDE | NA | NA | NA |
| ANXA1 | DEXAMETHASONE | DEXAME-THASONE | DEXAMETHASONE | Definite |
| ANXA1 | HYDROCORTISONE | HYDROC-ORTISONE | HYDROCORTISONE | Definite |
| ANXA1 | HYDROCORTISONE BUTYRA-TE | NA | NA | NA |
| ANXA1 | METHYLPREDNISOLONE | METHYL-PREDNI-SOLONE | METHYLPREDNISOLONE | Definite |
| ANXA1 | TRIAMCINOLONE | TRIAMC-INOLONE | TRIAMCINOLONE | Definite |
| APAF1 | BENZETHONIUM CHLORIDE | BENZET-HONIUM CHLORIDE | BENZETHONIUM CHLORIDE | Definite |
| APC | IBUPROFEN | IBUPRO-FEN | IBUPROFEN | Definite |
| APC | IBUPROFEN, SODIUM SALT | NA | NA | NA |
| APEX1 | AMPICILLIN | AMPICI-LLIN | AMPICILLIN | Definite |
| APEX1 | AMPICILLIN (ANHYDROUS) | NA | NA | NA |
| APEX1 | CEFOTAXIME SODIUM | NA | NA | NA |
| APEX1 | EPINEPHRINE | EPINEP-HRINE | EPINEPHRINE | Definite |
| APEX1 | FLUOROURACIL | NA | NA | NA |
| APEX1 | NOREPINEPHRINE | NOREPI-NEPHRINE | NOREPINEPHRINE | Definite |
| APOA1 | FUROSEMIDE | FUROSE-MIDE | FUROSEMIDE | Definite |
| APOA1 | PROGESTIN | NA | NA | NA |
| APOA5 | ATORVASTATIN CALCIUM TRIHYDRATE | NA | NA | NA |
| APOB | ATORVASTATIN CALCIUM TRIHYDRATE | NA | NA | NA |
| APOB | DEXAMETHASONE | DEXAME-THASONE | DEXAMETHASONE | Definite |
| APOB | HYDROCORTISONE | HYDROC-ORTISONE | HYDROCORTISONE | Definite |
| APOB | HYDROCORTISONE BUTYRA-TE | NA | NA | NA |
| APOB | TRIFLUOPERAZINE | TRIFLU-OPERAZ-INE | TRIFLUOPERAZINE | Definite |
| APOB | VITAMIN E | VITAMIN E | VITAMIN E | Ambiguous |
| APOE | TRIAMCINOLONE | TRIAMC-INOLONE | TRIAMCINOLONE | Definite |
| APOE | VITAMIN E | NA | NA | NA |
| APP | PROPOFOL | PROPOF-OL | PROPOFOL | Definite |
| APRT | HYDROGEN PEROXIDE | NA | NA | NA |
| AQP1 | ASCORBIC ACID | NA | NA | NA |
| AQP4 | PHENYTOIN | PHENYT-OIN | PHENYTOIN | Definite |
| AQP4 | PHENYTOIN SODIUM | NA | NA | NA |
| AR | BENZETHONIUM CHLORIDE | BENZET-HONIUM CHLORIDE | BENZETHONIUM CHLORIDE | Definite |
| AR | BISACODYL | BISACO-DYL | BISACODYL | Definite |

*Continued on next page*

Table S3 – *Continued from previous page*

| Gene | Drug Name | Score | Type/Mechanism | Approval |
|---|---|---|---|---|
| AR | CAFFEINE | CAFFEINE | CAFFEINE | Definite |
| AR | CLOTRIMAZOLE | CLOTRI-MAZOLE | CLOTRIMAZOLE | Definite |
| AR | CODEINE | CODEINE | CODEINE | Definite |
| AR | CODEINE ANHYDROUS | NA | NA | NA |
| AR | DEQUALINIUM CHLORIDE | DEQUAL-INIUM CHLORIDE | DEQUALINIUM CHLORIDE | Definite |
| AR | DEXAMETHASONE | DEXAME-THASONE | DEXAMETHASONE | Definite |
| AR | DIAZEPAM | DIAZEPAM | DIAZEPAM | Definite |
| AR | HYDROCORTISONE | HYDROC-ORTISONE | HYDROCORTISONE | Definite |
| AR | HYDROCORTISONE BUTYRA-TE | NA | NA | NA |
| AR | LOPERAMIDE | LOPERA-MIDE | LOPERAMIDE | Definite |
| AR | LORATADINE | LORATA-DINE | LORATADINE | Definite |
| AR | METHYLPREDNISOLONE | METHYL-PREDNI-SOLONE | METHYLPREDNISOLONE | Definite |
| AR | PROMETHAZINE HYDROC-HLORIDE | PROMET-HAZINE HYDROC-HLORIDE | PROMETHAZINE HYDROCHLORIDE | Definite |
| AR | RIFAMPIN | RIFAMPIN | RIFAMPIN | Definite |
| AR | SERTRALINE HYDROCHLO-RIDE | NA | NA | NA |
| AR | TAMSULOSIN HYDROCHLO-RIDE | NA | NA | NA |
| AR | TRIAMCINOLONE | TRIAMC-INOLONE | TRIAMCINOLONE | Definite |
| AR | TRIAMCINOLONE ACETONIDE | TRIAMC-INOLONE ACETON-IDE | TRIAMCINOLONE ACETONIDE | Definite |
| AR | ZOLPIDEM | ZOLPIDEM | ZOLPIDEM | Definite |
| AR | ZOLPIDEM TARTRATE | NA | NA | NA |
| ARNT | HYDROCORTISONE | HYDROC-ORTISONE | HYDROCORTISONE | Definite |
| ARNT | HYDROCORTISONE BUTYRA-TE | NA | NA | NA |
| ARSB | ASCORBIC ACID | NA | NA | NA |
| ATAD5 | CALYCOSIN | CALYCO-SIN | CALYCOSIN | Definite |
| ATF4 | VITAMIN E | NA | NA | NA |
| ATG4B | DOXYCYCLINE ANHYDROUS | NA | NA | NA |
| ATM | FLUOROURACIL | NA | NA | NA |
| ATP4A | OMEPRAZOLE | OMEPRA-ZOLE | OMEPRAZOLE | Definite |
| ATP4B | OMEPRAZOLE | OMEPRA-ZOLE | OMEPRAZOLE | Definite |
| ATRX | FLUOROURACIL | NA | NA | NA |
| ATXN2 | BENZETHONIUM CHLORIDE | BENZET-HONIUM CHLORIDE | BENZETHONIUM CHLORIDE | Definite |
| ATXN2 | CLOTRIMAZOLE | CLOTRI-MAZOLE | CLOTRIMAZOLE | Definite |
| ATXN2 | PROCHLORPERAZINE | PROCHL-ORPERA-ZINE | PROCHLORPERAZINE | Definite |
| ATXN2 | PROMETHAZINE | PROMET-HAZINE | PROMETHAZINE | Definite |
| ATXN2 | PROMETHAZINE HYDROC-HLORIDE | NA | NA | NA |
| ATXN2 | SERTRALINE | SERTRA-LINE | SERTRALINE | Definite |
| ATXN2 | SERTRALINE HYDROCHLO-RIDE | NA | NA | NA |
| ATXN2 | TRIFLUOPERAZINE | TRIFLU-OPERAZ-INE | TRIFLUOPERAZINE | Definite |
| AURKA | FLUOROURACIL | NA | NA | NA |
| AVPR2 | CLOTRIMAZOLE | CLOTRI-MAZOLE | CLOTRIMAZOLE | Definite |
| B2M | AMIKACIN | AMIKACIN | AMIKACIN | Definite |
| B4GALT2 | ASPIRIN | NA | NA | NA |

*Continued on next page*

Table S3 – *Continued from previous page*

| Gene | Drug Name | Score | Type/Mechanism | Approval | |
|---|---|---|---|---|---|
| BAX | CIPROFLOXACIN | CIPROF-LOXACIN | CIPROFLOXACIN | Definite | |
| BAX | ERYTHROMYCIN | ERYTHR-OMYCIN | ERYTHROMYCIN | Definite | |
| BAX | FLUOROURACIL | NA | NA | NA | |
| BAX | MELATONIN | MELATO-NIN | MELATONIN | Definite | |
| BAX | PHENYLEPHRINE | PHENYL-EPHRINE | PHENYLEPHRINE | Definite | |
| BAX | SODIUM CHLORIDE | SODIUM CHLORIDE | SODIUM CHLORIDE | Definite | |
| BAZ2B | CEFOXITIN SODIUM | NA | NA | NA | |
| BAZ2B | CHLORTETRACYCLINE HYD-ROCHLORIDE | CHLORT-ETRACY-CLINE HYDROC-HLORIDE | CHLORTETRACYCLINE HYDROCHLORIDE | Definite | |
| BAZ2B | CLOTRIMAZOLE | CLOTRI-MAZOLE | CLOTRIMAZOLE | Definite | |
| BAZ2B | DOXYCYCLINE ANHYDROUS | NA | NA | NA | |
| BAZ2B | MERCAPTOPURINE | NA | NA | NA | |
| BAZ2B | NALIDIXIC ACID | NALIDIXIC ACID | NALIDIXIC ACID | Definite | |
| BAZ2B | OMEPRAZOLE | OMEPRA-ZOLE | OMEPRAZOLE | Definite | |
| BAZ2B | PHTHALYLSULFATHIAZOLE | PHTHAL-YLSULF-ATHIAZ-OLE | PHTHALYLSULFATHIAZOLE | Definite | |
| BAZ2B | PROCHLORPERAZINE | PROCHL-ORPERA-ZINE | PROCHLORPERAZINE | Definite | |
| BAZ2B | PROMETHAZINE HYDROC-HLORIDE | PROMET-HAZINE HYDROC-HLORIDE | PROMETHAZINE HYDROCHLORIDE | Definite | |
| BAZ2B | TRIAZOLAM | TRIAZO-LAM | TRIAZOLAM | Definite | |
| BCHE | TRIAMCINOLONE | TRIAMC-INOLONE | TRIAMCINOLONE | Definite | |
| BCL2 | ERYTHROMYCIN | ERYTHR-OMYCIN | ERYTHROMYCIN | Definite | |
| BCL2 | METHYLPREDNISOLONE | METHYL-PREDNI-SOLONE | METHYLPREDNISOLONE | Definite | |
| BCL2 | VITAMIN E | VITAMIN E | VITAMIN E | Ambiguous | |
| BDKRB2 | ATORVASTATIN CALCIUM TRIHYDRATE | NA | NA | NA | |
| BDKRB2 | SODIUM CHLORIDE | NA | NA | NA | |
| BDNF | FLUOROURACIL | NA | NA | NA | |
| BDNF | GENTAMICIN | GENTAM-ICIN | GENTAMICIN | Definite | |
| BDNF | LIDOCAINE | LIDOCA-INE | LIDOCAINE | Definite | |
| BDNF | METHYLPREDNISOLONE | METHYL-PREDNI-SOLONE | METHYLPREDNISOLONE | Definite | |
| BDNF | NALOXONE HYDROCHLORIDE | NA | NA | NA | |
| BDNF | SODIUM CHLORIDE | SODIUM CHLORIDE | SODIUM CHLORIDE | Definite | |
| BDNF | VENLAFAXINE | VENLAF-AXINE | VENLAFAXINE | Definite | |
| BGLAP | ATORVASTATIN CALCIUM TRIHYDRATE | NA | NA | NA | |
| BGLAP | MELATONIN | MELATO-NIN | MELATONIN | Definite | |
| BGLAP | VITAMIN D | NA | NA | NA | |
| BIRC5 | DEXAMETHASONE | DEXAME-THASONE | DEXAMETHASONE | Definite | |
| BIRC5 | FLUOROURACIL | NA | NA | NA | |
| BLM | NOREPINEPHRINE | NOREPI-NEPHRINE | NOREPINEPHRINE | Definite | |
| BLMH | BLEOMYCIN | NA | NA | NA | |
| BMP7 | DEXAMETHASONE | DEXAME-THASONE | DEXAMETHASONE | Definite | |
| BRAF | FLUOROURACIL | NA | NA | NA | |
| BRAF | RIFAMPIN | NA | NA | NA | |

*Continued on next page*

Table S3 – *Continued from previous page*

| Gene | Drug Name | Score | Type/Mechanism | Approval |
|---|---|---|---|---|
| BRCA1 | BLEOMYCIN | BLEOMY-CIN | BLEOMYCIN | Ambiguous |
| BRCA1 | CALYCOSIN | CALYCO-SIN | CALYCOSIN | Definite |
| BRD2 | ACETAMINOPHEN | ACETAM-INOPHEN | ACETAMINOPHEN | Definite |
| BRD2 | TRIAZOLAM | TRIAZO-LAM | TRIAZOLAM | Definite |
| BRD3 | TRIAZOLAM | TRIAZO-LAM | TRIAZOLAM | Definite |
| BRD4 | TRIAZOLAM | TRIAZO-LAM | TRIAZOLAM | Definite |
| BRDT | TRIAZOLAM | TRIAZO-LAM | TRIAZOLAM | Definite |
| CACNA1C | CINNARIZINE | NA | NA | NA |
| CACNA1D | CINNARIZINE | NA | NA | NA |
| CACNA1F | CINNARIZINE | NA | NA | NA |
| CACNA1S | ATORVASTATIN CALCIUM TRIHYDRATE | NA | NA | NA |
| CACNA1S | CAFFEINE | NA | NA | NA |
| CACNA1S | CINNARIZINE | NA | NA | NA |
| CALR | GENTAMICIN | GENTAM-ICIN | GENTAMICIN | Definite |
| CALY | TRIFLUOPERAZINE | TRIFLU-OPERAZ-INE | TRIFLUOPERAZINE | Definite |
| CAMK2B | CAFFEINE | CAFFEINE | CAFFEINE | Definite |
| CAMK2B | HYDROGEN PEROXIDE | NA | NA | NA |
| CAMK2B | TRIFLUOPERAZINE | TRIFLU-OPERAZ-INE | TRIFLUOPERAZINE | Definite |
| CARTPT | DEXAMETHASONE | DEXAME-THASONE | DEXAMETHASONE | Definite |
| CARTPT | DEXTROAMPHETAMINE SULFATE | NA | NA | NA |
| CASR | VITAMIN D | NA | NA | NA |
| CAST | EPINEPHRINE | EPINEP-HRINE | EPINEPHRINE | Definite |
| CAST | THROMBIN | NA | NA | NA |
| CBFB | CETIRIZINE HYDROCHLORID-E | NA | NA | NA |
| CBR1 | FLUOROURACIL | NA | NA | NA |
| CBR3 | FLUOROURACIL | NA | NA | NA |
| CBX1 | CEFOTAXIME | CEFOTA-XIME | CEFOTAXIME | Definite |
| CBX1 | CEFOTAXIME SODIUM | NA | NA | NA |
| CBX1 | CLOTRIMAZOLE | CLOTRI-MAZOLE | CLOTRIMAZOLE | Definite |
| CBX1 | OMEPRAZOLE | OMEPRA-ZOLE | OMEPRAZOLE | Definite |
| CBX1 | OXOLINIC ACID | OXOLINIC ACID | OXOLINIC ACID | Definite |
| CBX1 | PROCHLORPERAZINE | PROCHL-ORPERA-ZINE | PROCHLORPERAZINE | Definite |
| CBX1 | PROMETHAZINE | PROMET-HAZINE | PROMETHAZINE | Definite |
| CBX1 | PROMETHAZINE HYDROC-HLORIDE | NA | NA | NA |
| CBX1 | TRIFLUOPERAZINE | TRIFLU-OPERAZ-INE | TRIFLUOPERAZINE | Definite |
| CBX1 | TRIMETHOPRIM | TRIMET-HOPRIM | TRIMETHOPRIM | Definite |
| CCKBR | DIAZEPAM | DIAZEPAM | DIAZEPAM | Definite |
| CCL17 | BLEOMYCIN | BLEOMY-CIN | BLEOMYCIN | Ambiguous |
| CCL17 | DEXAMETHASONE | DEXAME-THASONE | DEXAMETHASONE | Definite |
| CCL17 | HYDROCORTISONE | HYDROC-ORTISONE | HYDROCORTISONE | Definite |
| CCL17 | HYDROCORTISONE BUTYRA-TE | NA | NA | NA |
| CCL17 | LORATADINE | LORATA-DINE | LORATADINE | Definite |
| CCL18 | CIPROFLOXACIN | NA | NA | NA |
| CCL18 | ENOXACIN | NA | NA | NA |

*Continued on next page*

Table S3 – *Continued from previous page*

| Gene | Drug Name | Score | Type/Mechanism | Approval |
|---|---|---|---|---|
| CCL18 | LEVOFLOXACIN ANHYDROUS | NA | NA | NA |
| CCN2 | HYDROGEN PEROXIDE | NA | NA | NA |
| CCN2 | THROMBIN | NA | NA | NA |
| CCN2 | VITAMIN E | VITAMIN E | VITAMIN E | Ambiguous |
| CCND1 | ACETAMINOPHEN | ACETAM-INOPHEN | ACETAMINOPHEN | Definite |
| CCND1 | FLUOROURACIL | NA | NA | NA |
| CCND1 | VITAMIN E | VITAMIN E | VITAMIN E | Ambiguous |
| CCNK | FLUOROURACIL | NA | NA | NA |
| CD1A | TRIAMCINOLONE | TRIAMC-INOLONE | TRIAMCINOLONE | Definite |
| CD38 | THROMBIN | NA | NA | NA |
| CD40LG | ATORVASTATIN CALCIUM TRIHYDRATE | NA | NA | NA |
| CD44 | ACETAMINOPHEN | NA | NA | NA |
| CD44 | GENTAMICIN | GENTAM-ICIN | GENTAMICIN | Definite |
| CD80 | METHYLPREDNISOLONE | METHYL-PREDNI-SOLONE | METHYLPREDNISOLONE | Definite |
| CD86 | DEXAMETHASONE | DEXAME-THASONE | DEXAMETHASONE | Definite |
| CDC25C | FLUOROURACIL | NA | NA | NA |
| CDH13 | DEXTROAMPHETAMINE SULFATE | NA | NA | NA |
| CDH17 | DEXAMETHASONE | DEXAME-THASONE | DEXAMETHASONE | Definite |
| CDK1 | CINNARIZINE | CINNAR-IZINE | CINNARIZINE | Definite |
| CDK1 | CLOTRIMAZOLE | CLOTRI-MAZOLE | CLOTRIMAZOLE | Definite |
| CDK1 | SERTRALINE | SERTRA-LINE | SERTRALINE | Definite |
| CDK1 | SERTRALINE HYDROCHLO-RIDE | NA | NA | NA |
| CDK2 | ACETAMINOPHEN | ACETAM-INOPHEN | ACETAMINOPHEN | Definite |
| CDK2 | DEXAMETHASONE | DEXAME-THASONE | DEXAMETHASONE | Definite |
| CDK4 | ASCORBIC ACID | NA | NA | NA |
| CDK4 | DEXAMETHASONE | DEXAME-THASONE | DEXAMETHASONE | Definite |
| CDK4 | PROGESTIN | NA | NA | NA |
| CDK6 | DEXAMETHASONE | DEXAME-THASONE | DEXAMETHASONE | Definite |
| CDKN1A | ACETAMINOPHEN | ACETAM-INOPHEN | ACETAMINOPHEN | Definite |
| CDKN1A | FLUOROURACIL | NA | NA | NA |
| CDKN1B | PROGESTIN | NA | NA | NA |
| CEP68 | ASPIRIN | NA | NA | NA |
| CFHR4 | PHENYTOIN | PHENYT-OIN | PHENYTOIN | Definite |
| CFHR4 | PHENYTOIN SODIUM | NA | NA | NA |
| CFL1 | CINNARIZINE | CINNAR-IZINE | CINNARIZINE | Definite |
| CFL1 | CLOTRIMAZOLE | CLOTRI-MAZOLE | CLOTRIMAZOLE | Definite |
| CFL1 | SERTRALINE | SERTRA-LINE | SERTRALINE | Definite |
| CFL1 | SERTRALINE HYDROCHLO-RIDE | NA | NA | NA |
| CHAT | DEXAMETHASONE | DEXAME-THASONE | DEXAMETHASONE | Definite |
| CHAT | SODIUM CHLORIDE | NA | NA | NA |
| CHIA | ASPIRIN | NA | NA | NA |
| CHKA | DEXAMETHASONE | DEXAME-THASONE | DEXAMETHASONE | Definite |
| CHN2 | FLUOROURACIL | NA | NA | NA |
| CHRM1 | METOCLOPRAMIDE | METOCL-OPRAMI-DE | METOCLOPRAMIDE | Definite |
| CHRM1 | METOCLOPRAMIDE HYDROC-HLORIDE | NA | NA | NA |
| CHRM1 | PROMETHAZINE | PROMET-HAZINE | PROMETHAZINE | Definite |

*Continued on next page*

Table S3 – *Continued from previous page*

| Gene | Drug Name | Score | Type/Mechanism | Approval |
|---|---|---|---|---|
| CHRM1 | PROMETHAZINE HYDROC-HLORIDE | NA | NA | NA |
| CHRM1 | SCOPOLAMINE | SCOPOL-AMINE | SCOPOLAMINE | Definite |
| CHRM2 | PROMETHAZINE | PROMET-HAZINE | PROMETHAZINE | Definite |
| CHRM2 | PROMETHAZINE HYDROC-HLORIDE | NA | NA | NA |
| CHRM2 | SCOPOLAMINE | SCOPOL-AMINE | SCOPOLAMINE | Definite |
| CHRM3 | DEXAMETHASONE | NA | NA | NA |
| CHRM3 | PROMETHAZINE | PROMET-HAZINE | PROMETHAZINE | Definite |
| CHRM3 | PROMETHAZINE HYDROC-HLORIDE | NA | NA | NA |
| CHRM4 | PROMETHAZINE | PROMET-HAZINE | PROMETHAZINE | Definite |
| CHRM4 | PROMETHAZINE HYDROC-HLORIDE | NA | NA | NA |
| CHRM5 | PROMETHAZINE | PROMET-HAZINE | PROMETHAZINE | Definite |
| CHRM5 | PROMETHAZINE HYDROC-HLORIDE | NA | NA | NA |
| CHRM5 | SCOPOLAMINE | NA | NA | NA |
| CHRM5 | SCOPOLAMINE HYDROBROM-IDE | SCOPOL-AMINE HYDROB-ROMIDE | SCOPOLAMINE HYDROBROMIDE | Definite |
| CHUK | ASCORBIC ACID | NA | NA | NA |
| CKM | SODIUM CHLORIDE | NA | NA | NA |
| CNR1 | ARIPIPRAZOLE | ARIPIP-RAZOLE | ARIPIPRAZOLE | Definite |
| COL18A1 | ASCORBIC ACID | NA | NA | NA |
| COL18A1 | ASPIRIN | NA | NA | NA |
| COL18A1 | THROMBIN | NA | NA | NA |
| COL18A1 | VITAMIN E | NA | NA | NA |
| COL26A1 | ASPIRIN | NA | NA | NA |
| COLEC10 | FLUOROURACIL | NA | NA | NA |
| COMT | ACETAMINOPHEN | NA | NA | NA |
| COMT | CODEINE ANHYDROUS | NA | NA | NA |
| COMT | HYDROCODONE | NA | NA | NA |
| COMT | IBUPROFEN, SODIUM SALT | NA | NA | NA |
| COMT | MODAFINIL | MODAFI-NIL | MODAFINIL | Definite |
| COMT | NOREPINEPHRINE | NOREPI-NEPHRINE | NOREPINEPHRINE | Definite |
| COMT | QUETIAPINE FUMARATE | NA | NA | NA |
| COMT | SERTRALINE | SERTRA-LINE | SERTRALINE | Definite |
| COMT | SERTRALINE HYDROCHLO-RIDE | NA | NA | NA |
| COMT | VENLAFAXINE | VENLAF-AXINE | VENLAFAXINE | Definite |
| COQ2 | ATORVASTATIN CALCIUM TRIHYDRATE | NA | NA | NA |
| COX8A | ACETAMINOPHEN | NA | NA | NA |
| COX8A | ASPIRIN | NA | NA | NA |
| CP | AMPICILLIN | AMPICI-LLIN | AMPICILLIN | Definite |
| CP | AMPICILLIN (ANHYDROUS) | NA | NA | NA |
| CREBBP | TRIAZOLAM | TRIAZO-LAM | TRIAZOLAM | Definite |
| CRH | NALOXONE HYDROCHLORIDE | NA | NA | NA |
| CRHR1 | TRIAMCINOLONE | TRIAMC-INOLONE | TRIAMCINOLONE | Definite |
| CRISPLD2 | DEXAMETHASONE | DEXAME-THASONE | DEXAMETHASONE | Definite |
| CSF1R | PROGESTIN | NA | NA | NA |
| CSF2 | AMIKACIN | AMIKACIN | AMIKACIN | Definite |
| CSF2 | CETIRIZINE | CETIRI-ZINE | CETIRIZINE | Definite |
| CSF2 | CETIRIZINE HYDROCHLORID-E | NA | NA | NA |
| CSF2 | OMEPRAZOLE | OMEPRA-ZOLE | OMEPRAZOLE | Definite |

*Continued on next page*

Table S3 – *Continued from previous page*

| Gene | Drug Name | Score | Type/Mechanism | Approval |
|---|---|---|---|---|
| CSF2 | TRIFLUOPERAZINE | TRIFLU-OPERAZ-INE | TRIFLUOPERAZINE | Definite |
| CTDSP1 | CEPHALOTHIN | CEPHAL-OTHIN | CEPHALOTHIN | Definite |
| CTLA4 | BLEOMYCIN | BLEOMY-CIN | BLEOMYCIN | Ambiguous |
| CTLA4 | DEXAMETHASONE | DEXAME-THASONE | DEXAMETHASONE | Definite |
| CTNNB1 | DEXAMETHASONE | DEXAME-THASONE | DEXAMETHASONE | Definite |
| CUX2 | RIFAMPIN | RIFAMPIN | RIFAMPIN | Definite |
| CXCL10 | ATORVASTATIN CALCIUM TRIHYDRATE | NA | NA | NA |
| CXCL10 | METHYLPREDNISOLONE | METHYL-PREDNI-SOLONE | METHYLPREDNISOLONE | Definite |
| CXCL8 | ACETAMINOPHEN | ACETAM-INOPHEN | ACETAMINOPHEN | Definite |
| CXCL8 | ASPIRIN | NA | NA | NA |
| CXCL8 | CEFTRIAXONE | CEFTRI-AXONE | CEFTRIAXONE | Definite |
| CXCL8 | CLARITHROMYCIN | CLARIT-HROMYC-IN | CLARITHROMYCIN | Definite |
| CXCL8 | FLUOROURACIL | NA | NA | NA |
| CXCL8 | IBUPROFEN | IBUPRO-FEN | IBUPROFEN | Definite |
| CXCL8 | IBUPROFEN, SODIUM SALT | NA | NA | NA |
| CXCL8 | OMEPRAZOLE | OMEPRA-ZOLE | OMEPRAZOLE | Definite |
| CXCR1 | IBUPROFEN | IBUPRO-FEN | IBUPROFEN | Definite |
| CXCR1 | IBUPROFEN, SODIUM SALT | NA | NA | NA |
| CXCR2 | CLOTRIMAZOLE | CLOTRI-MAZOLE | CLOTRIMAZOLE | Definite |
| CXCR2 | IBUPROFEN | IBUPRO-FEN | IBUPROFEN | Definite |
| CXCR2 | IBUPROFEN, SODIUM SALT | NA | NA | NA |
| CXCR2 | VITAMIN E | VITAMIN E | VITAMIN E | Ambiguous |
| CYB5R1 | METOCLOPRAMIDE | METOCL-OPRAMI-DE | METOCLOPRAMIDE | Definite |
| CYB5R1 | METOCLOPRAMIDE HYDROC-HLORIDE | NA | NA | NA |
| CYB5R2 | METOCLOPRAMIDE | METOCL-OPRAMI-DE | METOCLOPRAMIDE | Definite |
| CYB5R2 | METOCLOPRAMIDE HYDROC-HLORIDE | NA | NA | NA |
| CYB5R3 | CEFTRIAXONE | CEFTRI-AXONE | CEFTRIAXONE | Definite |
| CYB5R3 | METOCLOPRAMIDE | METOCL-OPRAMI-DE | METOCLOPRAMIDE | Definite |
| CYB5R3 | METOCLOPRAMIDE HYDROC-HLORIDE | NA | NA | NA |
| CYB5R4 | METOCLOPRAMIDE | METOCL-OPRAMI-DE | METOCLOPRAMIDE | Definite |
| CYB5R4 | METOCLOPRAMIDE HYDROC-HLORIDE | NA | NA | NA |
| CYCSP5 | PHENYTOIN SODIUM | NA | NA | NA |
| CYP11B1 | METYRAPONE | NA | NA | NA |
| CYP11B2 | METOCLOPRAMIDE | METOCL-OPRAMI-DE | METOCLOPRAMIDE | Definite |
| CYP11B2 | METOCLOPRAMIDE HYDROC-HLORIDE | NA | NA | NA |
| CYP19A1 | ALENDRONATE SODIUM | NA | NA | NA |
| CYP19A1 | ASCORBIC ACID | NA | NA | NA |
| CYP19A1 | FLUOROURACIL | NA | NA | NA |
| CYP19A1 | IBUPROFEN | IBUPRO-FEN | IBUPROFEN | Definite |
| CYP19A1 | IBUPROFEN, SODIUM SALT | NA | NA | NA |
| CYP19A1 | PHENYTOIN | PHENYT-OIN | PHENYTOIN | Definite |
| CYP19A1 | PHENYTOIN SODIUM | NA | NA | NA |

*Continued on next page*

Table S3 – *Continued from previous page*

| Gene | Drug Name | Score | Type/Mechanism | Approval |
|---|---|---|---|---|
| CYP1A1 | PHENYTOIN | PHENYT- OIN | PHENYTOIN | Definite |
| CYP1A1 | PHENYTOIN SODIUM | NA | NA | NA |
| CYP1A1 | PROMETHAZINE | PROMET- HAZINE | PROMETHAZINE | Definite |
| CYP1A1 | PROMETHAZINE HYDROC- HLORIDE | NA | NA | NA |
| CYP1A2 | ACETAMINOPHEN | ACETAM- INOPHEN | ACETAMINOPHEN | Definite |
| CYP1A2 | AMOXICILLIN | AMOXIC- ILLIN | AMOXICILLIN | Definite |
| CYP1A2 | AMOXICILLIN ANHYDROUS | NA | NA | NA |
| CYP1A2 | ASPIRIN | NA | NA | NA |
| CYP1A2 | AZITHROMYCIN | AZITHR- OMYCIN | AZITHROMYCIN | Definite |
| CYP1A2 | CAFFEINE | CAFFEINE | CAFFEINE | Definite |
| CYP1A2 | CEFTRIAXONE | CEFTRI- AXONE | CEFTRIAXONE | Definite |
| CYP1A2 | CHLORZOXAZONE | CHLORZ- OXAZONE | CHLORZOXAZONE | Definite |
| CYP1A2 | CIPROFLOXACIN | CIPROF- LOXACIN | CIPROFLOXACIN | Definite |
| CYP1A2 | CLARITHROMYCIN | CLARIT- HROMYC- IN | CLARITHROMYCIN | Definite |
| CYP1A2 | CLOTRIMAZOLE | CLOTRI- MAZOLE | CLOTRIMAZOLE | Definite |
| CYP1A2 | DEXAMETHASONE | DEXAME- THASONE | DEXAMETHASONE | Definite |
| CYP1A2 | DIPHENHYDRAMINE | DIPHEN- HYDRAM- INE | DIPHENHYDRAMINE | Definite |
| CYP1A2 | ERYTHROMYCIN | ERYTHR- OMYCIN | ERYTHROMYCIN | Definite |
| CYP1A2 | FLUCONAZOLE | NA | NA | NA |
| CYP1A2 | GENTAMICIN | GENTAM- ICIN | GENTAMICIN | Definite |
| CYP1A2 | GLYCINE | GLYCINE | GLYCINE | Definite |
| CYP1A2 | IBUPROFEN | IBUPRO- FEN | IBUPROFEN | Definite |
| CYP1A2 | IBUPROFEN, SODIUM SALT | NA | NA | NA |
| CYP1A2 | LINEZOLID | LINEZOLID | LINEZOLID | Definite |
| CYP1A2 | MECLIZINE | MECLIZ- INE | MECLIZINE | Definite |
| CYP1A2 | MELATONIN | MELATO- NIN | MELATONIN | Definite |
| CYP1A2 | MERCAPTOPURINE | NA | NA | NA |
| CYP1A2 | METOCLOPRAMIDE | METOCL- OPRAMI- DE | METOCLOPRAMIDE | Definite |
| CYP1A2 | METOCLOPRAMIDE HYDROC- HLORIDE | NA | NA | NA |
| CYP1A2 | MINOCYCLINE | MINOCY- CLINE | MINOCYCLINE | Definite |
| CYP1A2 | MODAFINIL | MODAFI- NIL | MODAFINIL | Definite |
| CYP1A2 | NITROFURANTOIN | NITROF- URANTOIN | NITROFURANTOIN | Definite |
| CYP1A2 | NITROFURANTOIN, MACROC- RYSTALS | NA | NA | NA |
| CYP1A2 | OFLOXACIN | OFLOXA- CIN | OFLOXACIN | Definite |
| CYP1A2 | OMEPRAZOLE | OMEPRA- ZOLE | OMEPRAZOLE | Definite |
| CYP1A2 | PHENYTOIN | PHENYT- OIN | PHENYTOIN | Definite |
| CYP1A2 | PHENYTOIN SODIUM | NA | NA | NA |
| CYP1A2 | PROCHLORPERAZINE | PROCHL- ORPERA- ZINE | PROCHLORPERAZINE | Definite |
| CYP1A2 | PROMETHAZINE | PROMET- HAZINE | PROMETHAZINE | Definite |
| CYP1A2 | PROMETHAZINE HYDROC- HLORIDE | NA | NA | NA |
| CYP1A2 | RIFAMPIN | RIFAMPIN | RIFAMPIN | Definite |
| CYP1A2 | SILVER SULFADIAZINE | SILVER SULFAD- IAZINE | SILVER SULFADIAZINE | Definite |

*Continued on next page*

Table S3 – *Continued from previous page*

| Gene | Drug Name | Score | Type/Mechanism | Approval |
|---|---|---|---|---|
| CYP1A2 | TETRACYCLINE | TETRAC-YCLINE | TETRACYCLINE | Definite |
| CYP1A2 | TRIAMCINOLONE | TRIAMC-INOLONE | TRIAMCINOLONE | Definite |
| CYP1A2 | TRIFLUOPERAZINE | TRIFLU-OPERAZ-INE | TRIFLUOPERAZINE | Definite |
| CYP1A2 | TRIMETHOPRIM | TRIMET-HOPRIM | TRIMETHOPRIM | Definite |
| CYP1B1 | FLUOROURACIL | NA | NA | NA |
| CYP1B1 | RIFAMPIN | RIFAMPIN | RIFAMPIN | Definite |
| CYP24A1 | VITAMIN D | NA | NA | NA |
| CYP27B1 | RIFAMPIN | NA | NA | NA |
| CYP2A6 | ACETAMINOPHEN | ACETAM-INOPHEN | ACETAMINOPHEN | Definite |
| CYP2A6 | CAFFEINE | CAFFEINE | CAFFEINE | Definite |
| CYP2A6 | FLUOROURACIL | NA | NA | NA |
| CYP2A6 | PHENYTOIN | PHENYT-OIN | PHENYTOIN | Definite |
| CYP2A6 | PHENYTOIN SODIUM | NA | NA | NA |
| CYP2B6 | DIAZEPAM | DIAZEPAM | DIAZEPAM | Definite |
| CYP2B6 | KETAMINE | KETAMI-NE | KETAMINE | Definite |
| CYP2B6 | KETAMINE HYDROCHLORIDE | NA | NA | NA |
| CYP2B6 | PHENYTOIN | PHENYT-OIN | PHENYTOIN | Definite |
| CYP2B6 | RIFAMPIN | RIFAMPIN | RIFAMPIN | Definite |
| CYP2B6 | SERTRALINE | SERTRA-LINE | SERTRALINE | Definite |
| CYP2C19 | ACETAMINOPHEN | ACETAM-INOPHEN | ACETAMINOPHEN | Definite |
| CYP2C19 | AMOXICILLIN | AMOXIC-ILLIN | AMOXICILLIN | Definite |
| CYP2C19 | AMOXICILLIN ANHYDROUS | NA | NA | NA |
| CYP2C19 | ATORVASTATIN CALCIUM TRIHYDRATE | NA | NA | NA |
| CYP2C19 | AZITHROMYCIN | AZITHR-OMYCIN | AZITHROMYCIN | Definite |
| CYP2C19 | CAFFEINE | CAFFEINE | CAFFEINE | Definite |
| CYP2C19 | CEFTRIAXONE | CEFTRI-AXONE | CEFTRIAXONE | Definite |
| CYP2C19 | CHLORAMPHENICOL | CHLORA-MPHENI-COL | CHLORAMPHENICOL | Definite |
| CYP2C19 | CIPROFLOXACIN | CIPROF-LOXACIN | CIPROFLOXACIN | Definite |
| CYP2C19 | CLARITHROMYCIN | CLARIT-HROMYC-IN | CLARITHROMYCIN | Definite |
| CYP2C19 | CLOTRIMAZOLE | CLOTRI-MAZOLE | CLOTRIMAZOLE | Definite |
| CYP2C19 | DEXAMETHASONE | DEXAME-THASONE | DEXAMETHASONE | Definite |
| CYP2C19 | DIAZEPAM | DIAZEPAM | DIAZEPAM | Definite |
| CYP2C19 | DIPHENHYDRAMINE | DIPHEN-HYDRAM-INE | DIPHENHYDRAMINE | Definite |
| CYP2C19 | ERYTHROMYCIN | ERYTHR-OMYCIN | ERYTHROMYCIN | Definite |
| CYP2C19 | FLUCONAZOLE | NA | NA | NA |
| CYP2C19 | FLUOROURACIL | NA | NA | NA |
| CYP2C19 | GENTAMICIN | GENTAM-ICIN | GENTAMICIN | Definite |
| CYP2C19 | GLYCINE | GLYCINE | GLYCINE | Definite |
| CYP2C19 | IBUPROFEN | IBUPRO-FEN | IBUPROFEN | Definite |
| CYP2C19 | IBUPROFEN, SODIUM SALT | NA | NA | NA |
| CYP2C19 | LORATADINE | LORATA-DINE | LORATADINE | Definite |
| CYP2C19 | MERCAPTOPURINE | NA | NA | NA |
| CYP2C19 | MINOCYCLINE | MINOCY-CLINE | MINOCYCLINE | Definite |
| CYP2C19 | MODAFINIL | MODAFI-NIL | MODAFINIL | Definite |
| CYP2C19 | NITROFURANTOIN | NITROF-URANTOIN | NITROFURANTOIN | Definite |

*Continued on next page*

Table S3 – *Continued from previous page*

| Gene | Drug Name | Score | Type/Mechanism | Approval | |
|---|---|---|---|---|---|
| CYP2C19 | NITROFURANTOIN, MACROC-RYSTALS | NA | NA | NA | |
| CYP2C19 | OFLOXACIN | OFLOXA-CIN | OFLOXACIN | Definite | |
| CYP2C19 | OMEPRAZOLE | OMEPRA-ZOLE | OMEPRAZOLE | Definite | |
| CYP2C19 | PHENYTOIN | PHENYT-OIN | PHENYTOIN | Definite | |
| CYP2C19 | PHENYTOIN SODIUM | NA | NA | NA | |
| CYP2C19 | PROCHLORPERAZINE | PROCHL-ORPERA-ZINE | PROCHLORPERAZINE | Definite | |
| CYP2C19 | PSEUDOEPHEDRINE | PSEUDO-EPHEDR-INE | PSEUDOEPHEDRINE | Definite | |
| CYP2C19 | RIFAMPIN | RIFAMPIN | RIFAMPIN | Definite | |
| CYP2C19 | SILVER SULFADIAZINE | SILVER SULFAD-IAZINE | SILVER SULFADIAZINE | Definite | |
| CYP2C19 | TEMAZEPAM | TEMAZE-PAM | TEMAZEPAM | Definite | |
| CYP2C19 | TETRACYCLINE | TETRAC-YCLINE | TETRACYCLINE | Definite | |
| CYP2C19 | TRIAMCINOLONE | TRIAMC-INOLONE | TRIAMCINOLONE | Definite | |
| CYP2C19 | TRIFLUOPERAZINE | TRIFLU-OPERAZ-INE | TRIFLUOPERAZINE | Definite | |
| CYP2C19 | TRIMETHOPRIM | TRIMET-HOPRIM | TRIMETHOPRIM | Definite | |
| CYP2C8 | ATORVASTATIN CALCIUM TRIHYDRATE | NA | NA | NA | |
| CYP2C8 | FLUOROURACIL | NA | NA | NA | |
| CYP2C8 | IBUPROFEN | IBUPRO-FEN | IBUPROFEN | Definite | |
| CYP2C8 | IBUPROFEN, SODIUM SALT | NA | NA | NA | |
| CYP2C8 | RIFAMPIN | RIFAMPIN | RIFAMPIN | Definite | |
| CYP2C8 | TRIMETHOPRIM | TRIMET-HOPRIM | TRIMETHOPRIM | Definite | |
| CYP2C9 | ACETAMINOPHEN | ACETAM-INOPHEN | ACETAMINOPHEN | Definite | |
| CYP2C9 | AMOXICILLIN | AMOXIC-ILLIN | AMOXICILLIN | Definite | |
| CYP2C9 | AMOXICILLIN ANHYDROUS | NA | NA | NA | |
| CYP2C9 | ASPIRIN | NA | NA | NA | |
| CYP2C9 | ATORVASTATIN CALCIUM TRIHYDRATE | NA | NA | NA | |
| CYP2C9 | AZITHROMYCIN | AZITHR-OMYCIN | AZITHROMYCIN | Definite | |
| CYP2C9 | CAFFEINE | CAFFEINE | CAFFEINE | Definite | |
| CYP2C9 | CEFTRIAXONE | CEFTRI-AXONE | CEFTRIAXONE | Definite | |
| CYP2C9 | CIPROFLOXACIN | CIPROF-LOXACIN | CIPROFLOXACIN | Definite | |
| CYP2C9 | CLARITHROMYCIN | CLARIT-HROMYC-IN | CLARITHROMYCIN | Definite | |
| CYP2C9 | CLOTRIMAZOLE | CLOTRI-MAZOLE | CLOTRIMAZOLE | Definite | |
| CYP2C9 | DEXAMETHASONE | DEXAME-THASONE | DEXAMETHASONE | Definite | |
| CYP2C9 | DIAZEPAM | DIAZEPAM | DIAZEPAM | Definite | |
| CYP2C9 | DIPHENHYDRAMINE | DIPHEN-HYDRAM-INE | DIPHENHYDRAMINE | Definite | |
| CYP2C9 | ERYTHROMYCIN | ERYTHR-OMYCIN | ERYTHROMYCIN | Definite | |
| CYP2C9 | FLUCONAZOLE | NA | NA | NA | |
| CYP2C9 | GENTAMICIN | GENTAM-ICIN | GENTAMICIN | Definite | |
| CYP2C9 | GLYCINE | GLYCINE | GLYCINE | Definite | |
| CYP2C9 | IBUPROFEN | IBUPRO-FEN | IBUPROFEN | Definite | |
| CYP2C9 | IBUPROFEN, SODIUM SALT | NA | NA | NA | |
| CYP2C9 | LINEZOLID | LINEZOLID | LINEZOLID | Definite | |
| CYP2C9 | LORATADINE | LORATA-DINE | LORATADINE | Definite | |

*Continued on next page*

Table S3 – *Continued from previous page*

| Gene | Drug Name | Score | Type/Mechanism | Approval |
|---|---|---|---|---|
| CYP2C9 | MECLIZINE | MECLIZ-INE | MECLIZINE | Definite |
| CYP2C9 | MERCAPTOPURINE | NA | NA | NA |
| CYP2C9 | MINOCYCLINE | MINOCY-CLINE | MINOCYCLINE | Definite |
| CYP2C9 | MODAFINIL | MODAFI-NIL | MODAFINIL | Definite |
| CYP2C9 | NITROFURANTOIN | NITROF-URANTOIN | NITROFURANTOIN | Definite |
| CYP2C9 | NITROFURANTOIN, MACROC-RYSTALS | NA | NA | NA |
| CYP2C9 | OFLOXACIN | OFLOXA-CIN | OFLOXACIN | Definite |
| CYP2C9 | OMEPRAZOLE | OMEPRA-ZOLE | OMEPRAZOLE | Definite |
| CYP2C9 | PHENYTOIN | PHENYT-OIN | PHENYTOIN | Definite |
| CYP2C9 | PHENYTOIN SODIUM | NA | NA | NA |
| CYP2C9 | PROCHLORPERAZINE | PROCHL-ORPERA-ZINE | PROCHLORPERAZINE | Definite |
| CYP2C9 | PROPOFOL | PROPOF-OL | PROPOFOL | Definite |
| CYP2C9 | PSEUDOEPHEDRINE | PSEUDO-EPHEDR-INE | PSEUDOEPHEDRINE | Definite |
| CYP2C9 | RIFAMPIN | RIFAMPIN | RIFAMPIN | Definite |
| CYP2C9 | SILVER SULFADIAZINE | SILVER SULFAD-IAZINE | SILVER SULFADIAZINE | Definite |
| CYP2C9 | SULFAMETHOXAZOLE | SULFAM-ETHOXA-ZOLE | SULFAMETHOXAZOLE | Definite |
| CYP2C9 | TETRACYCLINE | TETRAC-YCLINE | TETRACYCLINE | Definite |
| CYP2C9 | TRIAMCINOLONE | TRIAMC-INOLONE | TRIAMCINOLONE | Definite |
| CYP2C9 | TRIMETHOPRIM | TRIMET-HOPRIM | TRIMETHOPRIM | Definite |
| CYP2D6 | ACETAMINOPHEN | ACETAM-INOPHEN | ACETAMINOPHEN | Definite |
| CYP2D6 | AMOXICILLIN | AMOXIC-ILLIN | AMOXICILLIN | Definite |
| CYP2D6 | AMOXICILLIN ANHYDROUS | NA | NA | NA |
| CYP2D6 | ARIPIPRAZOLE | ARIPIP-RAZOLE | ARIPIPRAZOLE | Definite |
| CYP2D6 | ASPIRIN | NA | NA | NA |
| CYP2D6 | ATORVASTATIN CALCIUM TRIHYDRATE | NA | NA | NA |
| CYP2D6 | AZITHROMYCIN | AZITHR-OMYCIN | AZITHROMYCIN | Definite |
| CYP2D6 | CAFFEINE | CAFFEINE | CAFFEINE | Definite |
| CYP2D6 | CEFTRIAXONE | CEFTRI-AXONE | CEFTRIAXONE | Definite |
| CYP2D6 | CIPROFLOXACIN | CIPROF-LOXACIN | CIPROFLOXACIN | Definite |
| CYP2D6 | CLARITHROMYCIN | CLARIT-HROMYC-IN | CLARITHROMYCIN | Definite |
| CYP2D6 | CLOTRIMAZOLE | CLOTRI-MAZOLE | CLOTRIMAZOLE | Definite |
| CYP2D6 | CODEINE | CODEINE | CODEINE | Definite |
| CYP2D6 | CODEINE ANHYDROUS | NA | NA | NA |
| CYP2D6 | DEXAMETHASONE | DEXAME-THASONE | DEXAMETHASONE | Definite |
| CYP2D6 | DEXTROAMPHETAMINE SULFATE | NA | NA | NA |
| CYP2D6 | DIMENHYDRINATE | DIMENH-YDRINATE | DIMENHYDRINATE | Definite |
| CYP2D6 | DIPHENHYDRAMINE | DIPHEN-HYDRAM-INE | DIPHENHYDRAMINE | Definite |
| CYP2D6 | ERYTHROMYCIN | ERYTHR-OMYCIN | ERYTHROMYCIN | Definite |
| CYP2D6 | FLUCONAZOLE | NA | NA | NA |
| CYP2D6 | GENTAMICIN | GENTAM-ICIN | GENTAMICIN | Definite |
| CYP2D6 | GLYCINE | GLYCINE | GLYCINE | Definite |

*Continued on next page*

Table S3 – *Continued from previous page*

| Gene | Drug Name | Score | Type/Mechanism | Approval | |
|---|---|---|---|---|---|
| CYP2D6 | HYDROCODONE | HYDROC-ODONE | HYDROCODONE | Definite | |
| CYP2D6 | IBUPROFEN | IBUPRO-FEN | IBUPROFEN | Definite | |
| CYP2D6 | IBUPROFEN, SODIUM SALT | NA | NA | NA | |
| CYP2D6 | LINEZOLID | LINEZOLID | LINEZOLID | Definite | |
| CYP2D6 | LOPERAMIDE | LOPERA-MIDE | LOPERAMIDE | Definite | |
| CYP2D6 | LORATADINE | LORATA-DINE | LORATADINE | Definite | |
| CYP2D6 | MECLIZINE | MECLIZ-INE | MECLIZINE | Definite | |
| CYP2D6 | MERCAPTOPURINE | NA | NA | NA | |
| CYP2D6 | METOCLOPRAMIDE | METOCL-OPRAMI-DE | METOCLOPRAMIDE | Definite | |
| CYP2D6 | METOCLOPRAMIDE HYDROC-HLORIDE | NA | NA | NA | |
| CYP2D6 | METOPROLOL | METOPR-OLOL | METOPROLOL | Definite | |
| CYP2D6 | MINOCYCLINE | MINOCY-CLINE | MINOCYCLINE | Definite | |
| CYP2D6 | MODAFINIL | MODAFI-NIL | MODAFINIL | Definite | |
| CYP2D6 | NITROFURANTOIN | NITROF-URANTOIN | NITROFURANTOIN | Definite | |
| CYP2D6 | NITROFURANTOIN, MACROC-RYSTALS | NA | NA | NA | |
| CYP2D6 | OFLOXACIN | OFLOXA-CIN | OFLOXACIN | Definite | |
| CYP2D6 | OMEPRAZOLE | OMEPRA-ZOLE | OMEPRAZOLE | Definite | |
| CYP2D6 | ONDANSETRON | ONDANS-ETRON | ONDANSETRON | Definite | |
| CYP2D6 | ONDANSETRON HYDROCHLO-RIDE | NA | NA | NA | |
| CYP2D6 | OXYMETAZOLINE | OXYMET-AZOLINE | OXYMETAZOLINE | Definite | |
| CYP2D6 | OXYMETAZOLINE HYDROC-HLORIDE | NA | NA | NA | |
| CYP2D6 | PHENYTOIN | PHENYT-OIN | PHENYTOIN | Definite | |
| CYP2D6 | PHENYTOIN SODIUM | NA | NA | NA | |
| CYP2D6 | PROCHLORPERAZINE | PROCHL-ORPERA-ZINE | PROCHLORPERAZINE | Definite | |
| CYP2D6 | PROMETHAZINE | PROMET-HAZINE | PROMETHAZINE | Definite | |
| CYP2D6 | PROMETHAZINE HYDROC-HLORIDE | NA | NA | NA | |
| CYP2D6 | PSEUDOEPHEDRINE | PSEUDO-EPHEDR-INE | PSEUDOEPHEDRINE | Definite | |
| CYP2D6 | RIFAMPIN | RIFAMPIN | RIFAMPIN | Definite | |
| CYP2D6 | TAMSULOSIN | TAMSUL-OSIN | TAMSULOSIN | Definite | |
| CYP2D6 | TAMSULOSIN HYDROCHLO-RIDE | NA | NA | NA | |
| CYP2D6 | TERBINAFINE | TERBIN-AFINE | TERBINAFINE | Definite | |
| CYP2D6 | TETRACYCLINE | TETRAC-YCLINE | TETRACYCLINE | Definite | |
| CYP2D6 | TRIAMCINOLONE | TRIAMC-INOLONE | TRIAMCINOLONE | Definite | |
| CYP2D6 | TRIFLUOPERAZINE | TRIFLU-OPERAZ-INE | TRIFLUOPERAZINE | Definite | |
| CYP2D6 | TRIMETHOPRIM | TRIMET-HOPRIM | TRIMETHOPRIM | Definite | |
| CYP2D6 | VENLAFAXINE | VENLAF-AXINE | VENLAFAXINE | Definite | |
| CYP2E1 | ACETAMINOPHEN | ACETAM-INOPHEN | ACETAMINOPHEN | Definite | |
| CYP2E1 | CIPROFLOXACIN | CIPROF-LOXACIN | CIPROFLOXACIN | Definite | |
| CYP2E1 | FLUOROURACIL | NA | NA | NA | |
| CYP2E1 | STREPTOMYCIN | NA | NA | NA | |
| CYP3A | CLARITHROMYCIN | NA | NA | NA | |

*Continued on next page*

Table S3 – *Continued from previous page*

| Gene | Drug Name | Score | Type/Mechanism | Approval |
|---|---|---|---|---|
| CYP3A | OMEPRAZOLE | NA | NA | NA |
| CYP3A4 | ACETAMINOPHEN | ACETAM-INOPHEN | ACETAMINOPHEN | Definite |
| CYP3A4 | AMOXICILLIN | AMOXIC-ILLIN | AMOXICILLIN | Definite |
| CYP3A4 | AMOXICILLIN ANHYDROUS | NA | NA | NA |
| CYP3A4 | ASPIRIN | NA | NA | NA |
| CYP3A4 | ATORVASTATIN CALCIUM TRIHYDRATE | NA | NA | NA |
| CYP3A4 | AZITHROMYCIN | AZITHR-OMYCIN | AZITHROMYCIN | Definite |
| CYP3A4 | BISACODYL | BISACO-DYL | BISACODYL | Definite |
| CYP3A4 | BLEOMYCIN | BLEOMY-CIN | BLEOMYCIN | Ambiguous |
| CYP3A4 | CAFFEINE | CAFFEINE | CAFFEINE | Definite |
| CYP3A4 | CEFTRIAXONE | CEFTRI-AXONE | CEFTRIAXONE | Definite |
| CYP3A4 | CHLORAMPHENICOL | CHLORA-MPHENI-COL | CHLORAMPHENICOL | Definite |
| CYP3A4 | CHLORZOXAZONE | CHLORZ-OXAZONE | CHLORZOXAZONE | Definite |
| CYP3A4 | CIPROFLOXACIN | CIPROF-LOXACIN | CIPROFLOXACIN | Definite |
| CYP3A4 | CLARITHROMYCIN | CLARIT-HROMYC-IN | CLARITHROMYCIN | Definite |
| CYP3A4 | CLOTRIMAZOLE | CLOTRI-MAZOLE | CLOTRIMAZOLE | Definite |
| CYP3A4 | CODEINE | CODEINE | CODEINE | Definite |
| CYP3A4 | CODEINE ANHYDROUS | NA | NA | NA |
| CYP3A4 | DEQUALINIUM CHLORIDE | DEQUAL-INIUM CHLORIDE | DEQUALINIUM CHLORIDE | Definite |
| CYP3A4 | DEXAMETHASONE | DEXAME-THASONE | DEXAMETHASONE | Definite |
| CYP3A4 | DIAZEPAM | DIAZEPAM | DIAZEPAM | Definite |
| CYP3A4 | DIPHENHYDRAMINE | DIPHEN-HYDRAM-INE | DIPHENHYDRAMINE | Definite |
| CYP3A4 | ERYTHROMYCIN | ERYTHR-OMYCIN | ERYTHROMYCIN | Definite |
| CYP3A4 | FLUCONAZOLE | NA | NA | NA |
| CYP3A4 | FLUOROURACIL | NA | NA | NA |
| CYP3A4 | GENTAMICIN | GENTAM-ICIN | GENTAMICIN | Definite |
| CYP3A4 | GLYCINE | GLYCINE | GLYCINE | Definite |
| CYP3A4 | HYDROCORTISONE | HYDROC-ORTISONE | HYDROCORTISONE | Definite |
| CYP3A4 | HYDROCORTISONE BUTYRA-TE | NA | NA | NA |
| CYP3A4 | IBUPROFEN | IBUPRO-FEN | IBUPROFEN | Definite |
| CYP3A4 | IBUPROFEN, SODIUM SALT | NA | NA | NA |
| CYP3A4 | LIDOCAINE | LIDOCA-INE | LIDOCAINE | Definite |
| CYP3A4 | LINEZOLID | LINEZOLID | LINEZOLID | Definite |
| CYP3A4 | LOPERAMIDE | LOPERA-MIDE | LOPERAMIDE | Definite |
| CYP3A4 | MELATONIN | MELATO-NIN | MELATONIN | Definite |
| CYP3A4 | MERCAPTOPURINE | NA | NA | NA |
| CYP3A4 | METRONIDAZOLE | METRON-IDAZOLE | METRONIDAZOLE | Definite |
| CYP3A4 | METYRAPONE | NA | NA | NA |
| CYP3A4 | MINOCYCLINE | MINOCY-CLINE | MINOCYCLINE | Definite |
| CYP3A4 | MODAFINIL | MODAFI-NIL | MODAFINIL | Definite |
| CYP3A4 | NITROFURANTOIN | NITROF-URANTOIN | NITROFURANTOIN | Definite |
| CYP3A4 | NITROFURANTOIN, MACROC-RYSTALS | NA | NA | NA |
| CYP3A4 | OFLOXACIN | OFLOXA-CIN | OFLOXACIN | Definite |

*Continued on next page*

Table S3 − *Continued from previous page*

| Gene | Drug Name | Score | Type/Mechanism | Approval | |
|---|---|---|---|---|---|
| CYP3A4 | OMEPRAZOLE | OMEPRA-ZOLE | OMEPRAZOLE | Definite | |
| CYP3A4 | ONDANSETRON | ONDANS-ETRON | ONDANSETRON | Definite | |
| CYP3A4 | ONDANSETRON HYDROCHLO-RIDE | NA | NA | NA | |
| CYP3A4 | OXYMETAZOLINE | OXYMET-AZOLINE | OXYMETAZOLINE | Definite | |
| CYP3A4 | OXYMETAZOLINE HYDROC-HLORIDE | NA | NA | NA | |
| CYP3A4 | PHENYTOIN | PHENYT-OIN | PHENYTOIN | Definite | |
| CYP3A4 | PHENYTOIN SODIUM | NA | NA | NA | |
| CYP3A4 | PROCHLORPERAZINE | PROCHL-ORPERA-ZINE | PROCHLORPERAZINE | Definite | |
| CYP3A4 | PSEUDOEPHEDRINE | PSEUDO-EPHEDR-INE | PSEUDOEPHEDRINE | Definite | |
| CYP3A4 | QUETIAPINE FUMARATE | NA | NA | NA | |
| CYP3A4 | RIFAMPIN | RIFAMPIN | RIFAMPIN | Definite | |
| CYP3A4 | SERTRALINE | SERTRA-LINE | SERTRALINE | Definite | |
| CYP3A4 | SERTRALINE HYDROCHLO-RIDE | NA | NA | NA | |
| CYP3A4 | SULFADIAZINE | SULFAD-IAZINE | SULFADIAZINE | Definite | |
| CYP3A4 | SULFAMETHOXAZOLE | SULFAM-ETHOXA-ZOLE | SULFAMETHOXAZOLE | Definite | |
| CYP3A4 | TAMSULOSIN | TAMSUL-OSIN | TAMSULOSIN | Definite | |
| CYP3A4 | TAMSULOSIN HYDROCHLO-RIDE | NA | NA | NA | |
| CYP3A4 | TEMAZEPAM | TEMAZE-PAM | TEMAZEPAM | Definite | |
| CYP3A4 | TETRACYCLINE | TETRAC-YCLINE | TETRACYCLINE | Definite | |
| CYP3A4 | TRIAMCINOLONE | TRIAMC-INOLONE | TRIAMCINOLONE | Definite | |
| CYP3A4 | TRIAZOLAM | TRIAZO-LAM | TRIAZOLAM | Definite | |
| CYP3A4 | TRIFLUOPERAZINE | TRIFLU-OPERAZ-INE | TRIFLUOPERAZINE | Definite | |
| CYP3A4 | TRIMETHOPRIM | TRIMET-HOPRIM | TRIMETHOPRIM | Definite | |
| CYP3A4 | ZOLPIDEM | ZOLPIDEM | ZOLPIDEM | Definite | |
| CYP3A4 | ZOLPIDEM TARTRATE | NA | NA | NA | |
| CYP3A43 | CHLORZOXAZONE | CHLORZ-OXAZONE | CHLORZOXAZONE | Definite | |
| CYP3A43 | MELATONIN | MELATO-NIN | MELATONIN | Definite | |
| CYP3A43 | QUETIAPINE FUMARATE | NA | NA | NA | |
| CYP3A5 | APIXABAN | APIXABAN | APIXABAN | Definite | |
| CYP3A5 | CLARITHROMYCIN | CLARIT-HROMYC-IN | CLARITHROMYCIN | Definite | |
| CYP3A5 | CLINDAMYCIN | CLINDA-MYCIN | CLINDAMYCIN | Definite | |
| CYP3A5 | DIAZEPAM | DIAZEPAM | DIAZEPAM | Definite | |
| CYP3A5 | ERYTHROMYCIN | ERYTHR-OMYCIN | ERYTHROMYCIN | Definite | |
| CYP3A5 | ESZOPICLONE | ESZOPI-CLONE | ESZOPICLONE | Definite | |
| CYP3A5 | FLUCONAZOLE | NA | NA | NA | |
| CYP3A5 | HYDROCODONE | HYDROC-ODONE | HYDROCODONE | Definite | |
| CYP3A5 | IBUPROFEN, SODIUM SALT | NA | NA | NA | |
| CYP3A5 | LORATADINE | LORATA-DINE | LORATADINE | Definite | |
| CYP3A5 | MODAFINIL | MODAFI-NIL | MODAFINIL | Definite | |
| CYP3A5 | OMEPRAZOLE | OMEPRA-ZOLE | OMEPRAZOLE | Definite | |
| CYP3A5 | ONDANSETRON | ONDANS-ETRON | ONDANSETRON | Definite | |

*Continued on next page*

Table S3 – *Continued from previous page*

| Gene | Drug Name | Score | Type/Mechanism | Approval |
|---|---|---|---|---|
| CYP3A5 | ONDANSETRON HYDROCHLORIDE | NA | NA | NA |
| CYP3A5 | PHENYTOIN | PHENYTOIN | PHENYTOIN | Definite |
| CYP3A5 | PHENYTOIN SODIUM | NA | NA | NA |
| CYP3A5 | RIFAMPIN | RIFAMPIN | RIFAMPIN | Definite |
| CYP3A5 | SERTRALINE | SERTRALINE | SERTRALINE | Definite |
| CYP3A5 | TAMSULOSIN | TAMSULOSIN | TAMSULOSIN | Definite |
| CYP3A5 | TRIAZOLAM | TRIAZOLAM | TRIAZOLAM | Definite |
| CYP3A5 | VENLAFAXINE | VENLAFAXINE | VENLAFAXINE | Definite |
| CYP3A5 | ZOLPIDEM | ZOLPIDEM | ZOLPIDEM | Definite |
| CYP3A5 | ZOLPIDEM TARTRATE | NA | NA | NA |
| CYP3A7 | ATORVASTATIN CALCIUM TRIHYDRATE | NA | NA | NA |
| CYP3A7 | MELATONIN | MELATONIN | MELATONIN | Definite |
| CYP4F2 | ASPIRIN | NA | NA | NA |
| CYP51A1 | FLUCONAZOLE | NA | NA | NA |
| CYP7B1 | ACETAMINOPHEN | ACETAMINOPHEN | ACETAMINOPHEN | Definite |
| CYTH2 | CLOTRIMAZOLE | CLOTRIMAZOLE | CLOTRIMAZOLE | Definite |
| DCN | ASCORBIC ACID | NA | NA | NA |
| DDIT3 | ASCORBIC ACID | NA | NA | NA |
| DDIT3 | ASPIRIN | NA | NA | NA |
| DDIT3 | HYDROGEN PEROXIDE | NA | NA | NA |
| DDIT3 | IBUPROFEN | IBUPROFEN | IBUPROFEN | Definite |
| DDIT3 | IBUPROFEN, SODIUM SALT | NA | NA | NA |
| DDIT3 | VITAMIN E | VITAMIN E | VITAMIN E | Ambiguous |
| DEFA1 | HYDROCORTISONE | HYDROCORTISONE | HYDROCORTISONE | Definite |
| DEFA1 | HYDROCORTISONE BUTYRATE | NA | NA | NA |
| DEFA5 | SODIUM CHLORIDE | NA | NA | NA |
| DES | THROMBIN | NA | NA | NA |
| DHFR | TRIMETHOPRIM | TRIMETHOPRIM | TRIMETHOPRIM | Definite |
| DHFR | VITAMIN B12 | NA | NA | NA |
| DIABLO | HYDROGEN PEROXIDE | NA | NA | NA |
| DKK1 | ALENDRONATE SODIUM | NA | NA | NA |
| DKK1 | FLUOROURACIL | NA | NA | NA |
| DLG5 | FLUOROURACIL | NA | NA | NA |
| DNMT1 | CEPHALOTHIN | CEPHALOTHIN | CEPHALOTHIN | Definite |
| DNTT | ACYCLOVIR | NA | NA | NA |
| DOK5 | DEXAMETHASONE | DEXAMETHASONE | DEXAMETHASONE | Definite |
| DPEP1 | CILASTATIN | NA | NA | NA |
| DPEP1 | DEXAMETHASONE | DEXAMETHASONE | DEXAMETHASONE | Definite |
| DPP4 | VALACYCLOVIR | VALACYCLOVIR | VALACYCLOVIR | Definite |
| DPYD | FLUOROURACIL | NA | NA | NA |
| DRD1 | DEXTROAMPHETAMINE | DEXTROAMPHETAMINE | DEXTROAMPHETAMINE | Definite |
| DRD1 | DEXTROAMPHETAMINE SULFATE | NA | NA | NA |
| DRD1 | EPINEPHRINE | EPINEPHRINE | EPINEPHRINE | Definite |
| DRD1 | NOREPINEPHRINE | NOREPINEPHRINE | NOREPINEPHRINE | Definite |
| DRD1 | PHENYLEPHRINE | PHENYLEPHRINE | PHENYLEPHRINE | Definite |
| DRD1 | PROCHLORPERAZINE | PROCHLORPERAZINE | PROCHLORPERAZINE | Definite |
| DRD1 | PROMETHAZINE | PROMETHAZINE | PROMETHAZINE | Definite |

*Continued on next page*

Table S3 – *Continued from previous page*

| Gene | Drug Name | Score | Type/Mechanism | Approval |
|---|---|---|---|---|
| DRD1 | PROMETHAZINE HYDROCHLORIDE | NA | NA | NA |
| DRD1 | QUETIAPINE FUMARATE | NA | NA | NA |
| DRD1 | SERTRALINE | SERTRALINE | SERTRALINE | Definite |
| DRD1 | SERTRALINE HYDROCHLORIDE | NA | NA | NA |
| DRD1 | TRIFLUOPERAZINE | TRIFLUOPERAZINE | TRIFLUOPERAZINE | Definite |
| DRD2 | ARIPIPRAZOLE | ARIPIPRAZOLE | ARIPIPRAZOLE | Definite |
| DRD2 | CAFFEINE | NA | NA | NA |
| DRD2 | DEXTROAMPHETAMINE SULFATE | NA | NA | NA |
| DRD2 | METOCLOPRAMIDE | METOCLOPRAMIDE | METOCLOPRAMIDE | Definite |
| DRD2 | METOCLOPRAMIDE HYDROCHLORIDE | NA | NA | NA |
| DRD2 | PHENYLEPHRINE | PHENYLEPHRINE | PHENYLEPHRINE | Definite |
| DRD2 | PROCHLORPERAZINE | PROCHLORPERAZINE | PROCHLORPERAZINE | Definite |
| DRD2 | PROPOFOL | NA | NA | NA |
| DRD2 | PSEUDOEPHEDRINE | PSEUDOEPHEDRINE | PSEUDOEPHEDRINE | Definite |
| DRD2 | QUETIAPINE FUMARATE | QUETIAPINE FUMARATE | QUETIAPINE FUMARATE | Definite |
| DRD2 | TRIFLUOPERAZINE | TRIFLUOPERAZINE | TRIFLUOPERAZINE | Definite |
| DRD3 | ATORVASTATIN CALCIUM TRIHYDRATE | NA | NA | NA |
| DRD3 | QUETIAPINE FUMARATE | NA | NA | NA |
| DTYMK | FLUOROURACIL | NA | NA | NA |
| DUX1 | RIFAMPIN | NA | NA | NA |
| E2F1 | ASCORBIC ACID | NA | NA | NA |
| E2F1 | FLUOROURACIL | NA | NA | NA |
| EGFR | FLUOROURACIL | NA | NA | NA |
| EGFR | LIDOCAINE | LIDOCAINE | LIDOCAINE | Definite |
| EHMT2 | CEFAZOLIN | CEFAZOLIN | CEFAZOLIN | Definite |
| EHMT2 | CEFOTAXIME | CEFOTAXIME | CEFOTAXIME | Definite |
| EHMT2 | CEFOTAXIME SODIUM | NA | NA | NA |
| EHMT2 | CEPHALEXIN | CEPHALEXIN | CEPHALEXIN | Definite |
| EHMT2 | CEPHALEXIN ANHYDROUS | NA | NA | NA |
| EHMT2 | CEPHALOTHIN | CEPHALOTHIN | CEPHALOTHIN | Definite |
| EHMT2 | DEQUALINIUM CHLORIDE | DEQUALINIUM CHLORIDE | DEQUALINIUM CHLORIDE | Definite |
| EHMT2 | DOXYCYCLINE | DOXYCYCLINE | DOXYCYCLINE | Ambiguous |
| EHMT2 | DOXYCYCLINE ANHYDROUS | NA | NA | NA |
| EHMT2 | ENROFLOXACIN | ENROFLOXACIN | ENROFLOXACIN | Definite |
| EHMT2 | LOMEFLOXACIN | LOMEFLOXACIN | LOMEFLOXACIN | Definite |
| EHMT2 | NALIDIXIC ACID | NALIDIXIC ACID | NALIDIXIC ACID | Definite |
| EHMT2 | NALOXONE HYDROCHLORIDE | NALOXONE HYDROCHLORIDE | NALOXONE HYDROCHLORIDE | Definite |
| EHMT2 | NOREPINEPHRINE | NOREPINEPHRINE | NOREPINEPHRINE | Definite |
| EHMT2 | OMEPRAZOLE | OMEPRAZOLE | OMEPRAZOLE | Definite |

*Continued on next page*

Table S3 – *Continued from previous page*

| Gene | Drug Name | Score | Type/Mechanism | Approval |
|---|---|---|---|---|
| EHMT2 | PROCHLORPERAZINE | PROCHL-ORPERA-ZINE | PROCHLORPERAZINE | Definite |
| EHMT2 | PROMETHAZINE | PROMET-HAZINE | PROMETHAZINE | Definite |
| EHMT2 | PROMETHAZINE HYDROC-HLORIDE | PROMET-HAZINE HYDROC-HLORIDE | PROMETHAZINE HYDROCHLORIDE | Definite |
| EHMT2 | RISEDRONIC ACID | RISEDR-ONIC ACID | RISEDRONIC ACID | Definite |
| EHMT2 | SILVER SULFADIAZINE | NA | NA | NA |
| EHMT2 | STREPTOMYCIN | STREPT-OMYCIN | STREPTOMYCIN | Definite |
| EHMT2 | TRIFLUOPERAZINE | TRIFLU-OPERAZ-INE | TRIFLUOPERAZINE | Definite |
| EHMT2 | TRIPROLIDINE | TRIPRO-LIDINE | TRIPROLIDINE | Definite |
| EIF2AK4 | QUETIAPINE FUMARATE | NA | NA | NA |
| ENO2 | PROPOFOL | PROPOF-OL | PROPOFOL | Definite |
| EPHX1 | PHENYTOIN | PHENYT-OIN | PHENYTOIN | Definite |
| EPHX1 | PHENYTOIN SODIUM | NA | NA | NA |
| EPM2A | QUETIAPINE FUMARATE | NA | NA | NA |
| EPM2A | TRIFLUOPERAZINE | TRIFLU-OPERAZ-INE | TRIFLUOPERAZINE | Definite |
| ERBB2 | HYDROCORTISONE | HYDROC-ORTISONE | HYDROCORTISONE | Definite |
| ERBB2 | HYDROCORTISONE BUTYRA-TE | NA | NA | NA |
| ERBB2 | VITAMIN E | NA | NA | NA |
| ERCC1 | BLEOMYCIN | BLEOMY-CIN | BLEOMYCIN | Ambiguous |
| ERCC1 | FLUOROURACIL | NA | NA | NA |
| ERCC2 | BLEOMYCIN | BLEOMY-CIN | BLEOMYCIN | Ambiguous |
| ESR1 | BENZETHONIUM CHLORIDE | BENZET-HONIUM CHLORIDE | BENZETHONIUM CHLORIDE | Definite |
| ESR1 | DOXYCYCLINE | DOXYCY-CLINE | DOXYCYCLINE | Ambiguous |
| ESR1 | DOXYCYCLINE ANHYDROUS | NA | NA | NA |
| ESR1 | ESTROGEN | ESTROG-EN | ESTROGEN | Definite |
| ESR1 | PHENAZOPYRIDINE HYDROC-HLORIDE | NA | NA | NA |
| ESR2 | ESTROGEN | ESTROG-EN | ESTROGEN | Definite |
| EXO1 | FLUOROURACIL | NA | NA | NA |
| F10 | APIXABAN | APIXABAN | APIXABAN | Definite |
| F10 | THROMBIN | NA | NA | NA |
| F11 | THROMBIN | NA | NA | NA |
| F13A1 | ASPIRIN | NA | NA | NA |
| F2 | ENOXAPARIN | ENOXAP-ARIN | ENOXAPARIN | Definite |
| F2 | ENOXAPARIN SODIUM | NA | NA | NA |
| F2R | ASPIRIN | NA | NA | NA |
| F2R | BLEOMYCIN | BLEOMY-CIN | BLEOMYCIN | Ambiguous |
| F2R | THROMBIN | NA | NA | NA |
| F2RL1 | CLARITHROMYCIN | CLARIT-HROMYC-IN | CLARITHROMYCIN | Definite |
| F2RL1 | DOXYCYCLINE | DOXYCY-CLINE | DOXYCYCLINE | Ambiguous |
| F2RL1 | DOXYCYCLINE ANHYDROUS | NA | NA | NA |
| F2RL1 | ERYTHROMYCIN | ERYTHR-OMYCIN | ERYTHROMYCIN | Definite |
| F2RL1 | MINOCYCLINE | MINOCY-CLINE | MINOCYCLINE | Definite |
| F2RL1 | TETRACYCLINE | TETRAC-YCLINE | TETRACYCLINE | Definite |
| F5 | THROMBIN | NA | NA | NA |

*Continued on next page*

Table S3 – *Continued from previous page*

| Gene | Drug Name | Score | Type/Mechanism | Approval |
|---|---|---|---|---|
| F8 | THROMBIN | NA | NA | NA |
| F9 | DEXAMETHASONE | DEXAME-THASONE | DEXAMETHASONE | Definite |
| F9 | HYDROCORTISONE | HYDROC-ORTISONE | HYDROCORTISONE | Definite |
| F9 | HYDROCORTISONE BUTYRA-TE | NA | NA | NA |
| F9 | HYDROGEN PEROXIDE | NA | NA | NA |
| F9 | LEVOTHYROXINE | LEVOTH-YROXINE | LEVOTHYROXINE | Definite |
| FAAH | ACETAMINOPHEN | ACETAM-INOPHEN | ACETAMINOPHEN | Definite |
| FAAH | ARIPIPRAZOLE | ARIPIP-RAZOLE | ARIPIPRAZOLE | Definite |
| FAAH | IBUPROFEN | IBUPRO-FEN | IBUPROFEN | Definite |
| FAAH | IBUPROFEN, SODIUM SALT | NA | NA | NA |
| FAAH | PROPOFOL | PROPOF-OL | PROPOFOL | Definite |
| FAAH | QUETIAPINE FUMARATE | NA | NA | NA |
| FABP1 | DEXAMETHASONE | DEXAME-THASONE | DEXAMETHASONE | Definite |
| FAS | ASPIRIN | NA | NA | NA |
| FAS | ATORVASTATIN CALCIUM TRIHYDRATE | NA | NA | NA |
| FAS | FLUOROURACIL | NA | NA | NA |
| FAS | THROMBIN | NA | NA | NA |
| FCER1G | ASPIRIN | NA | NA | NA |
| FCGR3A | PENICILLIN G POTASSIUM | PENICI-LLIN G POTASS-IUM | PENICILLIN G POTASSIUM | Definite |
| FCGR3A | SODIUM CHLORIDE | SODIUM CHLORIDE | SODIUM CHLORIDE | Definite |
| FCGR3B | PENICILLIN G POTASSIUM | PENICI-LLIN G POTASS-IUM | PENICILLIN G POTASSIUM | Definite |
| FCGR3B | PROGESTIN | NA | NA | NA |
| FCGR3B | SODIUM CHLORIDE | SODIUM CHLORIDE | SODIUM CHLORIDE | Definite |
| FDPS | ALENDRONATE SODIUM | ALENDR-ONATE SODIUM | ALENDRONATE SODIUM | Definite |
| FDPS | RISEDRONIC ACID | RISEDR-ONIC ACID | RISEDRONIC ACID | Definite |
| FEN1 | DEMECLOCYCLINE | DEMECL-OCYCLINE | DEMECLOCYCLINE | Definite |
| FEN1 | DOXYCYCLINE | DOXYCY-CLINE | DOXYCYCLINE | Ambiguous |
| FEN1 | DOXYCYCLINE ANHYDROUS | NA | NA | NA |
| FEN1 | EPINEPHRINE | EPINEP-HRINE | EPINEPHRINE | Definite |
| FEN1 | NOREPINEPHRINE | NOREPI-NEPHRINE | NOREPINEPHRINE | Definite |
| FGF2 | ASPIRIN | NA | NA | NA |
| FGF2 | ATORVASTATIN CALCIUM TRIHYDRATE | NA | NA | NA |
| FGF2 | CAFFEINE | CAFFEINE | CAFFEINE | Definite |
| FGF2 | PHENYLEPHRINE | PHENYL-EPHRINE | PHENYLEPHRINE | Definite |
| FGF2 | TRIAMCINOLONE | TRIAMC-INOLONE | TRIAMCINOLONE | Definite |
| FGFR2 | FLUOROURACIL | NA | NA | NA |
| FGFR4 | FLUOROURACIL | NA | NA | NA |
| FKBP5 | VENLAFAXINE | VENLAF-AXINE | VENLAFAXINE | Definite |
| FOLH1 | CLAVULANIC ACID | NA | NA | NA |
| FOLH1 | MERCAPTOPURINE | NA | NA | NA |
| FOLR1 | DEXAMETHASONE | DEXAME-THASONE | DEXAMETHASONE | Definite |
| FOS | THROMBIN | NA | NA | NA |
| FOXO1 | FLUOROURACIL | NA | NA | NA |
| FPGS | FLUOROURACIL | NA | NA | NA |

*Continued on next page*

Table S3 – *Continued from previous page*

| Gene | Drug Name | Score | Type/Mechanism | Approval |
|---|---|---|---|---|
| FPR1 | PENICILLIN G POTASSIUM | PENICI-LLIN G POTASS-IUM | PENICILLIN G POTASSIUM | Definite |
| FSIP1 | ASPIRIN | NA | NA | NA |
| FTO | MERCAPTOPURINE | NA | NA | NA |
| G6PD | ACETAMINOPHEN | NA | NA | NA |
| G6PD | ASCORBIC ACID | NA | NA | NA |
| G6PD | CEFTRIAXONE | CEFTRI-AXONE | CEFTRIAXONE | Definite |
| G6PD | CHLORAMPHENICOL | NA | NA | NA |
| G6PD | CIPROFLOXACIN | CIPROF-LOXACIN | CIPROFLOXACIN | Definite |
| G6PD | DIAZEPAM | DIAZEPAM | DIAZEPAM | Definite |
| G6PD | EPINEPHRINE | EPINEP-HRINE | EPINEPHRINE | Definite |
| G6PD | ERYTHROMYCIN | ERYTHR-OMYCIN | ERYTHROMYCIN | Definite |
| G6PD | LIDOCAINE | LIDOCA-INE | LIDOCAINE | Definite |
| G6PD | METOCLOPRAMIDE | METOCL-OPRAMI-DE | METOCLOPRAMIDE | Definite |
| G6PD | METOCLOPRAMIDE HYDROC-HLORIDE | NA | NA | NA |
| G6PD | NALIDIXIC ACID | NA | NA | NA |
| G6PD | NITROFURANTOIN | NITROF-URANTOIN | NITROFURANTOIN | Definite |
| G6PD | NITROFURANTOIN, MACROC-RYSTALS | NA | NA | NA |
| G6PD | OFLOXACIN | OFLOXA-CIN | OFLOXACIN | Definite |
| G6PD | OMEPRAZOLE | OMEPRA-ZOLE | OMEPRAZOLE | Definite |
| G6PD | OXYMETAZOLINE | OXYMET-AZOLINE | OXYMETAZOLINE | Definite |
| G6PD | OXYMETAZOLINE HYDROC-HLORIDE | NA | NA | NA |
| G6PD | PENICILLIN G POTASSIUM | PENICI-LLIN G POTASS-IUM | PENICILLIN G POTASSIUM | Definite |
| G6PD | PHENAZOPYRIDINE | PHENAZ-OPYRID-INE | PHENAZOPYRIDINE | Definite |
| G6PD | POTASSIUM CHLORIDE | NA | NA | NA |
| G6PD | PROGESTIN | NA | NA | NA |
| G6PD | SODIUM CHLORIDE | NA | NA | NA |
| G6PD | SULFADIAZINE | SULFAD-IAZINE | SULFADIAZINE | Definite |
| G6PD | SULFAMETHOXAZOLE | SULFAM-ETHOXA-ZOLE | SULFAMETHOXAZOLE | Definite |
| G6PD | TRIAMCINOLONE | TRIAMC-INOLONE | TRIAMCINOLONE | Definite |
| G6PD | TRIMETHOPRIM | TRIMET-HOPRIM | TRIMETHOPRIM | Definite |
| GABRA1 | DIAZEPAM | DIAZEPAM | DIAZEPAM | Definite |
| GABRA1 | ESZOPICLONE | ESZOPI-CLONE | ESZOPICLONE | Definite |
| GABRA1 | FLURAZEPAM | FLURAZ-EPAM | FLURAZEPAM | Definite |
| GABRA1 | FLURAZEPAM HYDROCHLO-RIDE | NA | NA | NA |
| GABRA1 | PHENYTOIN | PHENYT-OIN | PHENYTOIN | Definite |
| GABRA1 | PROPOFOL | PROPOF-OL | PROPOFOL | Definite |
| GABRA1 | TEMAZEPAM | TEMAZE-PAM | TEMAZEPAM | Definite |
| GABRA1 | TRIAZOLAM | TRIAZO-LAM | TRIAZOLAM | Definite |
| GABRA1 | ZALEPLON | ZALEPLON | ZALEPLON | Definite |
| GABRA1 | ZOLPIDEM | ZOLPIDEM | ZOLPIDEM | Definite |
| GABRA1 | ZOLPIDEM TARTRATE | NA | NA | NA |
| GABRA2 | DIAZEPAM | DIAZEPAM | DIAZEPAM | Definite |

*Continued on next page*

Table S3 – *Continued from previous page*

| Gene | Drug Name | Score | Type/Mechanism | Approval |
|---|---|---|---|---|
| GABRA2 | ESZOPICLONE | ESZOPI-CLONE | ESZOPICLONE | Definite |
| GABRA2 | FLURAZEPAM | FLURAZ-EPAM | FLURAZEPAM | Definite |
| GABRA2 | FLURAZEPAM HYDROCHLO-RIDE | NA | NA | NA |
| GABRA2 | PROPOFOL | PROPOF-OL | PROPOFOL | Definite |
| GABRA2 | TEMAZEPAM | TEMAZE-PAM | TEMAZEPAM | Definite |
| GABRA2 | TRIAZOLAM | TRIAZO-LAM | TRIAZOLAM | Definite |
| GABRA2 | ZOLPIDEM | ZOLPIDEM | ZOLPIDEM | Definite |
| GABRA2 | ZOLPIDEM TARTRATE | NA | NA | NA |
| GABRA3 | DIAZEPAM | DIAZEPAM | DIAZEPAM | Definite |
| GABRA3 | ESZOPICLONE | ESZOPI-CLONE | ESZOPICLONE | Definite |
| GABRA3 | FLURAZEPAM | FLURAZ-EPAM | FLURAZEPAM | Definite |
| GABRA3 | FLURAZEPAM HYDROCHLO-RIDE | NA | NA | NA |
| GABRA3 | PROPOFOL | PROPOF-OL | PROPOFOL | Definite |
| GABRA3 | TEMAZEPAM | TEMAZE-PAM | TEMAZEPAM | Definite |
| GABRA3 | TRIAZOLAM | TRIAZO-LAM | TRIAZOLAM | Definite |
| GABRA3 | ZOLPIDEM | ZOLPIDEM | ZOLPIDEM | Definite |

# SUPPLEMENTARY TABLE S3: GENE DESCRIPTIONS AND RESPONSE DIRECTION

Table S4: **Gene Descriptions for spaceflight-flown drug associated genes.**

| Gene | Gene Description | Reference / Link | Direction |
|---|---|---|---|
| ABL1 | Involved in signaling pathways that regulate cell growth, being an essential target for drugs that inhibit kinase activity | NCBI Gene 25 | Differentially Expr. |
| ADRB3 | Regulates fat metabolism and thermogenesis | 10.1186/1471-2393-7-S1-S14 | Differentially Expr. |
| APOE | Plays a role transporting lipids also it has been associated with Alzheimer's disease | 10.1186/s13024-020-0358-9 | Differentially Expr. |
| ATF4 | Mediates cellular stress responses | 10.1016/j.tem.2017.07.003 | Differentially Expr. |
| B4GALT2 | Participates in the glycosylation of proteins and lipids | 10.1002/cpt.401 | Differentially Expr. |
| BAX | Involved in the regulation apoptosis, having a significant role its direct pharmacological modulation in human diseases | 10.1016/j.tips.2021.11.001 | Differentially Expr. |
| CHRM3 | Prevents the engagement of beta-arrestin-2 with odorant receptors | MGI:88398 | Differentially Expr. |
| CREBBP | Vital for central nervous system development | 10.1016/j.bcp.2004.05.035 | Differentially Expr. |
| CYP1B1 | Essential for drug efficacy also activates procarcinogens | SSN 0163-7258 | Downregulated |
| CYP2D6 | Responsible of the metabolization of different drugs | 10.1007/s00210-003-0832-2 | Downregulated |
| CYP3A43 | Responsible of the metabolization of different drugs | 10.1007/s00210-003-0832-2 | Upregulated |
| ERCC1 | Participates in the nucleotide excision repair (NER) pathway | 10.4161/cc.26309 | Differentially Expr. |
| FOS | Principal role in gene regulation | HGNC:3796 | Differentially Expr. |
| FTO | Plays a role in the regulation of energy balance also is involved in suppressing tumor growth | 10.1016/j.trecan.2022.02.010 | Differentially Expr. |
| GCLC | Crucial for glutathione synthesis and cellular stress response | HGNC:4311 | Upregulated |
| GGT1 | Principal role in glutathione and drug metabolism | 10.3389/fphar.2022.873792 | Upregulated |
| GLP1R | Maintains the glucose levels and promotes $\beta$ cell proliferation | HGNC:4324 | Differentially Expr. |
| GNAS | Mediates diverse hormones responses | NCBI Gene 2778 | Differentially Expr. |
| GSR | Related to glutathione synthesis influencing the development of drug | NCBI Gene 2936 | Differentially Expr. |
| GSTP1 | Plays a critical role protecting the cells through apoptosis resistance | 10.3892/ijo.2020.4979 | Differentially Expr. |
| GTF2B | Fundamental for the transcription initiation and gene expression | 10.1016/j.drudis.2006.11.012 | Differentially Expr. |
| HRH1 | Mediates various responses including antihistamines and allergic activity | NAID 500001363945 | Differentially Expr. |
| HSPA5 | Involved in protein folding, essential for the development of drugs | 10.1155/2019/2706783 | Differentially Expr. |
| HSPA8 | Involved in protein folding, essential for the development of drugs | 10.1155/2019/2706783 | Differentially Expr. |
| IMPDH1 | Fundamental for metabolism of mycophenolic acid | NCBI Gene 3614 | Differentially Expr. |
| JUN | Important for transcriptional regulation | HGNC:6205 | Differentially Expr. |
| JUNB | Important for transcriptional regulation | HGNC:6205 | Differentially Expr. |
| MECP2 | Controls the process of brain development | 10.3390/ijms20184577 | Differentially Expr. |
| MVK | A key in cholesterol metabolism and isoprenoid synthesis | 10.1016/j.bbadis.2010.04.001 | Upregulated |
| PIK3R2 | A role in the PI3K signaling pathway involved in cell growth | 10.7554/elife.76183 | Differentially Expr. |
| PLOD1 | Associated in lysyl hydroxylation and implicated in tissues disorders | 10.1155/2020/3418398 | Differentially Expr. |
| PRDX1 | An abundant antioxidant that protect cells from oxidative stress | 10.4161/cc.8.24.10242 | Differentially Expr. |
| PTH1R | A calcium regulator involved in bone health | 10.1210/endrev/bnab005 | Differentially Expr. |
| RAD52 | An important role in DNA repair and it is unique among the pharmacological targets to inhibit the growth of BRCA-deficient cancer | 10.1016/j.dnarep.2022.10342-1 | Differentially Expr. |
| RPS19 | A primary function for ribosome assembly | 10.4049/jimmunol.1602057 | Differentially Expr. |
| SCN9A | An important role in controlling pain sensation | 10.1097/j.pain.000000000000-1807 | Differentially Expr. |
| SDHB | A role in the mitochondrial electron transport providing subunits of the succinate dehydrogenase | 10.1158/1078-0432.CCR-19-2335 | Differentially Expr. |
| SLC19A1 | Involved in transporting folates and anti-folates agents into cells | 10.1097/FPC.0b013e32833e-ca92 | Downregulated |
| SLC23A2 | Regulates vitamin C distribution encoding two required transporters | 10.1016/B978-0-12-401717-7.00056-3 | Downregulated |

*Continued on next page*

Table S4 – *Continued from previous page*

| **Gene** | **Gene Description** | **Reference / Link** | **Direction** | |
|---|---|---|---|---|
| SLC29A2 | Nucleoside transport at the basolateral membrane of kidney cells | HGNC:11004 | Upregulated | |
| SOD1 | Protects cells from oxidative stress | NCBI Gene 6647 | Differentially Expr. | |
| STS | Influences as drug transporter and metabolizing steroid sex hormones into the active free steroid | 10.1080/13543776.2021.19-10237 | Differentially Expr. | |
| TGM2 | Acts in multiple cellular processes as adhesion, growth, survival and apoptosis; has been associated with fibrosis and inflammatory responses | 10.1016/B978-0-12-394305-7.00001-X | Differentially Expr. | |
| TMEM167A | May play a role in transporting different molecules across membranes | NCBI Gene 153339 | Differentially Expr. | |
| UGT1A4 | Expressed in the liver and bile ducts is responsible for glucuronidation of drugs | 10.1097/FPC.0b013e328333-1637 | Upregulated | |
| WRN | Recognized for DNA Helicase Activity, DNA repair and telomere maintenance | 10.3389/fgene.2014.00441 | Differentially Expr. | |
| ZBTB22 | Participates in DNA damage response and cell apoptosis | 10.1016/j.isci.2023.106318 | Differentially Expr. | |
